# Small molecule-mediated inhibition of the oxidoreductase ERO1A restrains aggressive breast cancer by impairing VEGF and PD-L1 in the tumor microenvironment

**DOI:** 10.1038/s41419-025-07426-1

**Published:** 2025-02-17

**Authors:** Ersilia Varone, Michele Retini, Alessandro Cherubini, Alexander Chernorudskiy, Alice Marrazza, Andrea Guidarelli, Alfredo Cagnotto, Marten Beeg, Marco Gobbi, Stefano Fumagalli, Marco Bolis, Luca Guarrera, Maria Chiara Barbera, Chiara Grasselli, Augusto Bleve, Daniele Generali, Manuela Milani, Michele Mari, Mario Salmona, Giovanni Piersanti, Giovanni Bottegoni, Massimo Broggini, Yvonne M. W. Janssen-Heininger, Jaehyung Cho, Orazio Cantoni, Ester Zito

**Affiliations:** 1 Istituto di Ricerche Farmacologiche Mario Negri IRCCS, Milan, Italy; 2 Department of Biomolecular Sciences, University of Urbino Carlo Bo, Urbino, Italy; 3 Bioinformatics Core Unit, Institute of Oncology Research (IOR), Bellinzona, Switzerland; 4 U.O. Patologia Mammaria e Tumori Cerebrali, Azienda Socio-Sanitaria Territoriale, Cremona, Italia; 5 Institute of Clinical Sciences, University of Birmingham, Edgbaston, B15 2TT Birmingham UK; 6 Departments of Pathology and Laboratory Medicine, University of Vermont College of Medicine, Burlington, VT USA; 7 Division of Hematology, Department of Medicine and Department of Pathology and Immunology, Washington University School of Medicine, St. Louis, USA; 8 Present Address: School of Medicine, Taizhou University, Taizhou, 318000 Zhejiang China

**Keywords:** Breast cancer, Cancer

## Abstract

Cancer cells adapt to harsh environmental conditions by inducing the Unfolded Protein Response (UPR), of which ERO1A is a mediator. ERO1A aids protein folding by acting as a protein disulfide oxidase, and under cancer-related hypoxia conditions, it favors the folding of angiogenic VEGFA, leading tumor cells to thrive and spread. The upregulation of ERO1A in cancer cells, oppositely to the dispensability of ERO1A activity in healthy cells, renders ERO1A a perfect target for cancer therapy. Here, we report the upregulation of ERO1A in a cohort of aggressive triple-negative breast cancer (TNBC) patients in which ERO1A levels correlate with a higher risk of breast tumor recurrence and metastatic spread. For ERO1A target validation and therapy in TNBC, we designed new ERO1A inhibitors in a structure-activity campaign of the prototype EN460. Cell-based screenings showed that the presence of the Micheal acceptor in the compound is necessary to engage the cysteine 397 of ERO1A but not sufficient to set out the inhibitory effect on ERO1A. Indeed, the ERO1 inhibitor must adopt a non-coplanar rearrangement within the ERO1A binding site. I2 and I3, two new EN460 analogs with different phenyl-substituted moieties, efficiently inhibited ERO1A, blunting VEGFA secretion. Accordingly, in vitro assays to measure ERO1A engagement and inhibition confirmed that I2 and I3 bind ERO1A and restrain its activity with a IC50 in a low micromolar range. EN460, I2 and I3 triggered breast cancer cytotoxicity while specifically inhibiting ERO1A in a dose-dependent manner. I2 more efficiently impaired cancer-relevant features such as VEGFA secretion and related cell migration. I2 also acted on the tumor microenvironment and viability of xenografts and syngeneic TNBC. Thus, small molecule-mediated ERO1A pharmacological inhibition is feasible and promises to lead to effective therapy for the still incurable TNBC.

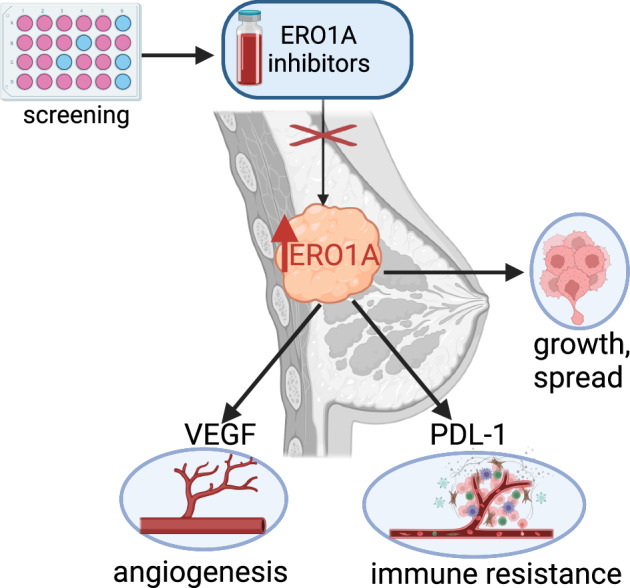

## Introduction

Harsh conditions in cancer, such as hypoxia, shortage of nutrients, and oncogenic mutations give rise to Endoplasmic Reticulum (ER) stress. This activates a homeostatic response, the Unfolded Protein Response (UPR), which rescues ER homeostasis through corrective measures, while boosting cancer cell fitness [1, 2]. UPR leads cancer cells to thrive and spread through a cell-autonomous mechanism: for example, by upregulating enzymes and molecular chaperones involved in protein folding, i.e., Endoplasmic Reticulum Oxidoreductin 1 A (ERO1A), and through a cell non-autonomous mechanism regulating Vascular Endothelial Growth Factor (VEGF), the related angiogenesis, and tumor microenvironment [3–6].

ERO1A is a Flavin Adenine Dinucleotide (FAD)-containing disulfide oxidase involved in an electron relay with Protein Disulfide Isomerase (PDI), which culminates in disulfide bond formation within nascent proteins [7], and affects metabolism [8] and mitochondrial bioenergetics [9]. In mammals, ERO1A and its paralogue ERO1B share 65% of amino acid sequence identity and an overlapping function. However, mice lacking ERO1A (and its paralogue ERO1B) are viable and fertile and do not show any overt phenotype [10]. Thus, unlike in yeast, where ERO1 activity is essential, in mammals, loss of ERO1 activity is well compensated by other oxido-reductive enzymes such as PRDX4, GPX7, and GPX8, which support protein disulfide bond formation [11–14]. ERO1A is upregulated in many types of cancer, and its upregulation correlates with a worse clinical outcome [15–17]. This upregulation is part of the adaptive UPR, aiding cancer cells with protein folding in hypoxia, which impairs post-translational disulfide bond formation, thus boosting cell fitness [1, 18–20].

Triple-negative breast cancer (TNBC) is the most aggressive type among breast cancers, with an incidence between 12–17% [21]. TNBC holds a dismal prognosis because of the shortage of effective therapeutics, urging the discovery of new therapeutic options [22]. Remarkably, inhibition of IRE1 α, a UPR branch, enhances the response to anti-angiogenic VEGF-target therapy in TNBC [6]. Likewise, ERO1A improves VEGF folding under hypoxic conditions, favoring cancer-related angiogenesis and metastasis in TNBC [5, 23, 24]. Accordingly, ERO1A genetic ablation reduces VEGF folding and affects its glycosylation, restraining angiogenesis and metastasis while it enhances the response to chemotherapy and VEGF-target therapy [16, 23, 25, 26].

Thus, while dispensable in healthy cells, ERO1A improves the survival of TNBC cells. This difference in the selective need for ERO1A activity between healthy and TNBC makes ERO1A an ideal target for pharmacological inhibition in this cancer type.

EN460 is an ERO1A inhibitor; this small molecule features a Michael acceptor moiety, capable of forming a covalent adduct with an essential cysteine residue of ERO1A, hence trapping ERO1A in a reduced state and impeding the relay of electrons to PDI [27]. Despite the specificity of EN460 toward ERO1A, its conjugated double bond, which may react with a range of thiol-containing compounds, is a reason for concern for potential off-target effects and toxicity in cells, thus excluding EN460 from clinical use. Nonetheless, the favorable target engagement profile of EN460 in vivo prompted us to carry out a characterization and lead compound optimization campaign of EN460.

Because of the high ERO1A expression levels in aggressive TNBC and the limited therapeutic option of this cancer type, we investigated ERO1A selective inhibition there. We concluded that ERO1A pharmacological inhibition with EN460 and new analogs yields anticancer efficacy, restraining the fitness of TNBC while not showing overt toxicity at the level of the organism.

## Materials And Methods

### EN460 and analogues I1-16

EN460 and analogues I1-I16 were synthesized, and the experimental procedures for synthesis, the NMR spectra, HPLC analysis, high-resolution mass spectrometry (ESI-TOF), yield, melting points, and purity of compounds tested are attached in the Supplementary Methods. All compounds were diluted in 25 μM DMSO and diluted at the final concentrations indicated for in vitro and *in cell* assays.

### Compounds I17-23

Compounds I17-23 are commercially available and were purchased from Enamine (Enamine Ltd., Kiev) with a declared purity of 90%.

### Molecular docking studies

Ligand docking simulations were carried out with the Covalent Docking protocol as implemented in Schrödinger 2023-1 (Schrödinger LLC, New York, USA). Unless otherwise specified, default parameters were used. The coordinates of the enzyme in complex with FAD (PDB: 3AHQ) [28] were processed with the Protein Preparation routine. The coordinates of the co-crystallised cofactor were used to define the size and the position of the binding box and then the ligand was deleted from the system. The software was instructed to simulate a Michael addition reaction between Cys397 and the third atom of a region of the ligand matching ocC=CC = N SMARTS pattern. The best scoring complex was retained and subjected to full atom minimisation using Desmond as implemented in Schrödinger. The system was solvated in an orthorhombic box of TIP3P water molecules whose dimensions were automatically calculated, assigning a 12 Å buffer in each direction. The system was neutralised considering a 0.15 M salt concentration. The system encompassed 44410 atoms. The system was minimized by means of a 100 ps Brownian motion simulation. All calculations were carried out using the OPLS4 force field [29]. Geometry optimization in solution was done at the DFT level of theory using B3LYP-D3 functional and 6-31 G** basis set as implemented in Jaguar (Schrödinger LLC). The solvent was modelled using the polarizable continuum model (PCM) [30].

### Expression and purification of ERO1A recombinant protein

A GST-SMT3-mouse ERO1A (residues 43–464) was expressed in the Rosetta DE3 bacteria (Novagen). After growth at 37 °C to an *A*_600_ of 0.6–0.8, the culture was shifted to 16 °C, and isopropyl-β-d-thiogalactoside was added to 0.5 mM for 24 h. Bacteria were harvested and resuspended in lysis buffer (50 mM Tris pH 8, 200 mM NaCl, Triton 0,2%) supplemented with protease inhibitor without EDTA. The lysate was centrifuged for 30 min at 13000 rpm, and the supernatant was applied to GST trap Glutathione Sepharose 4B (GE17-0756-01, Merck) over night at 4 °C. The next day, after initial washes, Ulp protease (Z03691 GenScript) was added to a column containing GST trap column and incubated two hours at 4 °C to cleave the GST-SMT3 from the ERO1A. After further washes the flow-through containing ERO1A was collected. The concentration of the purified protein was determined spectroscopically at 280 nm, the presence of FAD was determined by measuring the fluorescence at 450ex and 535em.

### Mobility Shift Analysis of ERO1A redox status

Recombinant ERO1A (1 μM) was reacted with the inhibitors at the indicated concentrations before quenching the reaction with NEM (10 mM). To detect ERO1A redox status, samples were resolved by non-reducing SDS-PAGE (10%) and stained with Coomassie Blue.

### Kinetic assay of ERO1A activity

ERO1A (residues 43–464) at 200 nM was preincubated with the compounds (I) at different concentrations in buffer A (65 mM NaCl, 20 mM sodium phosphate buffer, pH7.4, 1 mM EDTA) for five minutes. Later, 20 μM bacterially expressed His-tagged PDIA1 [31], which was previously reduced with DTT and gel filtered by PD10 column, was added together with 0.1 units/ml horseradish peroxidase (Worthington) and 5 μm Amplex Ultra Red (AUR; Invitrogen) in a 96-well black round bottom plate. The reaction was read kinetically at 535 nm excitation and 590 nm emission on a TECAN Infinite M Nano+ fluorescent plate reader. The effect of the ERO1A inhibitors was evaluated as inhibition of the initial rate of reaction (first 10 min), and the corresponding IC50 was determined using Prism 10 (GraphPad).

### Tumor cell lines

Cells were kept in culture for no more than two weeks and routinely tested for mycoplasma infection. MDAMB231 cells used in this essay were selected from parental MDAMB231(#505366 from ATCC Frederick Cancer Tumor Repository, Maryland, USA) through passages in mice to enhance their tumorigenic and metastatic properties as described in [32]. MDAMB231 cells were infected with a lentiviral vector carrying the coding sequence of the synthetic firefly luciferase gene, luc2 (Photinus pyralis). Twenty-four hours after infection, cells were selected with blasticidin (5μg/mL). ERO1A KO MDAMB231 were as described elsewhere [16, 19, 23]. E0771 murine breast cancer cells were bought (940001-A from CH3 BioSystems). E0771 cells were transfected with ERO1-Lα CRISPR-Cas9 KO plasmids (SC-424456 for murine, Santa Cruz Biotechnology) with three target-specific guide RNAs (gRNA) of 20 nt. The plasmids were co-transfected with homology-directed repair HDR (SC-424456-HDR, Santa Cruz Biotechnology) plasmids, which led to the insertion of puromycin resistance gene and red fluorescent protein (RFP) gene. Cells were seeded 24 h before transfection in a six-well plate and transfected with Fugene HD (E2311, Promega) at a 1:3 DNA/reagent ratio as described in the manufacturer’s manual. 72 h after transfection, cells were detached and seeded in 10 cm Petri dish and puromycin (6 μg/mL) was added to select positive clones. The clones were subjected to Sanger sequencing and Immunoblot with a homemade polyclonal ERO1A antibody[33] and a monoclonal Anti Actin clone C4 (MAB1501, Millipore). ERO1 KO HeLa cells were generated by using CRISPR/Cas9 technology (Origene) following the manufacturer’s guidelines (already described in [34]).

### EN460 and I2 levels in cells by MALDI mass spectrometry

MDAMB231 cells were plated in six-well plates at 10^6^ cells per well. The next day subconfluent cells were treated with 5 and 20 μM of either EN460 or I2 in RPMI medium containing 1% FBS for 3 h. At the end of incubation, cells were washed with PBS, harvested in cold PBS supplemented with EDTA and frozen at −80°C. Cell lysis was achieved by repeating freeze/thaw cycles, and the remaining pellet fraction was removed by centrifugation at 16000 g for 5 minutes at 4 °C. Cell lysates were frozen and the next day diluted with acetonitrile (ACN, 50%, 1:2), incubated for 30 minutes at 4 °C with shaking, then centrifuged at 4000 rpm for 4 minutes. After extraction, supernatants were collected, frozen and lyophilized. The freeze-dried samples were then re-suspended in a minimal volume of ACN (50%) and used for the MALDI analysis: one μl of sample was mixed with the same volume of the matrix solution (HCCA as below) and spotted on the MALDI target plate with a standard solution of EN460 and I2.

### Hypoxic chamber

Human MDAMB231 and murine E0771 breast cancer cells were transferred to hypoxic chamber (Ruskinn Invivo2 400, UK) at 37°C and maintained in deoxygenated culture medium at the following gas concentrations: O_2_ 0.1%, CO_2_ 5% for 48 h. Control cells were maintained in standard culture medium in a normoxic incubator. Conditioned media were assayed for VEGF by ELISA.

### Motility assay

MDAMB231 and E0771 motility were assessed using modified Boyden chambers and gelatin-coated polycarbonate nucleopore filters (8 μm pore size). Cells were treated with EN460 and I2 at the concentrations indicated for 12 hours, then washed and resuspended in high glucose DMEM with 0.1% BSA at 1 × 10^6^/mL and added to the upper compartment of the chamber. Assays were carried out in 5% CO2 at 37 °C for 6 h. At the end of the incubation, filters were fixed and stained with Diff-Quik (Marz-Dade, Dundingen, Switzerland) to detect cells adhering to the lower surface, and migrated cells in ten high-power fields for each filter were counted.

### VEGF measurements in conditioned media

An expression plasmid encoding N-terminally FLAG-tagged human VEGF^121^ (VEGF 1116-1483 of NM_001171628.1) was described elsewhere and used to transfect HeLa cells [23]. After 24 h the cells were split into six multi-well dishes and treated with 20 μM of 23 different molecules (I1-I23) for 2 h. After 6 h of washout from the inhibitors, the conditioned media in low serum (1% FBS) was collected. FLAG M2 (monoclonal mouse anti-Flag M2 F3165, Sigma Aldrich) Western Blotting was assessed on the proteins from conditioned media to evaluate the secreted VEGF.

### Surface Plasmon Resonance (SPR) for evaluation of VEGFA secretion

The ProteOn XPR36 Protein Interaction Array system (Bio-Rad Laboratories, Hercules, CA) was used for these studies. FLAG-M2 (monoclonal mouse anti-Flag M2, F3165, Sigma Aldrich) was immobilized on the sensor chip (GLC, Bio-Rad) by amine coupling chemistry, whereas an activated/deactivated “empty” surface was prepared in a parallel lane of the same chip and used as reference. Conditioned media containing FLAG-tagged human VEGF^121^ from HeLa cells treated with the different compounds were then flowed over immobilized anti-Flag and the empty surface [35].

### Effect of inhibitors on ERO1A redox status in vivo

HeLa, MDAMB231 and E0771 cells were cultured in Dulbecco’s modified Eagle’s medium supplemented with 1% fetal bovine serum and plated the day before the experiment at 70% confluence in six-well dishes. Cells were challenged with DTT (10 mM) and/or the ERO1A inhibitors for 1-5 hours. At harvest, phosphate-buffered saline containing NEM (10 mM) was added and the cells were then kept on ice for 10 min in lysis buffer (50 mM Tris-HCl (pH 7.4), 150 mM NaCl, 1% Triton X-100, 0.1% SDS, 1% sodium deoxycholate, protease inhibitors, and 10 mM NEM). Protein concentrations were measured by BCA assay, and comparable amounts of protein were run on non-reducing SDS-PAGE and immunoblotted with polyclonal antiserum to ERO1A [33]. Full and uncropped western blots are reported in Supplemental Material.

### VEGFA ELISA

Secreted VEGF was measured in the conditioned media of 5 × 10^6^ MDAMB231 and E0771 cells treated with EN460 and I2 or vehicle for 16 h (overnight treatment), after the media was changed in RPMI with 1% FBS and cells underwent hypoxia (O_2_ at 0,1%) for further 24 h. VEGF in MDAMB231 and E0771 conditioned media was assessed with a human VEGF Quantikine ELISA Kit (DVE00, R&D Systems) and mouse VEGF Quantikine ELISA Kit (MMV00, R&D Systems), respectively. Intracellular proteins were quantified by BCA. For measurements of VEGFA associated with MDAMB231 and E0771 tumors, tumors were weighed, 50 mg of each isolated, and lysed in PBS with protease inhibitor and without EDTA. After homogenization, samples were frozen overnight and, the day after, underwent cycles of freezing and thawing. The insoluble material was removed after centrifugation at high speed, and the supernatant was quantified by BCA. An amount of 190 μg of proteins was assessed by the aforementioned ELISA kits.

### MTS

Six thousand cells (MDAMB231 and E0771)/well were plated in 96-well plates and cultured in media containing 1% FBS. Cells were challenged with different concentrations of compounds and after 24 hours incubated in MTS [3-(4,5-dimetiltiazolo-2-il)-5-(3-carbossimetossifenil)-2-(4-solfofenil)-2H-tetrazolio] and PMS (Phenazine methosulfate) as indicated in CellTiter 96® Aqueous Non-Radioactive Cell Proliferation Assay (Promega). Acquisitions were made by TECAN infinite M200 using excitation wavelengths at 490 nm.

### Evaluation of apoptosis in tumor cells by flow cytometry analysis

Flow cytometry was performed on 5 × 10^4^ cells cultured in media with 1% FBS and treated for 6 (T1) and 14 (T2) h (overnight treatment) with the inhibitors. Cells were labeled with Annexin V-FITC and propidium iodide (Invitrogen) at the acquisition rate of 300 events per second, using a CytoFLEX LX flow cytometer (Beckman Coulter, Brea, CA, USA).

### Determination of GSH content in cells

Subconfluent cells were plated in 10 cm plates and incubated overnight. The following day compounds added to each well at the indicated concentrations. After 6 hours of exposure of the compounds to cells, the cells were harvested by trypsin, and after washing with PBS the cell pellets were lysed using 10 mM HCl. The protein content of each lysate was quantified by BCA, and proteins were precipitated with 0.1% w/v 5-sulfosalicylic acid. Samples were added to 96-well plates and neutralized using a stock buffer of 143 mM NaH_2_PO_4_ containing 6.3 mM EDTA. 1.1 mM DTNB and 350 μM NADPH were added to each well. The absorbance at 412 nm was read in a Tecan reader after 5 min incubation. GSH concentrations were determined using a linear curve of a standard reduced GSH to obtain nmoles GSH per mg of protein.

### Animals

Eight- to ten-week-old female SCID and C57/BL6J mice were obtained from Charles River Laboratories (Calco, Italy) and maintained under specific-pathogen-free conditions. SCID mice were housed in isolated vented cages and handled using aseptic procedures. Animal studies are reported in compliance with the ARRIVE guidelines and conducted in conformity with the following laws, regulations and policies governing the care and use of laboratory animals: Italian Governing Law (D.lgs 26/2014, authorization 19/2008-A issued 6 March 2008 by Ministry of Health; authorization 896/2023-PR to E. Zito); Mario Negri Institutional Regulations and Policies providing internal authorization for people conducting animal experiments (Quality Management System Certificate—UNI EN ISO9001: 2008—registration number 6121); EU directives and guidelines (EEC Council Directive 2010/63/UE), and in line with Guidelines for the welfare and use of animals in cancer research [36].

### Formulation of EN460 and I2 for studies in mice

A 1:1 polyethylene glycol 400 (PEG400) (Merck, 8.17003.1000)/Cremophor EL (Sigma, C5135-500G) mixture was used to solubilize EN460 and I2 at a concentration of 10 mM and served as a vehicle control. EN460 and I2 were first suspended in PEG400, then Cremophor EL was added to the mixture, and the solution was heated at 80 °C until the compound completely dissolved. Compounds and vehicle were diluted 1:10 in physiological saline solution immediately before being injected into mice at 10 mg/kg.

### Breast tumor models and treatments

**Scheme 1:**
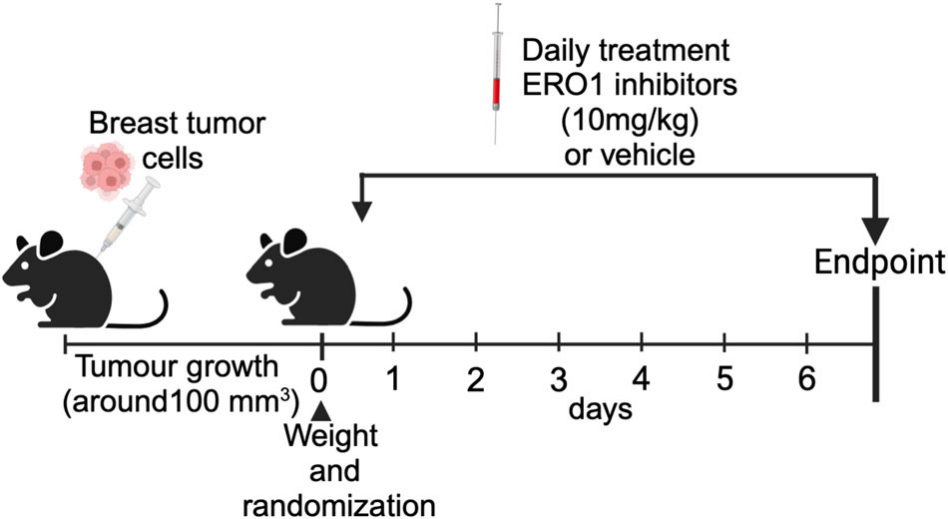
Scheme of mouse treatment with ERO1 inhibitors.

MDAMB231 and E0771 (1 × 10^6^) cells were subcutaneously inoculated into the right flank, located at the anterior lateral thoracic wall of SCID and C57/BL6J mice respectively. When the tumor reached an average volume of 100 mm^3^ mice were randomized based on tumor weight to receive EN460, I2 or vehicle by intraperitoneal injection once a day for six days (*N* = 8–12 in each group). The investigators were not blinded to the treatments. Tumor volume was measured by Vernier caliper and calculated according to the formula *D* *×* *d*^*2*^*/2*, where *D* is the largest diameter of the tumor and *d* the smallest one. MDAMB231 tumors were also quantified by bioluminescence imaging (BLI). Briefly, D-luciferin (150 mg/kg, i.p, Caliper Lifescience) injected mice were scanned after 10 minutes with IVIS Lumina Series III XRMS (Perkin Elmer). Images were analyzed with Living Image software (Perkin Elmer) and tumor burden was expressed as total flux (photons/sec). Mouse health and weight was monitored daily during the treatments. After one week, mice were sacrificed and primary tumors were collected and randomly selected for further analysis such as RNA sequencing, VEGF and MALDI imaging analysis (scheme 1 below).

E0771 were injected orthotopically (1 × 10^6^) into the mammary fat pad (m.f.p) of 20 C57BL/6 J female mice. When the primary tumor reached a volume around 100 mm^3^, mice were randomized and allocated to two groups of ten each to receive I2 or vehicle (Cremophor and PEG400). I2 was injected intraperitoneally (i.p.) once daily for 6 days at the dose of 10 mg/kg. Twenty-four hours after the sixth administration, mice were sacrificed. Tumors were resected and immediately analyzed by flow cytometry analysis for PD1 and PD-L1.

### Flow cytometry analysis of orthotopic breast tumors

Primary breast tumors derived from E0771 were cut into small pieces, disaggregated with 0.5 mg/ml Collagenase-IV (Sigma) and 40 ug/ml DNase-I in RPMI 1640 for 30 min at 37 °C, and filtered through a 70-μm strainer. A standardized red blood cell lysis protocol (ACK buffer, 5 min at RT) was then adopted. The resulting cells were resuspended in HBSS supplemented with 0.5% FBS. Staining was at 4 °C for 20 min using the following antibodies (Biolegend): CD45 BV650 (clone 104; dilution 0.6/100), CD11b PerCP-Cy5.5 (clone M1/70; 0.3/100), Ly6C BV421 (clone HK1.4; 0.6/100), Ly6G AF700 (clone 1A8; 0.6/100), F4/80 PE-Cy7 (clone BM8; 0.3/100), PD-L1 APC (clone 10 F.9G2; 1/100), CD3 BV605 (145-2C11; 0.6/100) CD4 AF700 (clone GK1.5; 0.6/100), CD8 BV785 (clone 53–6.7; 0.6/100), PD1 (clone 29.F1A12; 0.6/100). Cell viability was determined using LIVE/DEAD™ Fixable Near IR (876) Viability Kit (Invitrogen, L34981). Cells were detected using Cytoflex LX instrument (CytExpert software) and analyzed with Kaluza 2.0 software (Beckman Coulter). A traditional gating strategy was used to remove aggregates and dead cells. Among the CD45^+^ cells (leukocytes), monocytic (M-, CD11b^+^Ly6G^–^Ly6C^hi^) and granulocytic (PMN-, CD11b^+^Ly6G^+^Ly6C^lo^) myeloid-derived suppressor cells (MDSCs), and tumor-associated macrophages (TAM, CD11b^+^Ly6C^lo/–^F4/80^+^) were gated. Among the CD45^+^ cells (leukocytes), total T lymphocytes (CD3 + ) were gated and then the percentages of CD3 + CD4+ and CD3 + CD8+ lymphocyte subsets within the T cell population were determined. PD1 expression was measured in CD4+ and CD8+ cells while PD-L1 expression in MDSCs and TAM; their expressions were reported as geometric mean fluorescence intensity (gMFI).

### RNA sequencing analysis of breast tumors

RNA was extracted from MDAMB231 xenografts (five separate samples for each condition: vehicle, EN460- and I2-treated) with the Qiagen RNeasy kit. RNA concentration was measured with Qubit^TM^ RNA Broad Range Assay Kit (Invitrogen, Waltham, MA, USA) and RNA quality was established using 4200 Tapestation (Agilent Technologies, Santa Clara, CA, USA). Libraries were prepared through the TruSeq Stranded Total RNA protocol (Illumina, San Diego, CA, USA). RNA sequencing was run on a NextSeq 500 sequencer (Illumina). The overall quality of sequencing reads was evaluated using FastQC (v.0.11.9). Sequence alignments of total-RNA (stranded) to the hybrid reference [mouse genome (GRCm39) and human genome (GRCh38)] were done using STAR (v.2.7.9a) in two-pass mode. Gene expression was quantified at the gene level, using the hybrid comprehensive annotations made available by Gencode (m39.vM27 GTF File & hg38.v38 GTF File). Samples were adjusted for library size and normalized with the variance stabilizing transformation (vst) in the R statistical environment. Differential Expression Analysis was conducted using DESeq2 (v1.28.1) pipeline. Gene Set Enrichment Analysis was performed using the limma (v.3.44.3) package. Gene-set collections were retrieved from the Molecular Signature Database (MSigDB). *P*-values were corrected for multiple testing using the false discovery rate (FDR) procedure, with the significance threshold set to 0.05. Data were deposited at EMBL-EBI *Annotare database* (https://www.ebi.ac.uk/fg/annotare/) under the accession numbers: E-MTAB-14269 [37, 38].

### EN460 and I2 distributions in breast tumours by MALDI imaging

After treatment MDAMB231 tumours were collected, frozen in liquid nitrogen and stored at −80 °C before sectioning. Sections (14 µm thickness) were taken at −20 °C in a cryostat and mounted on steel plate MALDI targets (Opti-TOF High-Resolution TIS, Applied Biosystem) using a small paintbrush. Mounted tissue sections were subsequently placed under vacuum at 4 °C overnight and then stored at −20 °C until use. On the day of the experiment, mounted tissue sections were coated with a matrix solution of α-Cyano-4-hydroxycinnamic acid (HCCA, 15 mg/mL) dissolved in 60% acetonitrile/0.2% trifluoroacetic acid using a glass nebulizer. The plate was dried at room temperature for some minutes, then inserted in the MALDI-TOF mass spectrometer (4800 MALDI-TOF, Applied Biosystem). For a correct quantification of EN460 and I2 content in tumors, EN460 and I2 standards at known concentrations were loaded onto the plate and spiked on the tumor section. Images were acquired with the MALDI-TOF mass spectrometer operated in positive linear mode at a lateral resolution (raster) of 120 µm. Tissue View software was used to analyze and quantify EN460 and I2 in mouse tumours.

### Breast cancer patient and tissue collection

The tissue samples (breast cancers) were collected at U.O. “Patologia Mammaria e Tumori Cerebrali, Azienda Socio-Sanitaria Territoriale of Cremona, Italy” during normal clinical practice between 2005 and 2008. Human studies were approved by the local ethical committee of “Azienda Socio-Sanitaria Territoriale of Cremona” (RaLCTrVs1Ott_2000). The breast cancers were classified in Luminal A and TNB by the pathologist using H&E staining and molecular analysis. Leftover samples were used for the preparation of a human tissue microarrays (TMA) that was anonymized to the scientists who performed the analysis.

### Breast cancer tissue microarray

A TMA containing 52 cases of TNB and 48 cases of Luminal A tissues, was prepared using a computer-assisted Tissue Microarrayer Model TMA Galileo CK4500 (Integrated Systems Engineering srl, Milano, Italy). Cores of 1 or 0.6 mm were prepared from FFPE samples in a region defined by the pathologist on consecutive H&E-stained slices.

### ERO1A and Ki67 expression in breast cancers

Five-μm tissue tumor slide sections were baked at 70 °C for 40 minutes, deparaffinized with xylene and rehydrated through a graded series of ethanol solutions (100%, 95%, 85% and 75%). Tissue slides were then placed in citrate buffer pH 6 (Biocare medical, CB910M) and microwaved for fifteen minutes to induce epitope retrieval.

The slides were rinsed in 1% H_2_O_2_ for 10 minutes at room temperature (RT), then three times in PBS 0.01 M for 5 minutes at RT. Proteins were blocked with NGS 10%, Triton 0.3% in PBS 0.01 M for 1 h. The slides were incubated with anti-ERO1L antibody [EPR12474] (ab177156) 1:100 in overnight at 4 °C or with Anti-Ki67 antibody [SP6] ab16667 1:200 in PBS 0.01 M with NGS 1% overnight at 4 °C. The slides were washed three times in PBS 0.01 M for 5 minutes at RT and incubated with anti-rabbit biotinylated 1:200, NGS1% in PBS 0.01 M for 1 h at RT. Following a series of washes including 3 times in PBS 0.01 M for 5 minutes at RT and then, in TNT (0,04% Tween 20 in TBS 0,1 M) for 5 minutes at RT), the slides were incubated in blocking reagent (AKOYA Biosciences NEL701A001KT) for 1 h and 30 minutes at RT. The slides were washed 3 times in TNT for 5 minutes at RT and incubated with streptavidin-HRP 1:100 in blocking reagent (AKOYA Biosciences NEL701A001KT) for 30 minutes at RT. Then, the slides were rinsed three times in TNT for 5 minutes at RT, incubate with Fluorescein 1:300 in Amplification Diluent (AKOYA Biosciences NEL701A001KT) for 8 minutes at RT, washed 3 times in PBS 0.01 M for 5 minutes at RT and incubated with Hoechst 1ug/ml in PBS 0.01 M for 10 minutes at RT. Finally, the slides were washed two times in PBS 0,01 M for 5 minutes at RT, once in sterile water and mounted with Prolong gold. The slides were acquired by A1 confocal system by Nikon with 20X objective lens using a large view function and avoiding signal saturation. The signal intensity of ERO1A was calculated in the whole section area and expressed as mean pixel grey level, while the Ki67 signal was automatically segmented and calculated as the density of positive cells per mm^2^. Digital image analysis was done by originally developed ImageJ algorithms. Correlation plot between ERO1A and Ki67 was obtained with GraphPAD Prism 10.

### Statistics

Data were analyzed by Prism 10 (GraphPad). The statistical analysis employed, as well as N, were indicated in the figure legends except for dot plots.

## Results

### ERO1A expression is upregulated in TNBC and positively correlates with the extent of tumor proliferation

Previously, we reported that ERO1A is upregulated in different cancer types [15]. On the contrary, its paralogue ERO1B was not upregulated in tumors compared to the healthy adjacent tissue (Sup. Figure 1) suggesting the importance of ERO1A rather ERO1B in cancer fitness.

We also analyzed ERO1A expression in breast cancer and normal counterpart tissues from The Cancer Genome Atlas (TCGA) databank and found higher ERO1A levels in the more aggressive basal breast cancer subtype, including TNBC [15, 23].

**Fig. 1:**
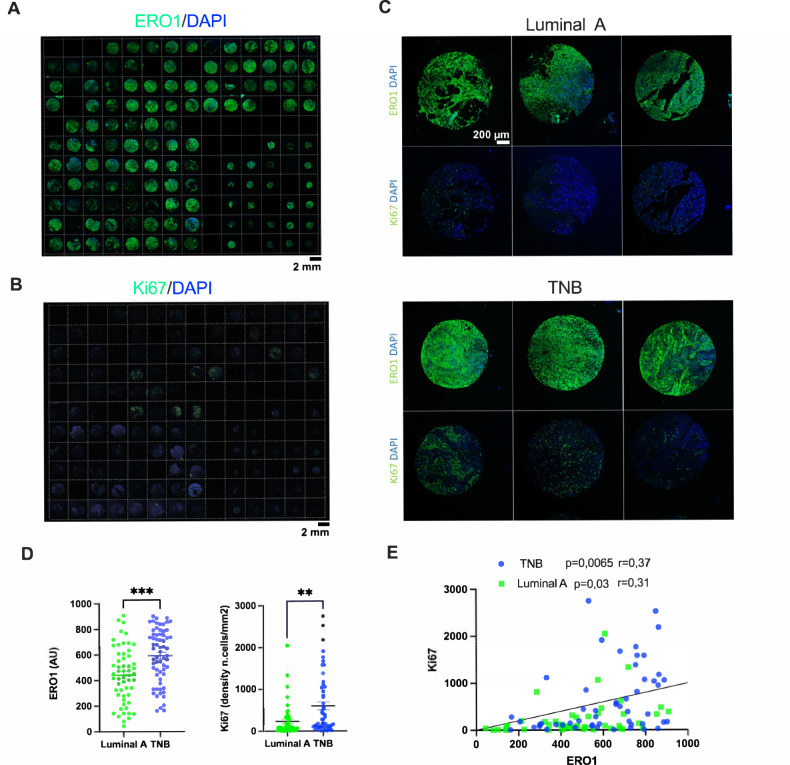
ERO1A expression in aggressive human breast cancer. **A** A tissue microarray TMA containing breast cancer samples, of which 48 were classified as Luminal A and 52 TNBC, was subjected to immunofluorescence with ERO1A (henceforth, ERO1); nuclei were stained with DAPI. **B** The same TMA was also subjected to immunofluorescence with Ki67. **C** Representative zoomed images of ERO1 and Ki67 staining in the indicated Luminal A and TNBC samples. **D** Dot plots representing the quantification of ERO1 and Ki67 staining (unpaired *t*-test). **E** Correlation plot between ERO1 and Ki67, the coefficient of correlation r and the *p* values were indicated on the graph.

To experimentally gain hints on ERO1A levels in TNBC, we exploited a tissue microarray (TMA) containing one hundred human breast cancer samples previously classified as Luminal A and TNBC. We proceeded with ERO1A immunofluorescence on them (Fig.1A). ERO1A expression levels were significantly higher in the more aggressive and poorly responsive to pharmacological treatment breast cancer type TNBC (Fig.1A–D). Notably, higher ERO1A levels positively correlated with that of the proliferation index Ki67 in both Luminal A and TNBC but more significantly in TNBC (Fig.1B–E). Furthermore, TNBC patients with higher expression of ERO1 had a higher risk of tumor recurrence and metastatic spread [23] indicating that ERO1A might be a biomarker for poor breast cancer prognosis and a target for cancer therapy.

### Chemistry of new ERO1A inhibitors

**Fig. 2:**
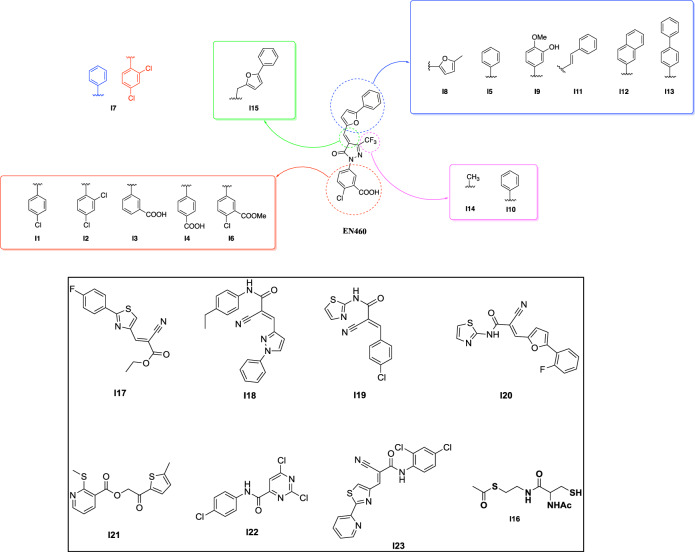
Structure of EN460 derivatives. Chemical structures of EN460. Each modified group in EN460 is boxed with a different color. The chemical substitutions of EN460 analogs (I1–14) are indicated and color- boxed. The phenyl of EN460 is depicted in red; analogs I1, I2, I3, I4 and I6 encompass the substitution of this group and are boxed in red. The phenyl-furan moiety of EN460 is depicted in blue, and the designated analogs with substitution of this group (I8-I15) are boxed in blue. The *α*, *β* unsaturated double bond is in green, and the analog with substitution of this group (I15) is also boxed in green. The side chain trifluoromethyl of pyrazolone is in purple and the analogs with substitution of this group (I14 and I10) are boxed in purple. Below, boxed in black, are commercially available analogs of EN460 putatively matching its activity profile while presenting a different chemical structure (I17–I23) and I16, a conjugate of N-acetylcysteine (NAC) and S-acetyl-β-mercaptoethylamine (SMEA).

Different analogs of the lead compound EN460 were synthesized to determine whether some of the four chemical groups indicated were critical for ERO1A inhibition, keeping the chemically active pyrazolone core fixed (I1-I15). The first class of compounds was designed to introduce different substituents from the phenyl-furan moiety of EN460 (depicted in blue) to modify its three-dimensional conformation and study how the reactivity of the *α*, *β* unsaturated double bond changed (I8, I5, I9, I11, I12 and I13). The second pharmacophoric modification of EN460 (green region) (I15) concerned the elimination of the *α*, *β* unsaturated double bond and might prove whether the Michael acceptor moiety is critical for drug activity. The third pharmacophoric modification (purple) led to the assessment of the reactivity of pyrazolone by replacing trifluoromethyl with methyl as an electron donating group or phenyl as an electron-withdrawing group (I14 and I10). The fourth set of compounds (red) (I1, I2, I3 I4 and I6), with different phenyl-substituted moieties bonded to the nitrogen of the pyrazolone core, might be helpful to assess the specificity of non-bonding interactions with ERO1A. To understand whether ERO1A cysteines might display promiscuous reactivity, we tested I16, a conjugate of N-acetylcysteine (NAC), and S-acetyl-cysteamine, which can bind cysteines [39]. Then, we conducted a ligand-based virtual screening exercise to select commercially available analogs of EN460 likely to match its activity profile despite having a different chemical structure. Through conformational sampling (MacroModel, as implemented in Schrodinger Drug Discovery software suite - Schrodinger, Inc., New York) we generated an energy minimum conformation of EN460 (geometric isomer *E*) in water and used it as a template. From Enamine (Enamine Ltd., Kiev) we retrieved a targeted library of compounds bearing groups (acrylamides, vinyl-ketones, etc) associated with covalent binding. When the screening was carried out (November 2021) the library encompassed 88,000 molecules. The library was processed and screened for 3D shape similarity with respect to the template, according to the standard protocol implemented in Schrodinger (default parameters were adopted). Each compound was assigned a similarity score, and the library was ranked accordingly. The top scoring compounds underwent visual inspection to verify that the position of the electrophile group matched that displayed in EN460. Thus, compounds I17-23 were selected and purchased (Fig. 2).

### Cell-based screening to identify effective ERO1A inhibitors

The formation of functional disulfides and the consequent secretion of VEGF^121^ was impaired in ERO1A-deficient cancer cells [19, 23]. Thus, VEGF^121^ secretion might be a valuable readout for measuring ERO1A inhibition.

**Fig. 3:**
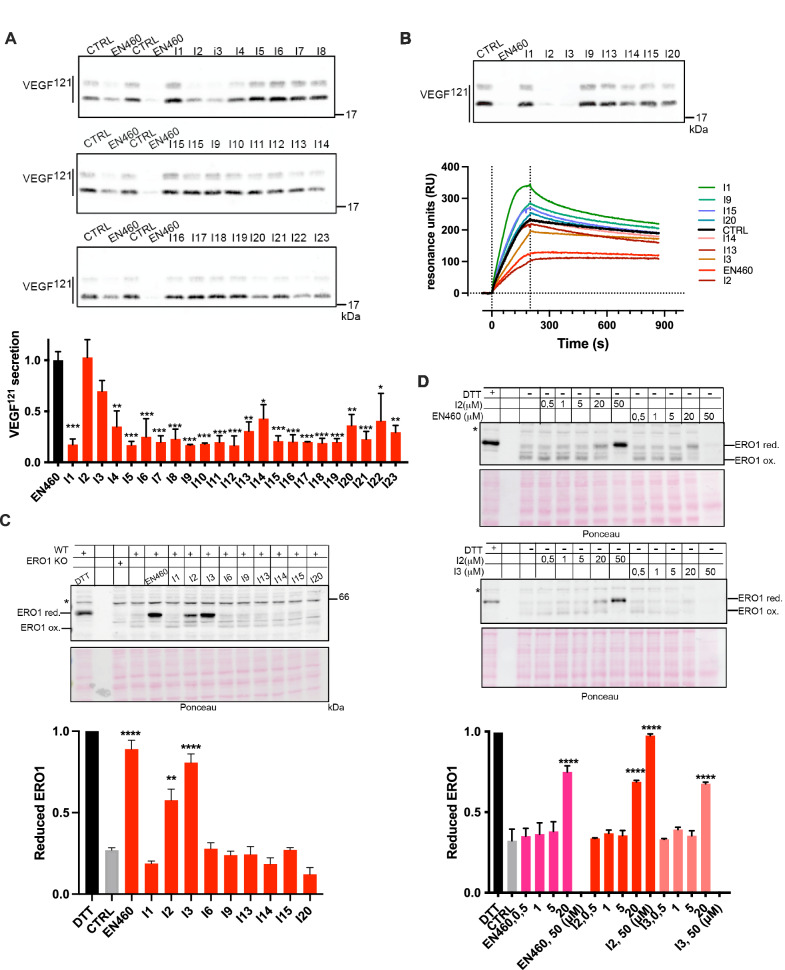
Effects of ERO1A inhibition on VEGF secretion and ERO1 redox state in vivo. **A** FLAG Immunoblot on conditioned media of HeLa cells transfected with FLAG-VEGF^121^ and treated with vehicle or inhibitors at 20 μM. Below is a bar graph representing the inhibition of VEGF secretion mediated by the different compounds when compared to the control; the ratio between the VEGF secreted from CTRL and the benchmark EN460 was arbitrarily set to 1 and calculated relatively to 1 for all the other compounds (*N* = 2, mean ± SD, One-Way ANOVA). **B** FLAG Immunoblot on conditioned media of HeLa cells transfected with FLAG-VEGF^121^ and treated with vehicle or inhibitors at 20 μM. Below, sensorgrams (time course of SPR signal in Resonance Units, RU) of FLAG-VEGF^121^ signal obtained by flowing the conditioned media over the immobilized FLAG M2 antibody (10 microgram/mL); these sensorgrams show the specific binding, already subtracted of the signal obtained on the reference surface with no antibody immobilized. **C** Non-reducing Immunoblot of endogenous ERO1A (henceforth, ERO1) in lysates of vehicle-treated HeLa cells or exposed to DTT, EN460 and the different inhibitors (I), as indicated. ERO1 red. stands for ERO1 reduced, ERO1 ox. for oxidized and * indicates a background band. ERO1 KO HeLa cells were loaded to indicate the background band. Ponceau stain indicated the protein loading control. The experiment was reproduced twice. Below is a bar graph representing reduced levels of ERO1A on total ERO1A (oxidized +reduced), which was arbitrarily set to 1 for the DTT-treated cells (mean ± SEM, One-Way ANOVA). **D** Non-reducing Immunoblot of endogenous ERO1A in lysates of vehicle-treated HeLa cells or exposed to DTT, and the indicated concentrations of EN460, I2 and I3. Representative experiment reproduced twice. Below is a bar graph representing reduced levels of ERO1A on total ERO1A (oxidized + reduced), which was arbitrarily set to 1 for the DTT-treated cells (mean ± SEM, One-Way ANOVA). Ponceau stain indicated the protein loading control.

Effective ERO1A inhibitors were assessed for reducing VEGF^121^ secretion in a VEGF^121^-FLAG-overexpressing HeLa cell line. The 23 compounds and the benchmark EN460 were added to the culture media at the concentration of 20 μM for 2 hours, then, after a washout of 6 hours, the conditioned media were collected and analyzed for VEGF content with Immunoblotting analysis and Surface Plasmon Resonance using an anti-FLAG antibody (Fig. 3A and B). Only I2 and I3 out of 23 compounds inhibited the secretion of VEGF in both assays in a quantitatively similar manner to EN460 and with a slight better performance of I2. Next, we employed a non-reducing Immunoblotting to assess the ERO1A redox state in cells with compound exposure. At steady state, ERO1A in cells was mostly oxidized with high mobility on SDS-PAGE. Exposure to DTT (10 mM) reduced the disulfides in ERO1A and imparted a slow protein migration. In a similar manner, exposure of cells to either EN460, I2 or I3 resulted in the accumulation of an ERO1A form with lower mobility, suggesting ERO1A was trapped in its reduced state (Fig. 3C). None of the other compounds altered the redox state of ERO1A. To confirm the inhibitory effect of I2 and I3 on ERO1A in cells, we treated cells with different concentrations of the compounds and assessed ERO1A reduction in a dose-response experiment (Fig. 3D). I2, I3 and EN460 led to a marked ERO1A reduction at 20 μM. In contrast to I2, both I3 and EN460 were cytotoxic at 50 μM, as the low cell recovery suggested. Thus, these assays indicated I2 and I3, analogs of EN460, as two new top candidates for ERO1 inhibitors.

### Molecular docking of ERO1A inhibitors

**Fig. 4:**
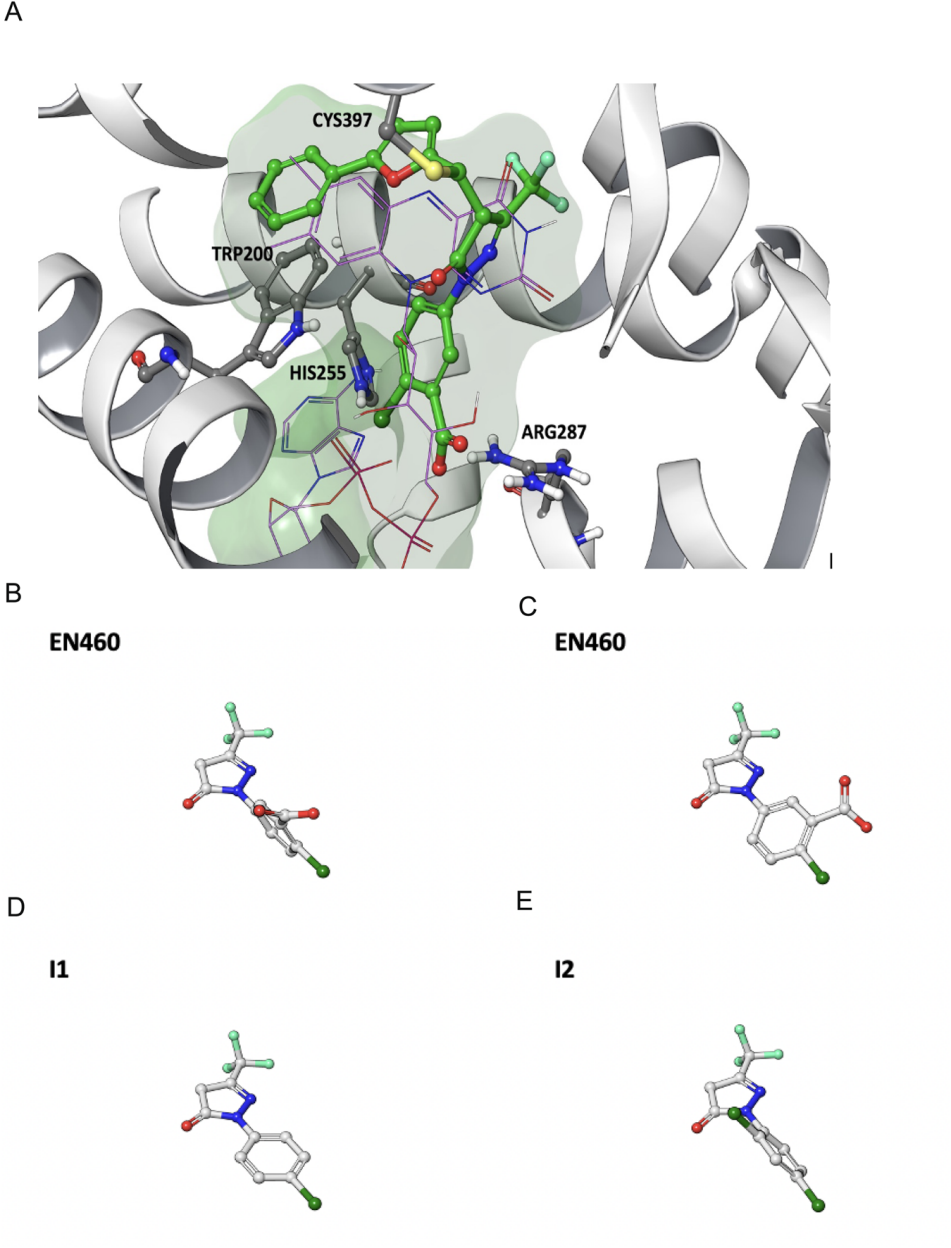
Docking analysis of ERO1A inhibition. **A** Predicted conformation of EN460 at the FAD binding pocket. EN460 (green carbon atoms) and the interacting amino acids (grey carbon atoms) are reported in a ball & stick representation. The crystallographic pose of FAD (thin magenta lines) is reported for comparison. The structure of ERO1 is reported in grey ribbons. The green mesh highlights the boundaries of the pocket. **B** Conformation of the substituted phenyl ring in the predicted bound conformation of EN460 **C**) in the energy minimum in water of EN460, **D**) in I1 and E) in I2.

Assuming the inhibition of ERO1A by EN460 occurs through a covalent interaction, we employed covalent ligand docking techniques to simulate the structure of the complex formed between the inhibitor and ERO1A. Our model positions EN460 within the pocket region originally occupied by the isoalloxazine moiety of FAD (Fig. 4A), consistent with experimental data indicating displacement of FAD upon EN460 binding [27]. Catalytic residue Cys397 initiates a nucleophilic attack at the carbon atom linking the pyrazolone and furan rings, elucidating the inactivity of I15 (in which the Michael acceptor is missing) and potentially explaining the inert nature of I14 due to distinct electronic properties of the substituent adjacent to the double bond. Additionally, a *π*-stacking interaction is established with the side chain of TRP200, while the chloro and carboxylate substituents interact with HIS255 and ARG287, respectively. The trifluoromethyl group occupies a sub-pocket proximal to the backbone of GLU186.

Our proposed model aligns with data from the preliminary structure-activity exploration, indicating how these interactions collectively contribute to inhibitory potency, possibly influencing the formation of a covalent bond. Derivatives unable to effectively engage TRP200 are inactive, due to insufficient length (e.g., I5, I7, I8, I9, I12) or substantial alteration in the substituent vector direction compared to the phenylfuran moiety of EN460 (e.g., I11, I13). The bulkier substituent in I10 cannot fit within the space occupied by EN460’s trifluoromethyl group. The interaction between the carboxylic group and ARG287 induces significant distortion in EN460’s geometry (Fig. 4B) compared to its energy-minimum conformation in water, where the pyrazolone ring and substituted phenyl ring are nearly coplanar (Fig. 4C). Except for I2, compounds unable to form a Coulombic interaction with ARG287’s side chain, such as I1, I4, and I6 maintain an almost coplanar arrangement between the two rings (as demonstrated by I1 in Fig. 4D) and are inactive. Owing to the *ortho* chloro substituent in that region, the energy-minimum conformation of I2 (Fig. 4E) closely mirrors the proposed enzyme-bound conformation of EN460. This implies that achieving inhibitory activity relies on adopting a non-coplanar rearrangement within the binding site and emphasizes the importance of the structural configuration over direct interaction with ARG287.

### Inhibitor-mediated change in the redox state of ERO1A

**Fig. 5:**
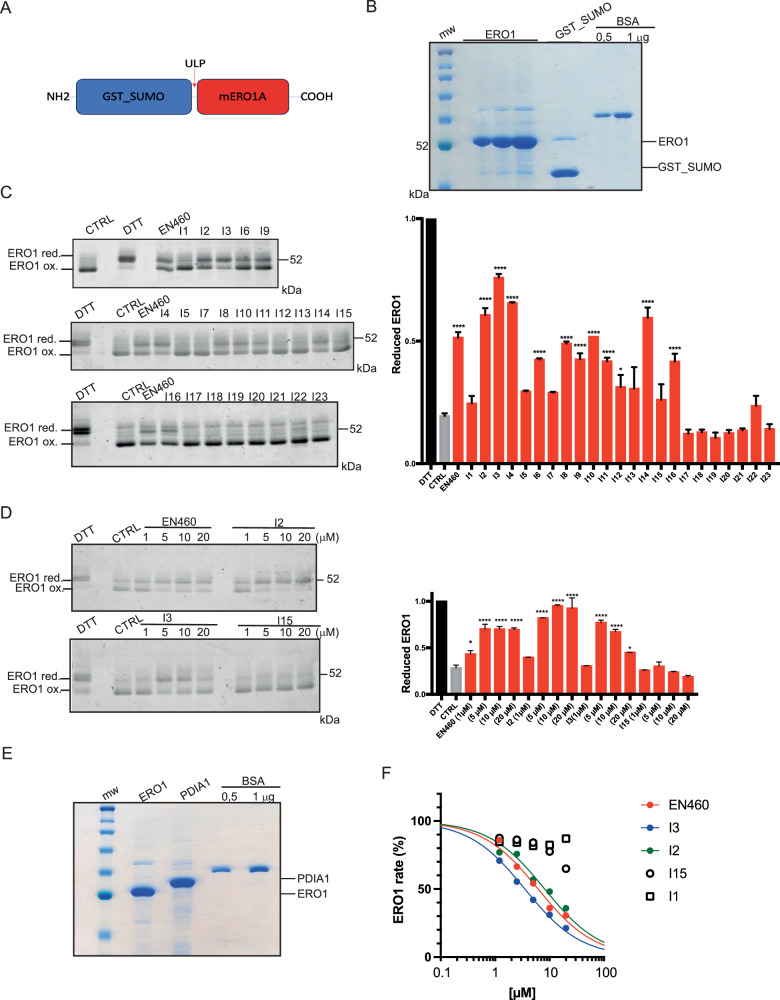
Inhibitor-mediated engagement and activity of ERO1A. **A** Schematic representation of the recombinant mouse ERO1A construct, indicating the ULP (a Sumo protease) cleavage site. **B** Coomassie-stained SDS-PAGE representing ERO1A cleaved from the upstream GST_SUMO, GST_SUMO, and BSA as a protein loading control. **C** Coomassie-stained nonreducing SDS-PAGE of ERO1A (1 μM) reacted with 20 μM of the 23 compounds. Representative experiments reproduced twice with different batches of ERO1A protein. On the right, bar graphs indicating the reduced ERO1A on total ERO1A (reduced ERO1A + oxidized ERO1A); the DTT-treated ERO1A was arbitrarily set to 1 (mean ± SEM, One-Way ANOVA). **D** Coomassie-stained non-reducing SDS-PAGE indicating ERO1A exposed to DTT or the concentrations indicated of EN460, I2, I3 and I15. Representative experiment reproduced twice. On the right, bar graphs indicating the reduced ERO1A on total ERO1A (mean ± SEM, One-Way ANOVA). **E** Coomassie-stained SDS-PAGE representing ERO1A, PDI1A1, and BSA as a protein loading control. **F** Concentration-dependent inhibition of the ERO1A-dependent AUR fluorescence in a kinetic assay employing ERO1A and PDIA1 (raw data in Fig Suppl. 2B). Curves were fitted using the equation: “log(inhibitor) vs. response - Variable slope” in Prism 10, to obtain the IC50 values with corresponding 95% interval of confidence, which were: 6.2 µM (5.1–7.5), 3.5 µM (2.9–4.3) and 8.1 µM (6.7–9.9) for EN640, I3 and I2, respectively.

To clarify whether active inhibitors bind directly to ERO1A and change its redox state, we purified recombinant mouse ERO1A (Fig. 5A and B). ERO1A recombinant protein (1μM) was exposed to EN460 and the twenty-three compounds (20 μM); after 30 min the reaction was quenched in NEM and loaded on a non-reducing SDS-PAGE stained with Coomassie. ERO1A recombinant protein (CTRL) was oxidized with high mobility on SDS-PAGE as the bulk ERO1A in cells [13, 40]. Exposure to DTT (10 mM) reduced the disulfides in ERO1A and imparted a slow protein migration. In a similar manner, exposure of the protein to EN460, I2, I3, I4, I6, I8, I9, 10, I11, I12, I14 and I16 resulted in the accumulation of an ERO1A form with lower mobility, suggesting the shift of ERO1A in its reduced state. None of the other compounds altered the redox state of ERO1A (Fig. 5C). The experiment was repeated with a different batch of ERO1A recombinant protein and ERO1A was exposed to I1, I2, I3, I6 and I15 confirming the ability of EN460, I2, I3 and I6 and the inability of I1 (planar conformation) and I15 (lack of Michael acceptor) to induce ERO1A redox shift (Sup. Figure 2A). To confirm the ERO1A engagement by I2 and I3 and compare it with EN460 and the inactive I15, a third batch of ERO1A was exposed to different concentrations of the compounds and ERO1A reduction was assessed in a dose-response experiment (Fig. 5D). I2 led to a slightly better ERO1A reduction than EN460 or I3, whereas I15, as expected, was inert. EN460, I2, I3, I1, and I15 were also analyzed for their ability to reduce the ERO1A-dependent AUR fluorescence in a kinetic assay employing ERO1A and PDIA1(Fig. 5E). Suppl Fig. 2B shows the raw data highlighting the concentration-dependent inhibition of the fluorescent signal. The initial rates of the reaction, measured in the absence or presence of different concentrations of the compounds, are reported in Fig. 5F and led to determining the IC50, i.e., the compound concentrations inhibiting 50% of the ERO1A reaction rate. The IC50 of EN460, I2, and I3 were quite similar and in the order of low micromolar (EN460 6.2μM; I3 3.5 μM and I2 8.1μM), while I15 and I1 were inactive (Fig. 5F, and Sup. Figure 2B). Taken together, these data confirm in vitro that the active ERO1A inhibitors, namely EN460, I2, and I3 bind ERO1, promoting changes in its redox state. In contrast, the inactive I1 and I15 did not change the ERO1 redox state and activity as they do in cells. However, other compounds such as I4, I6, I8, I9, 10, I11, I12, I14, and I16 that were inactive in cells in promoting ERO1 redox shift were instead active in vitro, suggesting low potency or limited uptake of these compounds in cells. Furthermore, EN460, I2, and I3 also efficiently inhibit ERO1A activity. In conclusion, the ERO1 redox state changed following the direct binding with its inhibitors, suggesting an inhibitor-mediated ERO1A redox shift in a reduced inactive state.

### Cytotoxicity and anti-angiogenic properties of ERO1A inhibitors in TNBC cells

**Fig. 6:**
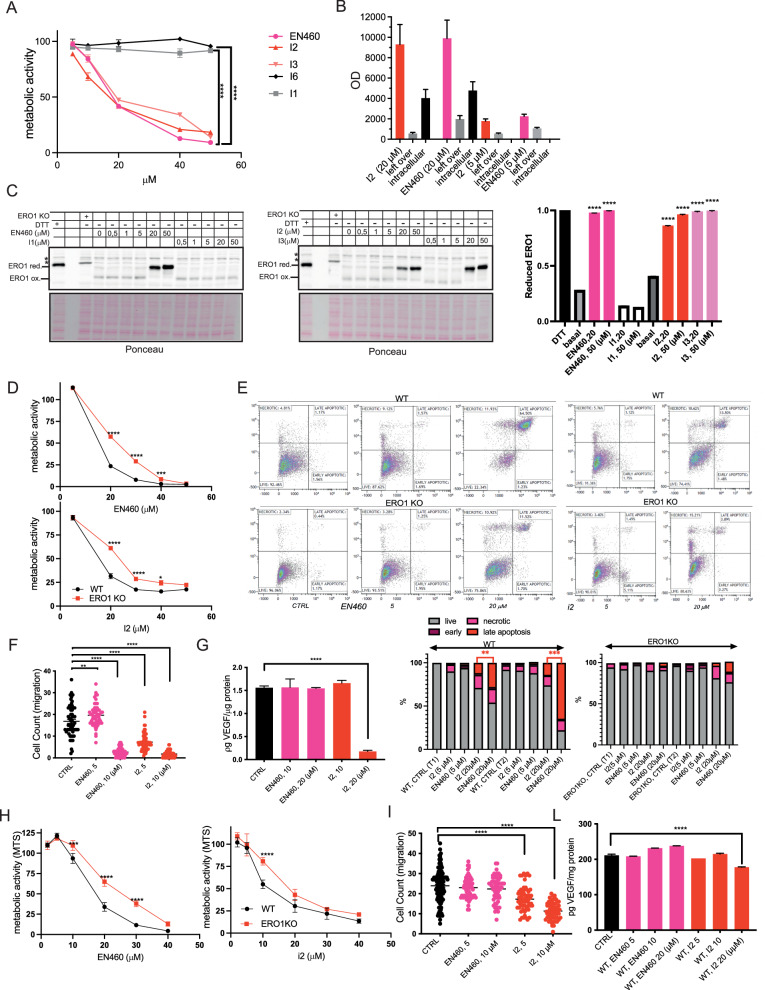
ERO1A inhibition triggers cytotoxicity and restrains VEGF in an ERO1A-dependent manner in TNBC cells. **A** MTS assay of MDAMB231 cells treated with different inhibitors (**I**). The value of the cells challenged with the vehicle alone was arbitrarily set to 100 and the value of the treated cells calculated as percentages (*N* = 5, mean ± SEM, Two-way ANOVA). **B** EN460 and I2 intracellular signal revealed by MALDI imaging in MDAMB231 cells from EN460 and I2-treated MDAMB231 cells, compared with the levels of the standard EN460 and I2. Left-over reports for the signal of the residual compound in the media, i.e., that was not taken up (*N* = 4). **C** Non-reducing Immunoblot of endogenous ERO1 in lysates of vehicle-treated MDAMB231 cells or exposed to DTT, EN460 and the different inhibitors (**I**). ERO1 red. stands for ERO1 reduced, ERO1 ox. for oxidized and * indicates a background band. Representative experiment reproduced twice. On the right, bar graph representing reduced levels of ERO1 on total ERO1 (oxidized + reduced), that was arbitrarily set to 1 for the DTT-treated cells (mean ± SEM, One-Way ANOVA). **D** MTS assay of MDAMB231 cells and ERO1A KO MDAMB231 cells treated with EN460 and I2 as indicated (*N* = 5, mean ± SEM, Two-way ANOVA). **E** Analysis of apoptotic cells. WT and ERO1 KO MDAMB231 were treated with EN460 or I2 for 6 (T1) and 14 hours (T2) (shown in the picture) and later analyzed for Annexin V and Propidium Iodide staining by flow cytometry. Based on the positivity to the stainings, cells were divided as live, necrotic, early and late apoptotic. Below, bar graph indicating the percentages of live, necrotic, early and late apoptotic and the significantly higher level of late apoptotic cells in EN460-treated cells (*N* = 3, *t*-test). **F** Dot plots representing the migrated MDAMB231 subjected to a Boyden Chamber motility assay (*N* = 3, mean ± SEM, One-way ANOVA). **G** Bar Graph representing VEGF secreted from MDAMB231cells by VEGF ELISA (*N* = 4, mean ± SEM, One-way ANOVA). **H** MTS assay of E0771 cells treated with EN460 and I2 (*N* = 5, mean ± SEM, Two-way ANOVA). **I** Dot plots representing migrated E0771 cells subjected to a Boyden chamber motility assay (*N* = 3, mean ± SEM, One-way ANOVA). **L** Bar graph representing VEGF secreted from E0771 cells by VEGF ELISA (*N* = 4, mean ± SEM, One-way ANOVA).

EN460, I2 and I3 inhibited MDAMB231 metabolic activity (MTS) in a dose-dependent manner while I1 and I6 were inert (Fig. 6A). To analyze the intracellular content of ERO1 inhibitors, we exploited MALDI imaging; the analysis indicated that EN460 and I2 were taken up by cells quantitively in a micromolar range (Fig. 6B). Analysis of ERO1A redox state in MDAMB231 cells, indicated that ERO1A was oxidized at steady-state and EN460, I2 and I3 trapped ERO1 in a reduced state whereas the inactive I1 had no effect on ERO1 redox state (Fig. 6C and Supplementary Figure 3A); this suggests a direct relationship between the impaired cell metabolic activity and ERO1 inhibition.

As I3 is more cyto-toxic (similarly to EN460) than I2 in MDAMB231 cells, we conducted further studies, only analyzing I2 and EN460 side-by-side. To assess whether I2 and EN460-reduced cell metabolic activity was dependent on ERO1A expression, we analyzed the response to EN460 and I2 of both WT and ERO1 KO MDAMB231 cells. ERO1 KO cells were more resistant to EN460 and I2, suggesting that EN460 and I2-reduced cell metabolic activity depends on ERO1 (Fig. 6D). At parity of dosage, EN460 induced more apoptosis than I2, as indicated by the staining of Anexin V and propidium iodide-positive cells. Furthermore, there were late apoptotic cells among EN460-treated ERO1 KO cells (Fig. 6E), and more than among the I2-treated counterparts, possibly suggesting a more promiscuous ERO1-independent effect of EN460.

To rule out any EN460 and I2-dependent toxicity due to effects on cellular glutathione (GSH) levels, the more abundant thiol-containing molecule in cells [41], we measured cellular GSH after cellular treatment with EN460 and I2, and we did not detect any relevant effect of ERO1A inhibitors on GSH levels (Supplementary Figure 3B). Despite the lower cytotoxicity of I2, I2 impaired cellular migration and VEGF secretion more efficiently than EN460 (Figs. 6F and 6G). To understand whether the effects of EN460 and I2 were reproducible on a second breast cancer cell line, we employed E0771, a murine breast cancer cell line. We analyzed the effects of EN460 and I2 on the metabolic activity of E0771 murine breast cancer and compared the effects of WT and ERO1 KO cells to clarify whether the effects of the two inhibitors depended on ERO1 levels (Figure Supplementary 3C and Fig. 6H). A side-by-side comparison of EN460 and I2 potency indicated that the two were similar in terms of impaired E0771 metabolic activity, while ERO1 KO cells were more resistant to the two inhibitors (Fig. 6H). As expected, the EN460 and I2-dependent E0771 metabolic impairment correlated with a redox reduction of ERO1 (Figure supplementary 3D). EN460-treated cells were slightly more apoptotic than I2-treated counterparts while I1-treated cells were comparable to the controls (Figure supplementary 3E). As in MDAMB231 cells, I2-treated E0771 cells were more impaired in migration and VEGF secretion than EN460-treated counterparts (Fig. 6I and L).

These observations suggest that I2 exerts its anti-cancer efficacy by impairing VEGF and related cell migration.

### Efficacy of ERO1A inhibition in TNBC-bearing mice

**Fig. 7:**
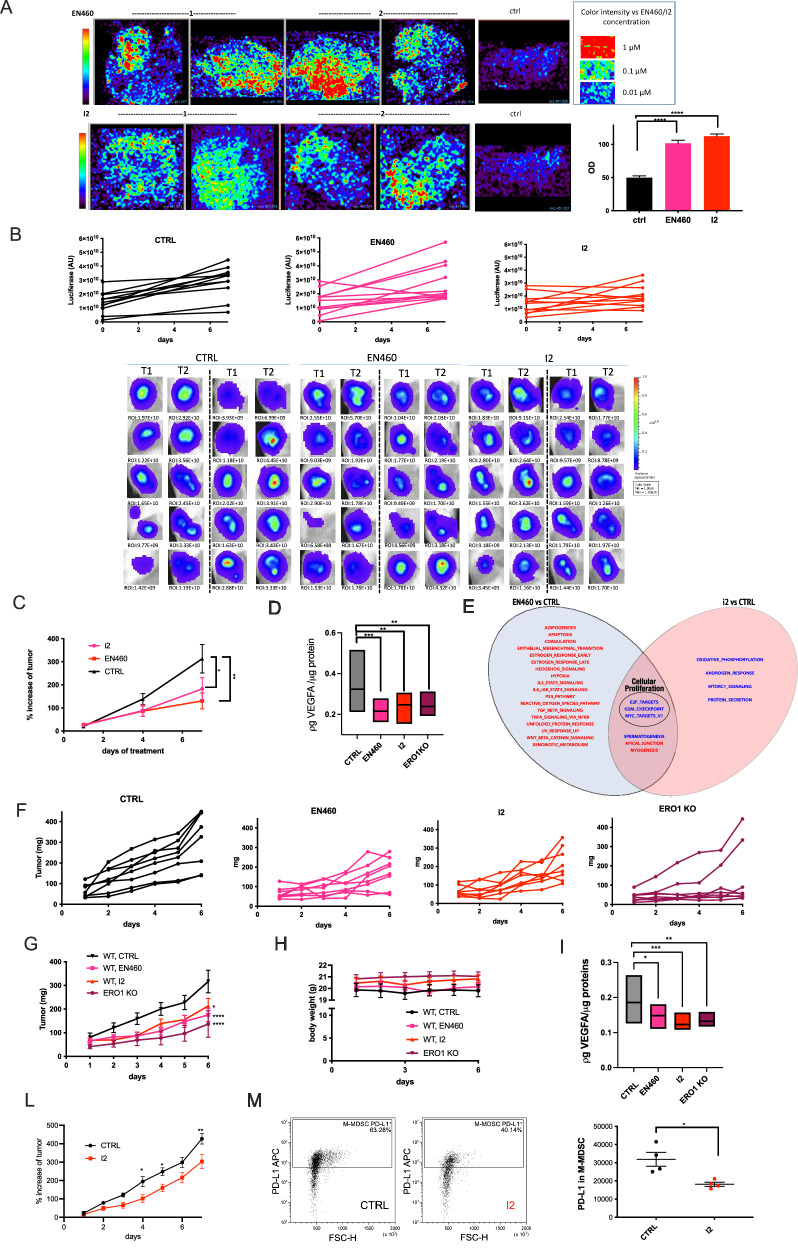
Anticancer effects of ERO1A inhibition in TNBC syngeneic and xenograft-bearing mice. **A** EN460 and I2 signal revealed by MALDI imaging in representative primary MDAMB231 breast tumor sections from CTRL or EN460 and I2-treated MDAMB231 tumor-bearing SCID mice. On the right, signal intensity in Optical density (OD) (*N* = 4, mean ± SEM, One-way ANOVA). **B** Bioluminescence signals of primary breast tumors from SCID mice which were injected subcutaneously with MDAMB231 cells. Mice-bearing tumors were treated six times with EN460, I2, or vehicle, and the luminescence in AU was detected at t = 0 (T1) and t = 7 days (T2). Below are the bioluminescence signals of tumors. **C** The graph shows the percentage in tumor growth compared to day 0 when mice were randomized before the treatment (*N* = 10, mean ± SEM, Two-way ANOVA). **D** Graph representing VEGFA levels in primary breast tumors (One-way ANOVA). **E** Venn diagram showing the results of a differential transcriptomic analysis, comparing two sets of sample treatments: EN460 vs. CTRL-treated and I2 vs. CTRL-treated tumors (*N* = 5). Each circle contains the upregulated and downregulated pathways identified in the respective comparisons. The pathways are taken from the Hallmark gene sets collection (MSigDB). The analysis focused on significantly regulated pathways (False Discovery Rate, FDR < 0.05). The intersection of the two sets reveals a common downregulation of pathways related to cellular proliferation. **F** The graph shows tumor growth caliper measurements of each mouse. **G** The graph shows tumor growth caliper measurements of the mean of the treated mice (*N* = 8, mean ± SEM, Two-way ANOVA). **H** Mouse weights during treatment. **I** Graph representing VEGFA levels in primary breast tumors (One-way ANOVA). **L** The graph shows the percentage of orthotopic E0071 breast tumor growth compared to day 0 when mice were randomized before treatment (*N* = 8, mean ± SEM, Two-way ANOVA). **M** Orthotopic E0071 breast tumors. Representative cytofluorimeter plots of a PD-L1^+^ subset of monocytic myeloid-derived suppressor cells (M-MDSC) and dot plots of quantification of PD-L1 expression (MFI) in M-MDSC (*N* = 4, unpaired *t*-test).

We examined the in vivo activities of EN460 and I2 in TNBC-bearing mice in the models of human MDAMB231 cells injected in SCID mice and murine E0771 injected in syngeneic C57BL/6 J mice. Mice tolerated the compounds well up to the highest administrable dose in terms of solubility, yielding 10 mg/kg EN460 or I2 injected intraperitoneally (i.p.), without any overt signs of toxicity in a week of treatment. Luciferase-expressing MDAMB231 tumor-bearing SCID mice were treated once a day, with 10 mg/kg i.p of EN460 or I2 for six days (together with vehicle-treated mice). We longitudinally followed the tumor growth by in vivo bioluminescence imaging and caliper measurements. The EN460 and I2 treatments resulted in appreciable levels of the two ERO1 inhibitors in the tumors, as proved by MALDI imaging analysis (Fig. 7A), suggesting that the two compounds efficiently reached the tumor. Luminescence was lower in I2-treated bearing tumor mice, but the differences in the measurements among mice precluded any statistical significance (Fig. 7B). However, there was a slight decrease in tumor growth percentage with EN460 and I2 compared to vehicle-treated controls but tumors from EN460 and I2-treated MDAMB231-bearing mice did not significantly differ (Fig. 7C).

Then, since ERO1A enhances VEGFA expression [24] and ERO1A inhibitors reduce VEGFA in cells, we examined VEGFA levels in primary tumors using ELISA. VEGFA levels decreased in EN460 and I2-treated MDAMB231-bearing mice and in ERO1 KO MDAMB231-bearing mice (Fig. 7D).

To clarify the pathways involved in the tumor response to ERO1 inhibition, we sequenced the total mRNA of primary tumors from EN460- and I2-treated MDAMB231 tumor-bearing mice and compared them to control tumors, i.e., from vehicle-treated MDAMB231 tumor-bearing mice. Pathway analysis in tumors subjected to the two treatments suggested the downregulation of gene sets involved in cellular proliferation represented by E2F targets, G2M checkpoints, and MYC targets, whose high score is typically associated with a proliferative cancer phenotype and aggressiveness [42]. Notably, the tumors treated with the two compounds also shared the downregulated pathways involved in cellular proliferation (Fig. 7E).

This indicates that ERO1 inhibition triggers a defect in cellular proliferation consistently with tumor-reduced growth.

The role of ERO1 was also analyzed in syngeneic breast tumor-bearing mice. E0771 murine breast cancer cells were transplanted subcutaneously in syngeneic C57BL/6 J mice. When tumors were palpable, the treatments with EN460 and I2 started and continued for six days. EN460 and I2 both impaired tumor growth in this model, like tumors from ERO1 KO E0771-bearing mice (Fig. 7F and G). Tolerance of the compounds was good, with no overt signs of toxicity or weight loss during the treatment (Fig. 7H). As in MDAMB231 tumors, VEGFA levels decreased in both cohorts of tumors from EN460 and I2-treated E0771 tumor-bearing mice and from E0771 ERO1 KO tumor -bearing mice.

Finally, given the evidence of the involvement of ERO1A in the tumor microenvironment [43], we analyzed whether ERO1A inhibition impinges on the breast cancer tumor microenvironment. For this purpose, we orthotopically injected E0771 in C57/BL6J mice. When the tumors were palpable, mice were randomized to receive vehicle or I2 and the treatments continued for six days. Caliper measurements, used to analyze the tumor growth, showed a slight reduction in tumors from I2-treated mice. At the end of the treatment, the tumors were analyzed for the intratumoral myeloid and lymphoid populations by flow cytometry, mainly focusing on PD-1/PD-L1 immune checkpoint axis [44]. While PD-1 expression in T cells (CD4+ and CD8 + ) resulted unchanged (Supplementary Figure 3), I2-treated tumors revealed a reduced expression of PD-L1 in the monocytic myeloid-derived suppressor cell (M-MDSC) subset monocytes and in tumor-associated macrophages (TAM) (Fig. 7M and Figure Supplementary 4).

These findings suggest that ERO1A inhibition triggers a scant decrease in tumor mass, a significant defect in cellular proliferation pathways, in the master regulator of angiogenesis VEGFA, and the immune checkpoint PD-L1. Thus, these findings imply that ERO1A inhibition might limit tumor fitness by impairing tumor angiogenesis and improving anti-tumor immunity by limiting VEGF and immune checkpoints.

## Discussion

Although ERO1A is the predominant protein disulfide oxidase in cells [13], its activity is dispensable in healthy cells [10]. On the other hand, ERO1A is upregulated in many tumors [15, 45], driving immunosuppression and promoting poor clinical outcomes [23, 43]. It is also upregulated in hypoxia [5], a hallmark of malignant breast cancer, enhancing tumor aggressiveness [25, 46]. Here, immunofluorescence on human breast tumor samples indicate that ERO1A is more upregulated in aggressive and highly proliferative TNBC than in other breast tumor types, suggesting a critical role of ERO1A in this still incurable breast tumor. The expression of ERO1A significantly correlates with that of VEGF and other HIF1-dependent angiogenic factors, suggesting a close association between ERO1A, multiple angiogenic regulators, and angiogenesis in this tumor type [5, 23]. Our previous studies indicated that ERO1A genetic deletion in TNBC murine models impaired VEGF folding under hypoxic conditions, affecting cancer fitness in terms of angiogenesis and dissemination, and potentiated the otherwise scant effects of antiangiogenic therapy solely targeting VEGFA [16, 19, 23].

These results are consistent with the efficacy of ERO1A genetic deletion in TNBC models and its importance for cancer cell survival and related angiogenesis. Furthermore, the dispensability of ERO1A in healthy cells and the lack of an overt phenotype of ERO1A-devoid murine models, in stark contrast with the relevance of ERO1A in TNBC angiogenesis, make ERO1A an ideal target for TNBC therapy so an ERO1A inhibitor might be a promising cancer drug with a unique broad antiangiogenic feature.

EN460 is the first ERO1A inhibitor that, together with the structurally similar QM295, came out from a high-throughput screening [27]. An adduct formation between ERO1A and EN460 undermines the FAD prosthetic group. This information inferred EN460 binding to other FAD-containing enzymes, including MAO-A and MAO-B, which might be potential off-targets [47]. Recently, B12–5 was identified as a new ERO1 inhibitor that, despite the different chemistry, shares with EN460 the displacement of the FAD prosthetic group in ERO1 and the poor water solubility [31]. Given the high degree of sequence identity in the region surrounding ERO1A and ERO1B’s FAD binding pocket, EN460 and B12-5 are likely to inhibit the activity of both ERO1 paralogues.

EN460 inhibits ERO1A in a low micromolar range by exploiting a potent Michael acceptor electrophile. Because of this property, EN460 can potentially form covalent bonds with abundant thiol-containing compounds in cells, such as glutathione, raising concerns about the possibility of lack of selectivity and off-target effects. Thus, although EN460 efficiently inhibits ERO1A in vivo, trapping it in an inactive reduced state [27], its potential lack of selectivity precluded its clinical use. Indeed, compounds in the class of covalent inhibitors containing an electrophile, such as a Michael acceptor, and targeting a cysteine have been overlooked in clinical use. However, current interest has been aroused by the recent patents and clinical success of targeted covalent inhibitors in cancer therapy [48, 49]. Pivotal examples in this direction are the Michael acceptor-containing inhibitors of EGFR, which react with cysteine residue (Cys797) [50] and neratinib, which potently inhibits HER2 by covalently binding Cys805, approved by the FDA for treatment of HER2^+^ breast cancer in 2017 [51].

We, therefore, organized a fine-tuned structure-activity analysis and lead compound optimization of EN460 to increase its potency and the lack of solubility in aqueous solution aimed at speed up the preclinical validation of ERO1 inhibitors as new anti-cancer drugs.

This led to identifying the pharmacophoric groups of EN460 that are instrumental in promoting ERO1A inhibition and improving its potency by testing the compounds in different settings, in vitro, in vivo, and in breast cancer models. Cell-based screening was the initial crucial step of our selection process for the compounds, consisting of evaluating VEGFA secretion and ERO1A redox state in vivo. This in vivo screening, although more time- and labor-consuming than in vitro, offers the advantage of highlighting issues related to limited solubility or toxicity of new compounds; this means that compounds that lack any in vivo applicability or have a broad, toxic, non-ERO1-based effect can be immediately discarded. Importantly, this approach led to the selection of effective compounds, the hits I2 and I3, derivatives of EN460 phenyl group, which inhibit the secretion of ERO1 target’s VEGFA while trapping ERO1A in a reduced inactive state.

In vitro assays confirmed the direct binding of the active EN460 analogs, I2 and I3 to ERO1A recombinant protein and their ability to induce a redox shift in the protein and to inhibit the enzyme despite the inert nature of the inactive compounds such as I1, which has a co-planar structure or I15 which lacks the Michael acceptor. These findings suggest that the 3-carboxylic acid in EN460, bonded to the phenyl group, is permissive to chemical variants, and the lack of the carboxyl group (I2) does not impair ERO1A inhibition, as previously reported [47]. Docking studies pointed to a covalent binding between the catalytic Cy397 of ERO1A and EN460 or B12-5 [31], to inhibit ERO1A. However, docking studies also illustrate the importance of non-bonded interactions between the amino acids Trp200, His255, and Arg287 of ERO1A and EN460. Interestingly, a retrospective docking study based on the ability of I2 and I3 to inhibit ERO1A suggested that ERO1A inhibition entails a non-coplanar rearrangement of the compound within the binding site, thus stressing the importance of the compound’s structural configuration and explaining why lack of the carboxyl group in I2 does not compromise ERO1 inhibition, while I1 is inactive. The side-by-side comparison of EN460 and I2 in TNBC cells revealed a dose-effect and ERO1A-dependent reduced cell viability.

Compared with EN460, I2 was more efficient in restraining VEGF secretion and the related cellular migration of TNBC cells. I2 treatments of TNBC-bearing mice efficiently reached the tumor and impaired its fitness by reducing VEGF and the immune check point PD-L1, with no apparent toxicity in mice. In support of the lack of toxicity, we did not detect gross changes in glutathione levels in vivo, although in vitro these compounds bind glutathione and other thiol-containing compounds [27].

In vitro assays suggest that IC50 of ERO1A inhibitors is in the micromolar range as the inhibitory concentration in vivo, potentially ruling out their promiscuous binding in vivo [31]. Furthermore, to our knowledge, other compounds with a chemistry of potent electrophile and targeting a redox amino acid, the selenocysteine, of another redox enzyme, Thioredoxin reductase 1, work in a low micromolar range [48] in vivo. This could suggest the need to use concentrations in the micromolar range to target redox amino acids in redox enzymes.

To conclude, our findings prove the feasibility and the efficacy of ERO1A inhibition through small molecules in TNBC by targeting a catalytic active cysteine. They might also be of interest for clinical application in diseases other than cancer, where an excess of ERO1A activity might be detrimental [31, 52, 53].

## Conclusion

ERO1A is highly expressed in aggressive and poorly responsive to treatments TNBC. The dispensability of ERO1A in healthy cells, in stark contrast with the relevance of ERO1A in TNBC, makes ERO1A an ideal target for TNBC therapy.

ERO1A inhibitors impair the angiogenic VEGF and migration of TNBC cells. Furthermore, they restrain aggressive TNBC in vivo, reducing VEGF and the immune checkpoint PD-L1 in the tumor microenvironment. Thus, ERO1A inhibition with small molecules is an innovative and feasible approach for TNBC therapy (Graphical Abstract).

## Supplementary information

Supplementary material

## Data Availability

The datasets generated during the current study are available from the corresponding author on reasonable request. The RNA sequencing data have been deposited at EMBL-EBI *Annotare database* (https://www.ebi.ac.uk/fg/annotare/) under the accession numbers: E-MTAB-14269.
