# Supplementary material for: Small molecule-mediated inhibition of the oxidoreductase ERO1A restrains aggressive breast cancer by impairing VEGF and PD-L1 in the tumor microenvironment
Source: Cell Death Dis. 2025 Feb 17;16(1):105. doi: 10.1038/s41419-025-07426-1 (PMC11833095; doi:10.1038/s41419-025-07426-1)

Supplementary Table of Contents

1. **General information** **S2**
2. **General procedure for the preparations of EN460 and inhibitors I_{1-5}, I_{7-14}** **S3**
3. **Characterization data of EN460 and inhibitors I_{1-5}, I_{7-14}** **S4**
4. **Synthetic procedure and characterization data for I_6** **S17**
5. **Synthetic procedure and characterization data for I_{15}** **S18**
6. **Synthetic procedure and characterization data for I_{16}** **S19**
7. **References** **S20**
8. **Copies of ^{1}H NMR and ^{13}C NMR spectra** **S21**
9. **Supplementary Figure Legends and Figures**
10. **Uncropped Immunoblots and Coomassie stained SDS-PAGEs**

Experimental Section

1. General informations

All reactions were conducted in air unless otherwise noted. Column chromatography purifications were performed in flash chromatography conditions using Merck 230-400 mesh silica gel. Analytical thin layer chromatography (TLC) was carried out on Merck silica gel plates (Silica Gel 60 F254), that were visualized by exposure to ultraviolet light and an aqueous solution of KMnO4. ^{1}H NMR and ^{13}C NMR spectra were recorded on a Bruker Avance 400 spectrometer, using DMSO-d_6 or $CDCl_3$ as solvent. ^{1}H NMR chemical shifts (δ scale) are reported in parts per million (ppm) relative to the central peak of the solvent, DMSO-d_6 (δ = 2.50 ppm), $CDCl_3$ (δ = 7.26 ppm). Data are reported as follows: chemical shift, multiplicity (s = singlet, brs = broad singlet, d = doublet, t = triplet, m = multiplet), coupling constants (Hz) and integration. ^{13}C NMR chemical shifts are reported in ppm (δ) relative to DMSO-d_6 (δ = 39.52 ppm), $CDCl_3$ (δ = 77.16 ppm). Yields refer to isolated material. All the synthesized inhibitors tested (**EN460**, **I_1-I_{16}**) had purity greater than 95%, judged by ^{1}H NMR spectroscopy and high-pressure liquid chromatography. HPLC analyses were performed on a Waters HPLC/UV/MS system (separation module Alliance HT2795, photo diode array detector 2996, mass detector Micromass ZQ, using column Phenomenex C6-phenyl 150 mm × 4.60 mm × 5 mm. The mobile phase consisted of acetonitrile and water (e containing 0.1% formic acid). A linear gradient of 70% to 100% acetonitrile over 8 minutes was used with a 10-minute run time at a flow rate of 1 mL/min. High-resolution mass spectrometry (HRMS) analysis was performed using a Q-TOF micro TM mass spectrometer. Starting materials: 4,4,4-trifluoroacetoacetate, methylacetoacetate and 3-oxo-3-phenylpropanoate were purchased from the best-known commercial suppliers and used without further purification. All the aromatic hydrazines hydrochloride were purchased from the best-known commercial suppliers and used without further purification. 5-phenylfuran-2-carbaldehyde was synthesized according to the literature procedure.[1] All the other aromatic aldehydes were purchased from the best-known commercial suppliers and used without further purification. All other chemicals and solvents were purchased from Merck Sigma-Aldrich and used without further purification. **I_{16}** was synthesized according to the literature procedure. [2]

Compounds **I_{17-23}** are commercially available, were purchased from Enamine (Enamine Ltd., Kiev) and had a declared purity of 90%.

2. **General procedure for the preparations of EN460, inhibitors I1-5, I7-14.**

EN460 and inhibitors **I$_{1-5}$**, **I$_{7-14}$** were prepared according to the literature procedure described by L. A. Hazlehurst [1] and reported in **Scheme 1.**

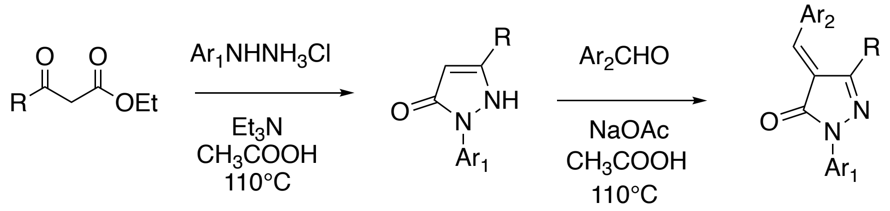

Scheme 1

To a solution of appropriate Hydrazine hydrochloride (1 equiv.) and appropriate acetoacetate (1 equiv.), in acetic acid (1.0 M), was added triethylamine (1 equiv.). The resulting mixture was heated at reflux for 24h. The excess of acetic acid was evaporated under reduced pressure, resulting in viscous oil. The crude product was partitioned between water and dichloromethane, the phases were separated, and the organic layer was dried with sodium sulfate and evaporated under reduced pressure to afford the corresponding pyrazolone that was used in the next step without further purification.

To a solution of the above obtained pyrazolone derivatives (1 equiv.) and the appropriate aldehyde (1 equiv.), in acetic acid (0.5 M), was added a catalytic amount of sodium acetate (0.1 equiv). The resulting mixture was heated to reflux for 4 h and the excess of acetic acid was evaporated under reduced pressure and then co-evaporated with toluene (5 mL x3). The crude mixture was purified by crystallization with a mixture of water-acetone (5:1). The precipitate formed was collected by filtration, washed three times with water and dried.

3. Characterization data of EN460 and inhibitors I_{1-5}, I_{7-14}.

(E)-5-(4-((5-phenylfuran-2-yl)methylene)-5-oxo-3-(trifluoromethyl)-4,5-dihydro-1H-pyrazol-1-yl)-2-chlorobenzoic acid EN460

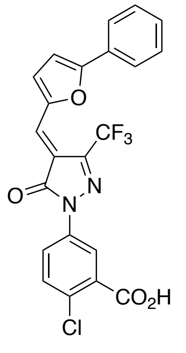

EN460

The reactions were carried out following the general procedure using *2-chloro-5-hydrazinobenzoic acid hydrochloride* (223.5 mg, 1 mmol) and 4,4,4-trifluoroacetoacetate (0.146 mL, 1 mmol, 1 equiv.) for the preparation of pyrazolone core, and using *5-phenylfuran-2-carbaldehyde* (172.18 mg, 1 mmol, 1 equiv.) for the last step of inhibitor synthesis.
The crude mixture was purified by crystallization with a mixture of water-acetone (5:1) and the precipitate formed was collected by filtration, washed three times with water and dried. The title compound **EN460** was isolated as a red solid with 80% yield over two steps (368 mg, 0.8 mmol).
^{1}H NMR (400 MHz, DMSO-d_6) δ 11.79 (brs, 1H), 9.04 (brs, 1H), 8.625 (d, J = 4 Hz, 1H), 8.215 (dd, J = 4 Hz, 8 Hz, 1H), 8.11-8.08 (m, 2H), 7.89 (s, 1H), 7.70 (d, J = 8 Hz, 1H), 7.62-7.53 (m, 4H).
^{13}C NMR (100 MHz, DMSO-d_6) δ 166.55, 163.16, 161.44, 150.51, 140.30, 140.05, 136.77, 132.14, 132.03, 131.54, 130.54, 129.85, 128.88, 128.35, 126.39, 125.48, 123.31, 121.54, 113.46, 113.39.
LC-MS retention time 5.47 min; LRMS m/z $[M+H]^+$: 461.3.

HRMS (ESI-TOF) m/z calcd. for $C_{22}H_{13}ClF_3N_2O_4$ $[M+H]^+$: 461.0510; found 461.0519.
Mp: 204 °C–205°C

(E)-2-(4-chlorophenyl)-4-((5-phenylfuran-2-yl) methylene)-5-(trifluoromethyl)-2,4-dihydro-3H-pyrazol-3-one I_1

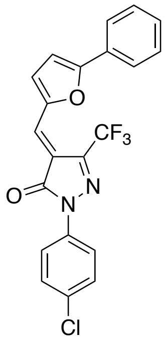

I1

The reactions were carried out following the general procedure using (4-chlorophenyl)hydrazine *hydrochloride* (179.04 mg, 1 mmol) and 4,4,4-trifluoroacetoacetate (0.146 mL, 1 mmol, 1 equiv.) for the preparation of pyrazolone core, and using *5-phenylfuran-2-carbaldehyde* (172.18 mg, 1 mmol, 1 equiv.) for the last step of inhibitor synthesis.

The crude mixture was purified by crystallization with a mixture of water-acetone (5:1) and the precipitate formed was collected by filtration, washed three times with water and dried. The title compound **I_1** was isolated as a red solid with 60% yield over two steps (250 mg, 0.6 mmol).

^{1}H NMR (400 MHz, DMSO-d_6) δ 8.81 (brs, 1H), 8.11-8.08 (m, 2H), 7.95-7.91 (m, 2H), 7.82 (s, 1H), 7.655 (d, J = 4 Hz, 1H), 7.61-7.54 (m, 5H).

^{13}C NMR (100 MHz, DMSO-d_6) δ 163.00, 150.53, 136.83, 131.60, 131.52, 130.47, 129.88, 129.58, 129.15, 126.36, 125.49, 122.66, 121.67, 113.63, 113.28, 106.12, 102.03.

LC-MS retention time 6.77 min; LRMS m/z $[M+H]^+$: 417.3.

HRMS (ESI-TOF) m/z calcd. for $C_{21}H_{13}ClF_3N_2O_2$ $[M+H]^+$: 417.0612; found 417.0604.

Mp: 230°C–231°C.

(E)-2-(2,4-dichlorophenyl)-4-((5-phenylfuran-2-yl)methylene)-5 (trifluoromethyl)-2,4-dihydro-3H-pyrazol-3-one I_2

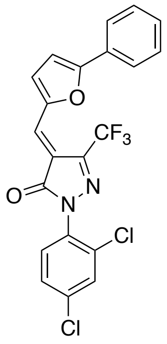

I2

The reactions were carried out following the general procedure using 2,4-dichlorophenyl)hydrazine *hydrochloride* (213.48 mg, 1 mmol) and 4,4,4-trifluoroacetoacetate (0.146 mL, 1 mmol, 1 equiv.) for the preparation of pyrazolone core, and using *5-phenylfuran-2-carbaldehyde* (172.18 mg, 1 mmol, 1 equiv.) for the last step of inhibitor synthesis.

The crude mixture was purified by crystallization with a mixture of water-acetone (5:1) and the precipitate formed was collected by filtration, washed three times with water and dried.. The title compound **I$_2$** was isolated as a red solid with 55% yield over two steps (248 mg, 0.55 mmol).

^{1}H NMR (400 MHz, DMSO-d$_6$) δ 8.70 (brs, 1H), 8.1-8.08 (m, 2H), 7.93 (d, J = 4 Hz, 1H), 7.85 (s, 1H), 7.71-7.73 (m, 1H), 7.66-7.63 (m, 2H), 7.60-7.53 (m, 3H).

^{13}C NMR (100 MHz, DMSO-d$_6$) δ 162.91, 150.50, 140.12, 139.76, 135.28, 133.62, 133.00, 131.88, 131.44, 130.32, 129.81, 128.92, 128.40, 126.31, 126.15, 121.67, 118.97, 113.14, 112.25.

LC-MS retention time 6.05 min; LRMS m/z $[M+H]^+$: 451.3.

HRMS (ESI-TOF) m/z calcd. for $C_{21}H_{12}Cl_2F_3N_2O_2$ $[M+H]^+$: 451.0222; found 451.0231.

Mp: 186°C–187°C

(E)-3-(5-oxo-4-((5-phenylfuran-2-yl)methylene)-3-(trifluoromethyl)-4,5-dihydro-1H-pyrazol-1-yl)benzoic acid I$_3$

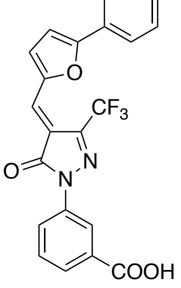

I3

The reactions were carried out following the general procedure using 3-hydrazineylbenzoic acid *hydrochloride* (188.6 mg, 1 mmol) and 4,4,4-trifluoroacetoacetate (0.146 mL, 1 mmol, 1 equiv.) for the preparation of pyrazolone core, and using *5-phenylfuran-2-carbaldehyde* (172.18 mg, 1 mmol, 1 equiv.) for the last step of inhibitor synthesis.

The crude mixture was purified by crystallization with a mixture of water-acetone (5:1) and the precipitate formed was collected by filtration, washed three times with water and dried. The title compound **I$_3$** was isolated as a red solid with 40% yield over two steps (171 mg, 0.4 mmol).

^{1}H NMR (400 MHz, DMSO-d$_6$) δ 13.20 (brs, 1H), 8.85 (brs, 1H), 8.48-8.46 (m, 1H), 8.15-8.13 (m, 1H), 8.08 (d, J = 4 Hz, 2H), 7.89-7.87 (m, 1H), 7.82 (s, 1H), 7.67-7.61 (m, 2H), 7.61-7.53 (m, 3H).

^{13}C NMR (100 MHz, DMSO-d$_6$) δ 167.24, 162.96, 150.50, 139.68, 138.17, 132.39, 132.14, 131.45, 130.35, 129.97, 129.91, 129.82, 128.38, 127.06, 126.33, 125.47, 123.88, 120.40, 113.69, 113.28.

LC-MS retention time 4.42 min; LRMS m/z [M+H]$^+$: 427.4.

HRMS (ESI-TOF) m/z calcd. for $C_{22}H_{14}F_3N_2O_4$ [M+H]$^+$: 427.0900; found 427.0893.

Mp: 195°C–196°C

(E)-4-(5-oxo-4-((5-phenylfuran-2-yl)methylene)-3-(trifluoromethyl)-4,5-dihydro-1H-pyrazol-1-yl)benzoic acid I$_4$

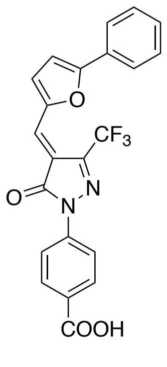

I4

The reactions were carried out following the general procedure using 4-hydrazineylbenzoic acid *hydrochloride* (188.6 mg, 1 mmol) and 4,4,4-trifluoroacetoacetate (0.146 mL, 1 mmol, 1 equiv.) for the preparation of pyrazolone core, and using *5-phenylfuran-2-carbaldehyde* (172.18 mg, 1 mmol, 1 equiv.) for the last step of inhibitor synthesis.

The crude mixture was purified by crystallization with a mixture of water-acetone (5:1) and the precipitate formed was collected by filtration, washed three times with water and dried. The title compound **I$_4$** was isolated as a red solid with 50% yield over two steps (213 mg, 0.5 mmol).

^{1}H NMR (400 MHz, DMSO-d_6) δ 13.00 (s, 1H), 8.82 (brs, 1H), 8.09-8.06 (m, 6H), 7.81 (brs, 1H), 7.655 (d, J = 4 Hz, 1H), 7.60-7.55 (m, 3H).

^{13}C NMR (100 MHz, DMSO-d_6) δ 178.39, 167.30, 159.21, 158.63, 152.21, 151.66, 150.51, 143.14, 131.21, 130.80, 130.15, 129.95, 129.73, 126.33, 125.40, 120.37, 118.99, 109.31, 100.10.

LC-MS retention time 4.40 min; LRMS m/z $[M+H]^+$: 427.3.

HRMS (ESI-TOF) m/z calcd. for $C_{22}H_{14}F_3N_2O_4$ $[M+H]^+$: 427.0900; found 427.0907.

Mp: 300°C–301°C

(E)-5-(4-benzylidene-5-oxo-3-(trifluoromethyl)-4,5-dihydro-1H-pyrazol-1-yl)-2-chlorobenzoic acid I_5

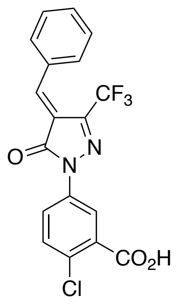

I5

The reactions were carried out following the general procedure using *2-chloro-5-hydrazinobenzoic acid hydrochloride* (223.5 mg, 1 mmol) and 4,4,4-trifluoroacetoacetate (0.146 mL, 1 mmol, 1 equiv.) for the preparation of pyrazolone core, and using *benzaldheyde* (106.12mg, 0.146 mL,1 mmol, 1 equiv.) for the last step of inhibitor synthesis.

The crude mixture was purified by crystallization with a mixture of water-acetone (5:1) and the precipitate formed was collected by filtration, washed three times with water and dried. The title compound **I_5** was isolated as a red solid with 45% yield over two steps (177.6 mg, 0.45 mmol).

^{1}H NMR (400 MHz, DMSO-d_6) δ 13.66 (brs,1H) 8.61 (d, J = 8 Hz, 2H), 8.321 (d, J = 3 Hz, 1H), 8.01 (s, 1H), 7.98 (dd, J = 4 Hz, 8 Hz,1H), 7.75-7.68 (m, 2H), 7.64-7.58 (m, 2H).

^{13}C NMR (100 MHz, DMSO-d_6) δ 166.67, 158.76, 151.20, 143.45, 138.45, 134.91, 131.85, 131.70, 129.96, 129.63, 129.32, 128.61, 127.36, 124.36, 122.54, 100.99.

LC-MS retention time 6.27 min; LRMS m/z $[M+H]^+$: 395.3.

HRMS (ESI-TOF) m/z calcd. for $C_{18}H_{11}ClF_3N_2O_3$ $[M+H]^+$: 395.0405; found 395.0414.

Mp: 232°C–234°C

(E)-4-benzylidene-2-(2,4-dichlorophenyl)-5-methyl-2,4-dihydro-3H-pyrazol-3-one I_7

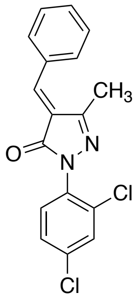

I7

The reactions were carried out following the general procedure using 2,4-dichlorophenyl)hydrazine *hydrochloride* (213.48 mg, 1 mmol) and methylacetoacetate (0.116 mg 0.108 mL, 1 mmol, 1 equiv.) for the preparation of pyrazolone core, and using *benzaldheyde* (106.12mg, 0.146 mL,1 mmol, 1 equiv.) for the last step of inhibitor synthesis.

The crude mixture was purified by crystallization with a mixture of water-acetone (5:1) and the precipitate formed was collected by filtration, washed three times with water and dried. The title compound $\mathbf{I_7}$ was isolated as a yellow solid with 55% yield over two steps (182 mg, 0.55 mmol).

^{1}H NMR (400 MHz, DMSO-d$_6$) δ 8.57-8.55 (m, 2H), 7.87 (s, 2H), 7.84-7.83 (m, 1H), 7.65-7.61 (m, 1H), 7.58-7.54 (m, 4H), 2.33 (s, 3H).

^{13}C NMR (100 MHz, DMSO-d$_6$) δ 162.35, 152.31, 149.06, 134.58, 134.01, 133.75, 133.40, 132.59, 131.19, 130.14, 129.16, 128.53, 127.62, 125.79, 13.56.

LC-MS retention time 4.07 min; LRMS m/z $[M+H]^+$: 331.3.

HRMS (ESI-TOF) m/z calcd. for $C_{17}H_{13}Cl_2N_2O$ $[M+H]^+$: 331.0399; found 331.0390.

Mp: 168°C–170°C

(E)-2-chloro-5-(4-((5-methylfuran-2-yl) methylene)-5-oxo-3-(trifluoromethyl)-4,5-dihydro-1H-pyrazol-1-yl) benzoic acid I_8

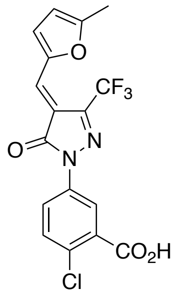

I8

The reactions were carried out following the general procedure using *2-chloro-5-hydrazinobenzoic acid hydrochloride* (223.5 mg, 1 mmol) and 4,4,4-trifluoroacetoacetate (0.146 mL, 1 mmol, 1 equiv.) for the preparation of pyrazolone core, and using 5-methyl furan-2-carbaldehyde (110.1 mg, 0.1 mL,1 mmol, 1 equiv.) for the last step of inhibitor synthesis.
The crude mixture was purified by crystallization with a mixture of water-acetone (5:1) and the precipitate formed was collected by filtration, washed three times with water and dried. The title compound **I_8** was isolated as a dark grey solid with 66% yield over two steps (263 mg, 0.66 mmol).

^{1}H NMR (400 MHz, DMSO-d$_6$) δ 13.73 (brs,1H) 8.88 (s,1H), 8.415 (d, J = 4 Hz, 2H), 8.085 (dd, J = 4 Hz, 8 Hz, 1H), 7.75-7.72 (m, 2H), 6.885 (d, J = 4 Hz, 1H), 2.61 (s,3H).

^{13}C NMR (100 MHz, DMSO-d$_6$) δ 166.52, 166.28, 161.44, 149.89, 148.73, 140.32, 140.08, 136.75, 132.04, 131.99, 131.10, 128.83, 123.09, 121.35, 114.97, 112.37, 14.89.

LC-MS retention time 4.27 min; LRMS m/z $[M+H]^+$: 399.3.

HRMS (ESI-TOF) m/z calcd. for $C_{17}H_{11}ClF_3N_2O_4$ $[M+H]^+$: 399.0354; found 399.0362.

Mp: 196 °C–198°C

(E)-2-chloro-5-(4-(3-hydroxy-4-methoxybenzylidene)-5-oxo-3-(trifluoromethyl)-4,5-dihydro-1H-pyrazol-1-yl) benzoic acid I_9

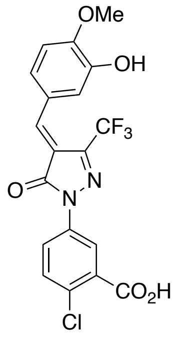

I9

The reactions were carried out following the general procedure using *2-chloro-5-hydrazinobenzoic acid hydrochloride* (223.5 mg, 1 mmol) and 4,4,4-trifluoroacetoacetate (0.146 mL, 1 mmol, 1 equiv.) for the preparation of pyrazolone core, and using 3-hydroxy-4-methoxy benzaldehyde (152.15 mg, 1 mmol, 1 equiv.) for the last step of inhibitor synthesis.
The crude mixture was purified by crystallization with a mixture of water-acetone (5:1) and the precipitate formed was collected by filtration, washed three times with water and dried. The title compound **I_9** was isolated as a yellow solid with 51% yield over two steps (225 mg, 0.51 mmol).

^{1}H NMR (400 MHz, DMSO-d_6) δ 13.60 (brs, 1H), 11.12 (brs,1H), 8.74 (s 1H), 8.30 (d, J = 3 Hz, 1H), 8.24 (d, J= 8 Hz, 1H), 8.045 (dd, J = 4 Hz, 8 Hz, 1H), 7.84 (s, 1H), 7.69 (d, J= 4 Hz, 1H), 6.99 (d, J = 8 Hz, 1H), 3.89 (s, 3H).

^{13}C NMR (100 MHz, DMSO-d_6) δ 166.60, 161.98, 156.20, 151.60, 148.05, 136.89, 134.03, 132.19, 132.03, 128.93, 125.64, 123.69, 121.87, 121.73, 118.98, 118.49, 116.72, 116.00.

LC-MS retention time 3.62 min; LRMS m/z $[M+H]^+$: 441.4.

HRMS (ESI-TOF) m/z calcd. for $C_{19}H_{13}ClF_3N_2O_5$ $[M+H]^+$: 441.0460; found 441.0449.

Mp: 226°C–227°C

(E)-2-chloro-5-(5-oxo-3-phenyl-4-((5-phenylfuran-2-yl)methylene)-4,5-dihydro-1H-pyrazol-1-yl)benzoic acid I_{10}

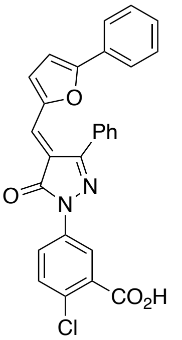

I10

The reactions were carried out following the general procedure using *2-chloro-5-hydrazinobenzoic acid hydrochloride* (223.5 mg, 1 mmol) and *ethyl 3-oxo-3-phenylpropanoate* (0.193 mg, 1 mmol, 1 equiv.) for the preparation of pyrazolone core, and using *5-phenylfuran-2-carbaldehyde* (172.18 mg, 1 mmol, 1 equiv.) for the last step of inhibitor synthesis.

The crude mixture was purified by crystallization with a mixture of water-acetone (5:1) and the precipitate formed was collected by filtration, washed three times with water and dried. The title compound $\mathbf{I_{10}}$ was isolated as a red solid with 54 % yield over two steps (253 mg, 0.54 mmol).

^{1}H NMR (400 MHz, DMSO-d_6) δ 13.60 (brs,1H), 8.785 (d, J =4 Hz, 1H), 8.495 (d, J = 4 Hz, 1H), 8.195 (dd, J = 4 Hz, 8 Hz, 1H), 8.00-7.98 (m, 2H), 7.80-7.77 (m, 2H), 7.66 (t, J = 8 Hz, 2H), 7.64-7.61 (m, 3H), 7.54-7.48 (m, 4H).

^{13}C NMR (100 MHz, DMSO-d_6) δ 162.30, 160.75, 152.41, 150.56, 149.58, 137.19, 131.40, 130.85, 130.68, 130.35, 129.71, 129.56, 129.08, 128.84, 128.77, 128.73, 128.63, 128.46, 125.81, 125.47, 119.15, 112.22, 109.27.

LC-MS retention time 6.05 min; LRMS m/z $[M+H]^+$: 469.4.

HRMS (ESI-TOF) m/z calcd. for $C_{27}H_{18}ClN_2O_4$ $[M+H]^+$: 469.0950; found 469.0961.
Mp: 268°C–269°C

2-chloro-5-((E)-5-oxo-4-((E)-3-phenylallylidene)-3-(trifluoromethyl)-4,5-dihydro-1H-pyrazol-1-yl) benzoic acid I_{11}

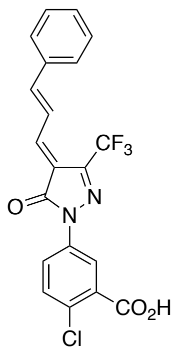

I11

The reactions were carried out following the general procedure using *2-chloro-5-hydrazinobenzoic acid hydrochloride* (223.5 mg, 1 mmol) and 4,4,4-trifluoroacetoacetate (0.146 mL, 1 mmol, 1 equiv.) for the preparation of pyrazolone core, and using cinnamaldehyde (132.16 mg, 0.125 mL, 1 mmol, 1 equiv.) for the last step of inhibitor synthesis.
The crude mixture was purified by crystallization with a mixture of water-acetone (5:1) and the precipitate formed was collected by filtration, washed three times with water and dried. The title compound **I_{11}** was isolated as a red solid with 82% yield over two steps (345 mg, 0.82 mmol).
^1H NMR (400 MHz, DMSO-d$_6$) δ 13.6 (brs, 1H), 8.54-8.47 (m, 1H), 8.345 (d, J = 4 Hz, 1H), 8.01-7.93 (m, 3H), 7.76-7.74 (m, 2H), 7.68 (d, J = 8 Hz, 1H), 7.57-7.51 (m, 3H).
^{13}C NMR (100 MHz, DMSO-d$_6$) δ 166.46, 162.42, 156.03, 149.79, 140.23, 139.85, 136.57, 135.45, 132.76, 132.06, 129.98, 129.68, 128.95, 123.22, 122.92, 121.49, 121.33, 118.36.
LC-MS retention time 4.72 min; LRMS m/z $[M+H]^+$: 421.3.
HRMS (ESI-TOF) m/z calcd. for $C_{20}H_{13}ClF_3N_2O_3$ $[M+H]^+$: 421.0561; found 421.0571.
Mp: 238°C–240°C

(E)-2-chloro-5-(4-(naphthalen-2-ylmethylene)-5-oxo-3-(trifluoromethyl)-4,5- dihydro-1H-pyrazol-1-yl)benzoic acid I_{12}

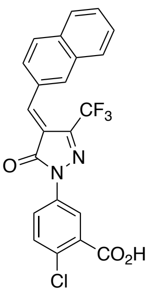

I12

The reactions were carried out following the general procedure using *2-chloro-5-hydrazinobenzoic acid hydrochloride* (223.5 mg, 1 mmol) and 4,4,4-trifluoroacetoacetate (0.146 mL, 1 mmol, 1 equiv.) for the preparation of pyrazolone core, and using 2-naphtaldehyde (156.18 mg, 1 mmol, 1 equiv.) for the last step of inhibitor synthesis.

The crude mixture was purified by crystallization with a mixture of water-acetone (5:1) and the precipitate formed was collected by filtration, washed three times with water and dried. The title compound **I₁₂** was isolated as a red-orange solid with 40% yield over two steps (178 mg, 0.4 mmol).

^{1}H NMR (400 MHz, DMSO-d$_6$) δ 13.7 (brs, 1H), 9.18 (brs, 1H), 8.79 (dd, J = 4 Hz, 8 Hz, 1H), 8.33 (d, J = 4 Hz, 1H), 8.15 (s, 1H), 8.11-8.08 (m, 2H), 8.05-8.01 (m, 2H), 7.78-7.74 (m, 1H), 7.70 (d, J = 8 Hz, 1H), 7.69-7.65 (m, 1H).

^{13}C NMR (100 MHz, DMSO-d$_6$) δ 166.54, 161.45, 151.05, 138.65, 136.52, 135.99, 135.02, 132.69, 132.16, 132.05, 130.47, 130.06, 129.11, 129.07, 128.69, 128.69, 128.29, 127.81, 123.68, 121.86, 121.18, 119.23.

LC-MS retention time 7.1 min; LRMS m/z [M+H]$^+$: 445.3.

HRMS (ESI-TOF) m/z calcd. for $C_{22}H_{13}ClF_3N_2O_3$ [M+H]$^+$: 445.0561; found 445.0550.

Mp: 238°C–239°C

(E)-5-(4-([1,1'-biphenyl]-4-ylmethylene)-5-oxo-3-(trifluoromethyl)-4,5- dihydro-1H-pyrazol-1-yl)-2-chlorobenzoic acid I$_{13}$

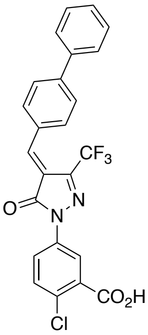

I13

The reactions were carried out following the general procedure using 2-chloro-5-hydrazinobenzoic acid hydrochloride (223.5 mg, 1 mmol) and 4,4,4-trifluoroacetoacetate (0.146 mL, 1 mmol, 1 equiv.) for the preparation of pyrazolone core, and using biphenyl-4-carboxaldehyde (182.22 mg, 1 mmol, 1 equiv.) for the last step of inhibitor synthesis.

The crude mixture was purified by crystallization with a mixture of water-acetone (5:1) and the precipitate formed was collected by filtration, washed three times with water and dried. The title compound **I_{13}** was isolated as a red-orange solid with 64% yield over two steps (301 mg, 0.64 mmol).

^{1}H NMR (400 MHz, DMSO-d$_6$) δ 13.68 (s, 1H), 8.76 (d, J = 4 Hz, 2H), 8.35 (d, J = 2.7 Hz, 1H), 8.04 (s, 1H), 8.00 (dd, J = 4, 8 Hz, 1H), 7.97 (d, J = 8 Hz, 2H), 7.87-7.85 (m, 2H), 7.69 (d, J = 8 Hz, 1H), 7.57-7.52 (m, 2H), 7.50-7.46 (m, 1H).

^{13}C NMR (100 MHz, DMSO-d$_6$) δ 166.50, 161.46, 150.56, 146.47, 138.87, 136.52, 135.96, 132.05, 131.93, 129.68, 129.50, 129.11, 127.85, 127.62, 127.44, 127.34, 127.29, 123.56, 121.82, 120.87.

LC-MS retention time 6.78 min; LRMS m/z $[M+H]^+$: 471.4.

HRMS (ESI-TOF) m/z calcd. for $C_{24}H_{15}ClF_3N_2O_3$ $[M+H]^+$: 471.0718; found 471.0727.

Mp: 255°C–257°C

2-chloro-5-(3-methyl-5-oxo-4-((5-phenylfuran-2-yl)methylene)-4,5-dihydro-1H-pyrazol-1-yl)benzoic acid I_{14}

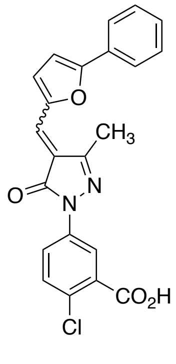

I14

The reactions were carried out following the general procedure using *2-chloro-5-hydrazinobenzoic acid hydrochloride* (223.5 mg, 1 mmol) and methylacetoacetate (0.116 mg 0.108 mL, 1 mmol, 1 equiv.) for the preparation of pyrazolone core, and using *5-phenylfuran-2-carbaldehyde* (172.18 mg, 1 mmol, 1 equiv.) for the last step of inhibitor synthesis.

The crude mixture was purified by crystallization with a mixture of water-acetone (5:1) and the precipitate formed was collected by filtration, washed three times with water and dried. The title compound **I$_{14}$** was isolated as a red solid with 40 % yield over two steps (162 mg, 0.4 mmol) as a mixture of E/Z stereoisomers.

^{1}H NMR and ^{13}C NMR signals corresponding to the major stereoisomer are indicated with *, in contrast, the signals corresponding to minor ones were indicated with §.

^{1}H NMR (400 MHz, DMSO d$_6$) δ 13.56 (s, 1H), 8.685 (d, J =4 Hz, 1H), 8.42 (bs, 1H), 8.37 (bs 1H), 8.115 (brs 2H), 7.99-7.97 (m, 2H), 7.925-7.90 (m, 1H), 7.835 (d, J = 4 Hz, 1H), 7.77 (s, 1H), 7.61 (d, J = 8 Hz, 2H), 7.57-7.54 (m,3H), 7.51-7.48 (m,3H), 2.75 § (s, 3H), 2.36* (s,3H).

^{13}C NMR (100 MHz, DMSO-d$_6$) δ 166.82*, 166.80§, 165.13§, 162.27*, 161.66§, 160.26*, 151.98*, 150.43*, 149.94§, 149.02§, 137.56*, 137.37§, 132.05§, 131.97*, 131.84§, 131.77§, 131.71*, 130.62, 130.52, 130.35, 129.82, 129.73, 128.89, 128.85, 128.52, 127.27, 126.97, 126.93, 125.71, 125.59, 121.66*, 121.41§, 120.61*, 119.99*, 119.72§, 119.01§, 111.93*, 111.75§, 18.29§, 13.31*.

LC-MS retention time 4.65 min; LRMS m/z [M+H]$^+$: 407.4.

HRMS (ESI-TOF) m/z calcd. for $C_{22}H_{16}ClN_2O_4$ [M+H]$^+$: 407.0793; found 407.07998.

Mp: 262°C–263°C

4. **Synthetic procedure and characterization data for Methyl (E)-2-chloro-5-(5-oxo-4-((5-phenylfuran-2-yl)methylene)-3 (trifluoromethyl)-4,5-dihydro-1H-pyrazol-1-yl)benzoate I$_6$**

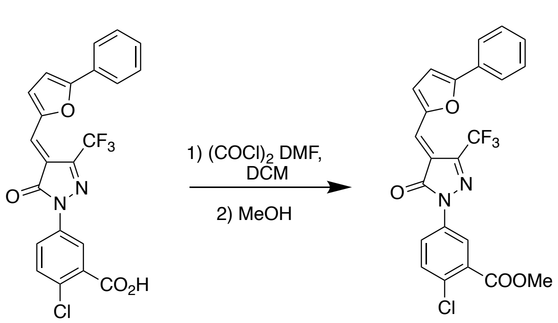

I6

Oxalyl chloride (0.021 mL, 0.24 mmol, 2.6 equiv.) was added to a mixture of EN460 (50 mg, 0.1 mmol, 1 equiv.) in anhydrous dichloromethane (1mL) in a cold bath with a catalytic amount of DMF (2 drops). After three hours, 2 mL of methanol was added, and the reaction mixture

was stirred for 2 hours at room temperature. Then the crude mixture was purified by column chromatography (dichloromethane:methanol from 100:0 to 98:2) to afford the title compound **I₆** as a red solid with 77% Yield (37 mg, 0.077 mmol).

^{1}H NMR (400 MHz, DMSO-d$_6$) δ 8.84 (*s,* 1H) 8.365 (d, J = 4 Hz, 1H), 8.13-8.09 (m, 3H), 7.65 (s, 1H), 7.74 (d, J= 8 Hz, 1H), 7.655 (d, J= 4Hz, 1H), 7.61-7.55 (m, 3H), 3.93 (s,3H.).

^{13}C NMR (100 MHz, DMSO-d$_6$) δ 165.37, 163.22, 150.53, 136.87, 132.19, 131.59, 130.84, 129.96, 129.88, 129.70, 129.11, 128.95, 128.36, 126.42, 125.48, 124.97, 123.88, 121.63, 113.42, 109.28, 53.31.

LC-MS retention time 6.28 min; LRMS m/z [M+H]$^+$: 475.3.

HRMS (ESI-TOF) m/z calcd. for $C_{23}H_{15}ClF_3N_2O_4$ [M+H]$^+$: 475.0677; found 475.0688.

Mp: 208°C–210°C

5. Synthetic procedure and characterization data for 2-chloro-5-(5-oxo-4-((5-phenylfuran-2-yl)methyl)-3-(trifluoromethyl)-4,5-dihydro-1H-pyrazol-1-yl)benzoic acid I$_{15}$

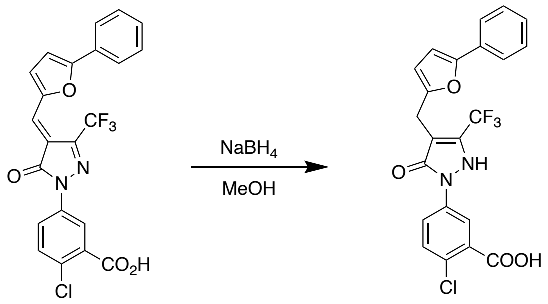

I15

Sodium borohydride (28.18mg, 0.745 mmol, 5 equiv.) was added to a mixture of **EN460** (69 mg, 0.149 mmol, 1 equiv.) in methanol (9.5 mL) at 0°C. After one hour, the reaction was quenched with the addition of a saturated solution of ammonium chloride and diluted with dichloromethane. The phases were separated and the organic layer was dried with sodium sulfate and evaporated under reduced pressure to afford the crude product. The crude mixture was purified by crystallization with a mixture of water-acetone (5:1) and the precipitate formed was collected by filtration, washed three times with water and dried. The title compound **I$_{15}$** was isolated as an orange solid with 50% yield (34 mg, 0.073 mmol).

^{1}H NMR (400 MHz, DMSO-d$_6$) δ 8.365 (d, *J* = 4 Hz, 1H) 8.205 (dd, J = 4 Hz, 8 Hz, 1H), 7.62-7.60 (m, 2H), 7.38 (t, *J* = 8 Hz, 2H), 7.24-7.20 (m, 2H), 6.745 (d, J = 4 Hz, 1H), 5.955 (d, J = 4 Hz, 1H), 3.60 (s, 2H), 1.89 (s, 1H).

^{13}C NMR (100 MHz, DMSO-d$_6$) δ 163.96, 157.22, 151.12, 140.45, 139.41, 139.18, 131.60, 131.32, 129.36, 129.22, 127.09, 124.62, 123.56, 123.22, 122.84, 119.41, 107.27, 106.98, 22.14.

LC-MS retention time 5.42 min; LRMS m/z $[M+H]^+$: 463.3.

HRMS (ESI-TOF) m/z calcd. for $C_{22}H_{15}ClF_3N_2O_4$ $[M+H]^+$: 463.0667; found 463.0659.

Mp: 148°C–150°C

6. Synthetic procedure and characterization data for (R)-S-(2-(2-Acetamido-3-mercaptopropanamido)ethyl) Ethane- thioate I_{16}

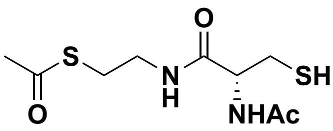

I16

I_{16} was synthesized according to the literature procedure.[2]

^1H NMR (400 MHz, $CDCl_3$) δ 7.17 (t, J= 5.5Hz, 1H), 6.80 (d, J= 8.0 Hz, 1H), 4.65 (ddd, J = 8.0, 6.5, 4.5 Hz, 1H), 3.55−3.34 (m, 2H), 3.03 (t, J = 6.5 Hz, 2H), 2.99 (ddd, J = 14.0, 8.0, 4.5 Hz, 1H), 2.76 (ddd, J = 14.0, 10.0, 6.5 Hz, 1H), 2.35 (s, 3H), 2.06 (s, 3H), 1.60 (dd, J = 10.0, 8.0 Hz, 1H).

^{13}C NMR (100 MHz, $CDCl_3$): δ 196.3, 170.4, 170.0, 54.2, 39.6, 30.7, 28.5, 26.8, 23.2;

$[\alpha]_D^{25}$= −37 (c = 0.87, $CHCl_3$).

HRMS (ESI-TOF) m/z calcd. for $C_9H_{17}N_2O_3S_2$ $[M+H]^+$: 265.0675; found 265.068.

Mp = 126−128 °C.

The chemical−physical data are in accordance with the literature.[2]

7. References

[1] K. E. Hayes, P. Batsomboon, W. C. Chen, B. D. Johnson, A. Becker, S. Eschrich, Y. Yang, A. R. Robart, G. B. Dudley, W. J. Geldenhuys, L. A. Hazlehurst, Inhibition of the FAD containing ER oxidoreductin 1 (Ero1) protein by EN-460 as a strategy for the treatment of multiple myeloma, *Bioorganic & Medicinal Chemistry,* **2019**, 27,1479-1488.

[2] F. Bartoccini, M. Retini, R. Crinelli, M. Menotta, A. Fraternale, G. Piersanti, Dithiol Based on L-Cysteine and Cysteamine as a Disulfide-Reducing Agent J. Org. Chem. **2022**, 87, 10073–10079.

8. NMR spectra

^{1}H-NMR and ^{13}C-NMR of ***(E)-5-(4-((5-phenylfuran-2-yl)methylene)-5-oxo-3-(trifluoromethyl)-4,5-dihydro-1H-pyrazol-1-yl)-2-chlorobenzoic acid EN460***

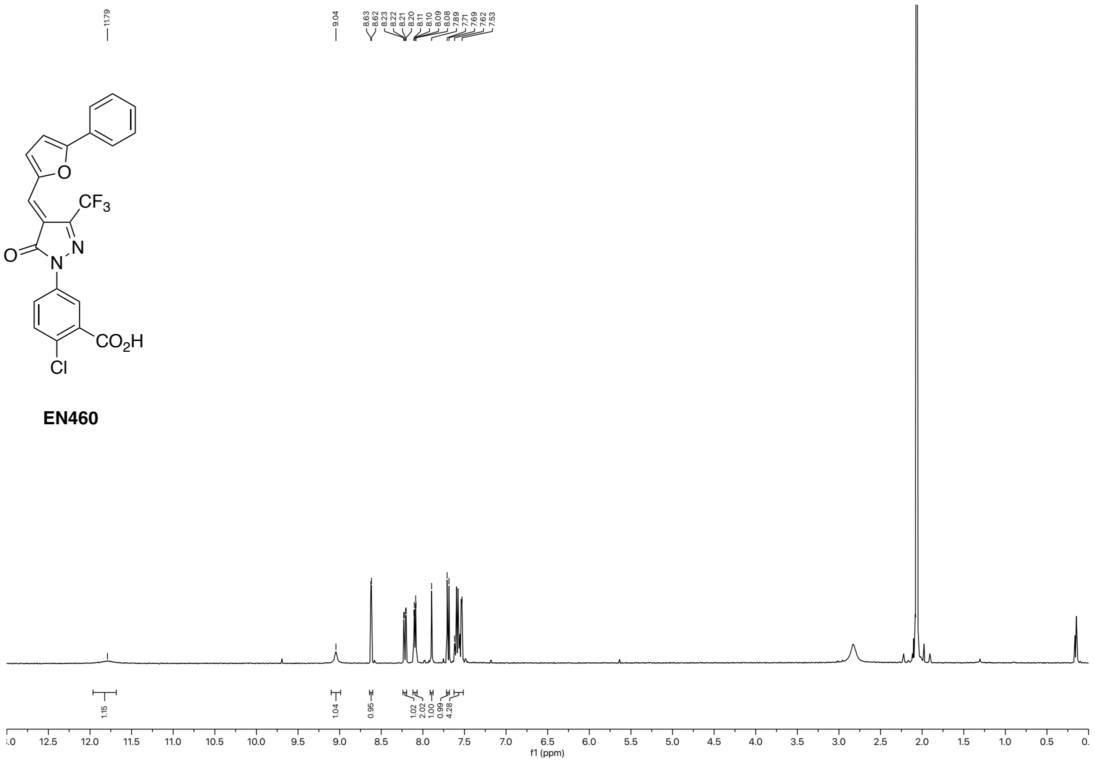

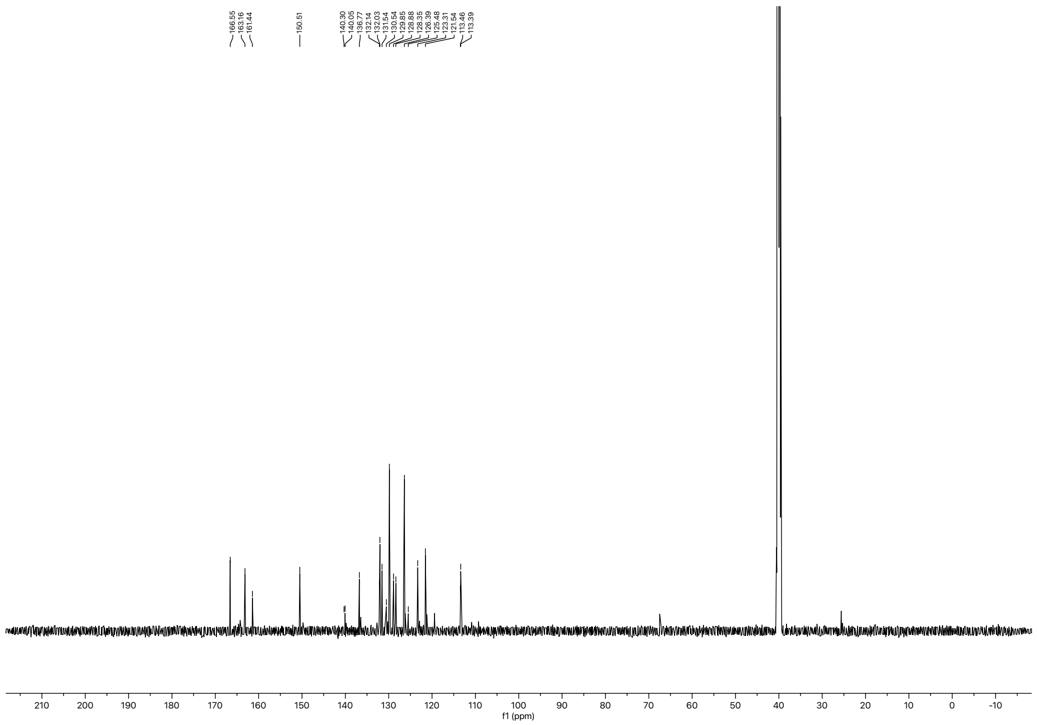

^{1}H-NMR and ^{13}C-NMR of (E)-2-(4-chlorophenyl)-4-((5-phenylfuran-2-yl) methylene)-5-(trifluoromethyl)-2,4-dihydro-3H-pyrazol-3-one I$_1$

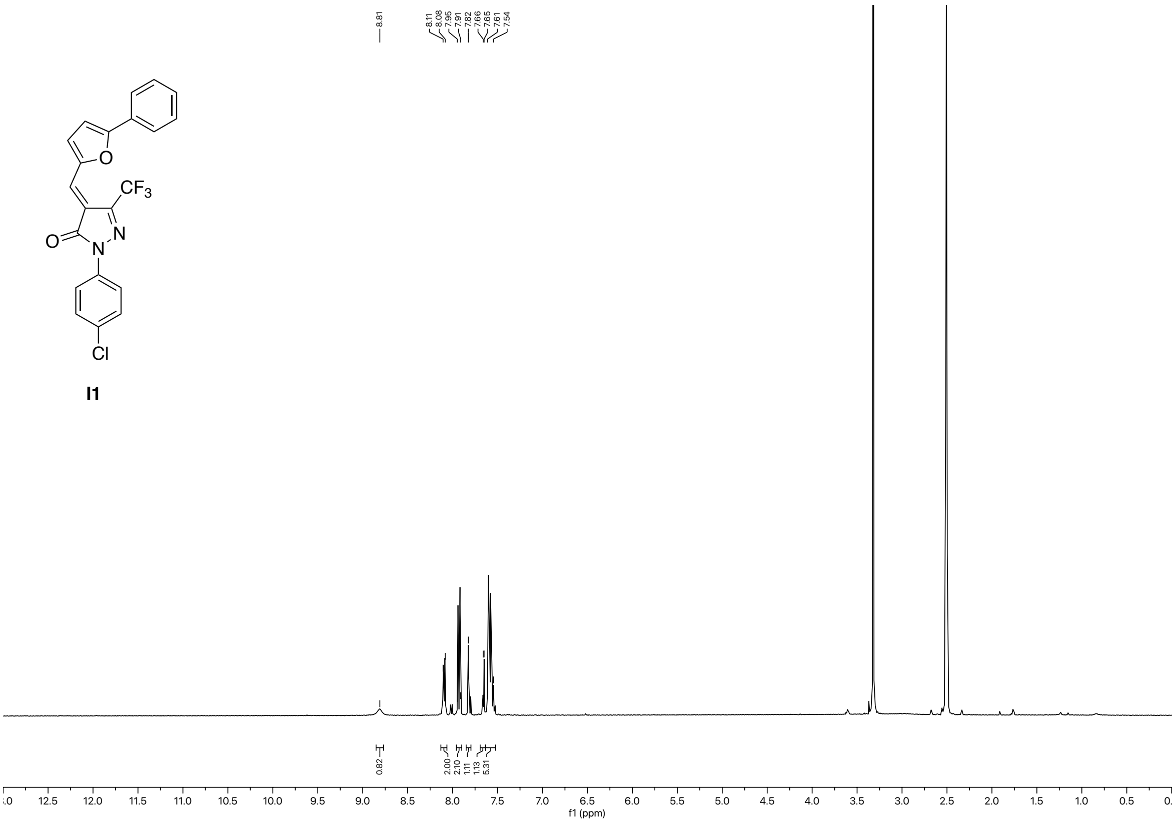

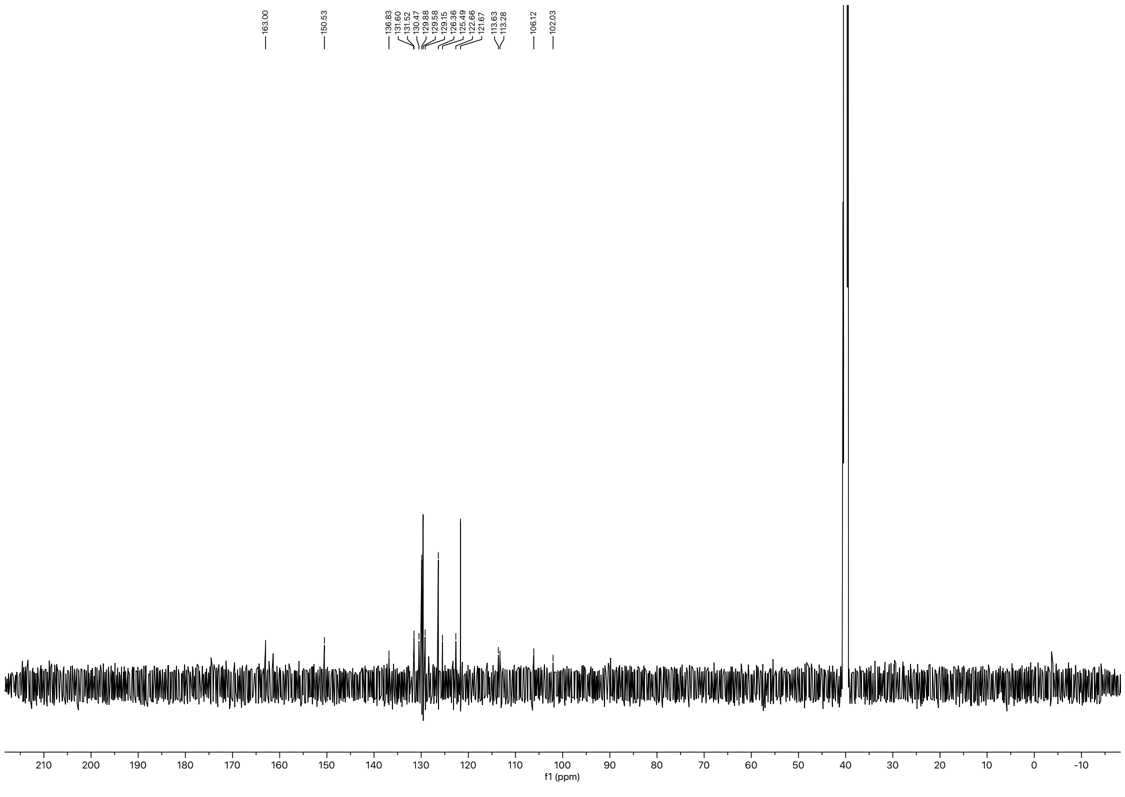

^{1}H-NMR and ^{13}C-NMR of (E)-2-(2,4-dichlorophenyl)-4-((5-phenylfuran-2-yl)methylene)-5 (trifluoromethyl)-2,4-dihydro-3H-pyrazol-3-one I_2

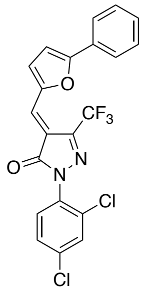

I2

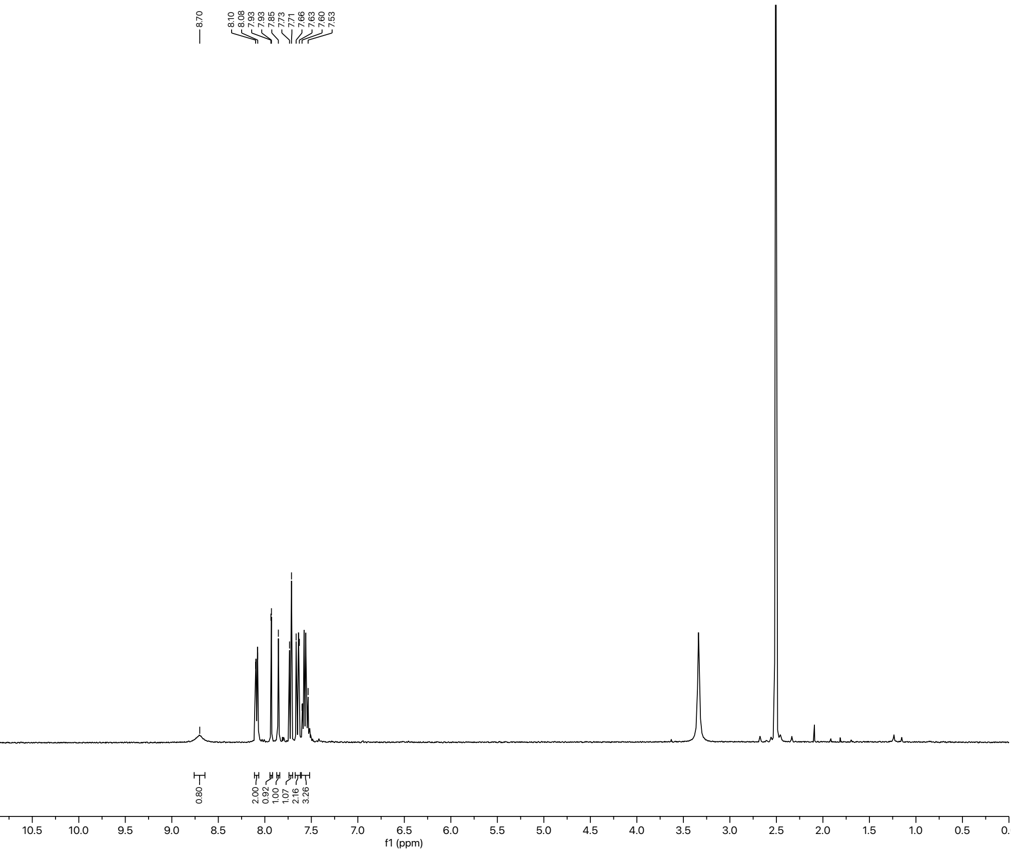

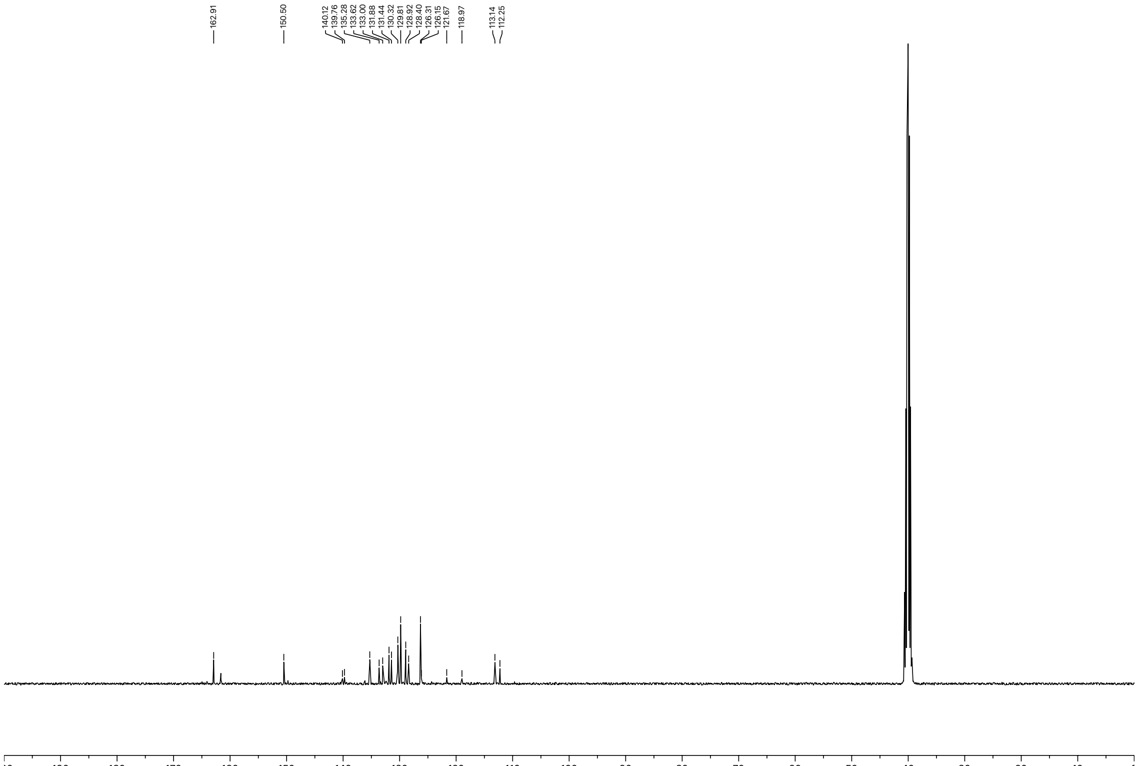

^{1}H-NMR and ^{13}C-NMR of (E)-3-(5-oxo-4-((5-phenylfuran-2-yl)methylene)-3-(trifluoromethyl)-4,5-dihydro-1H-pyrazol-1-yl)benzoic acid I$_3$

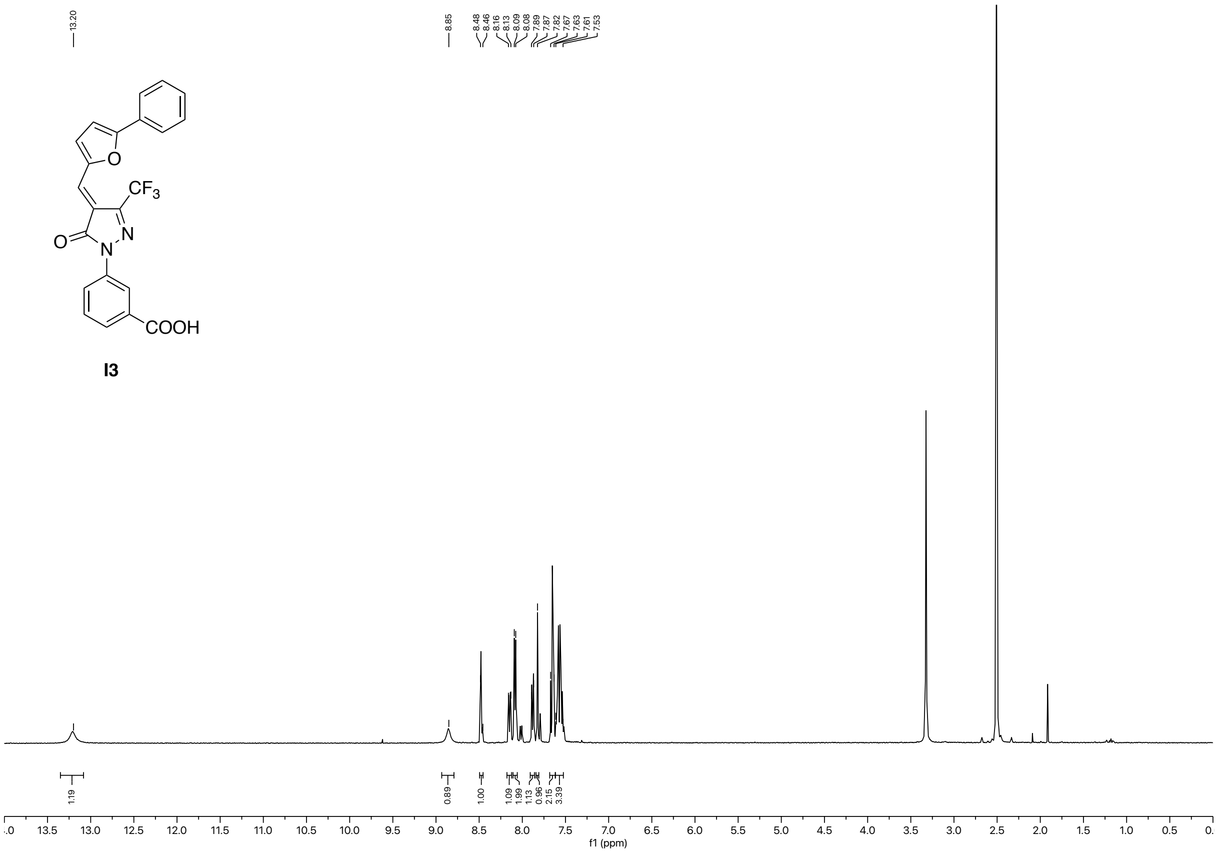

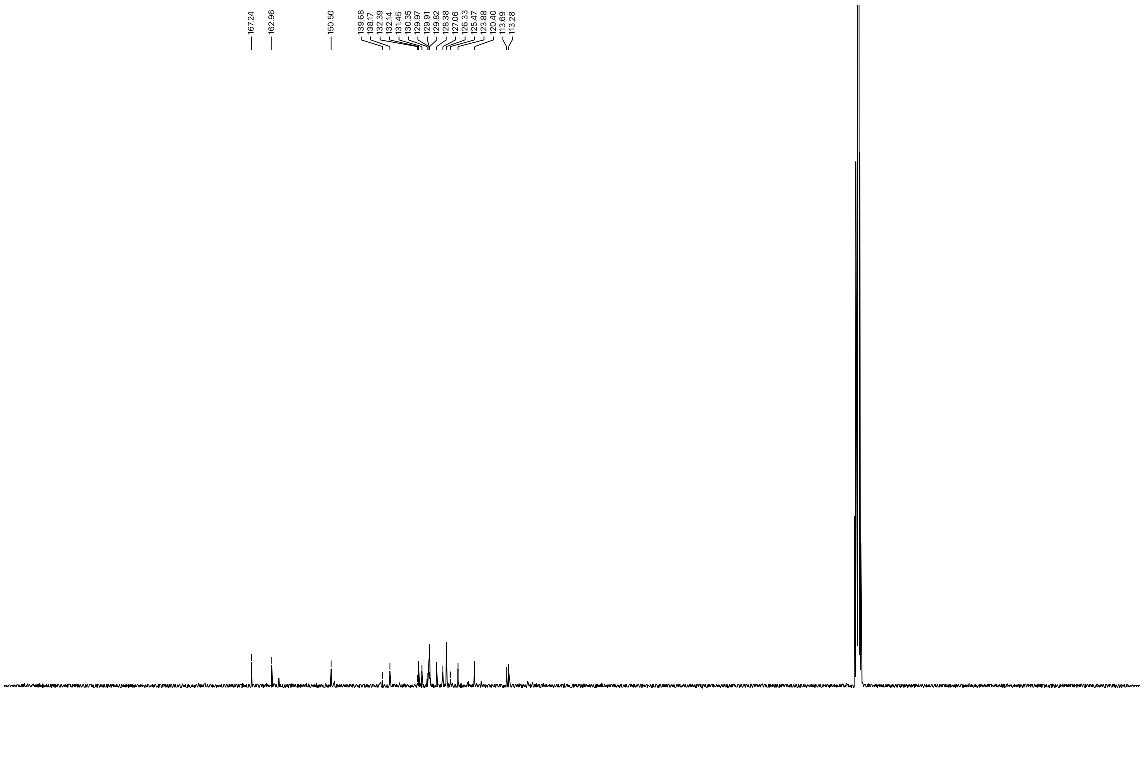

^{1}H-NMR and ^{13}C-NMR of (E)-4-(5-oxo-4-((5-phenylfuran-2-yl)methylene)-3-(trifluoromethyl)-4,5-dihydro-1H-pyrazol-1-yl)benzoic acid I$_4$

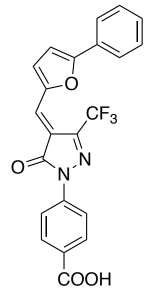

I4

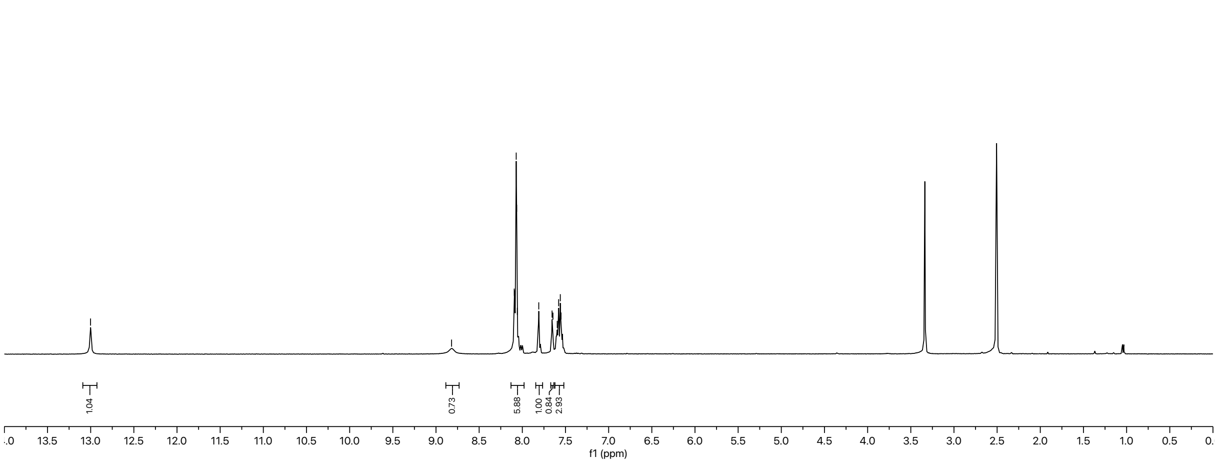

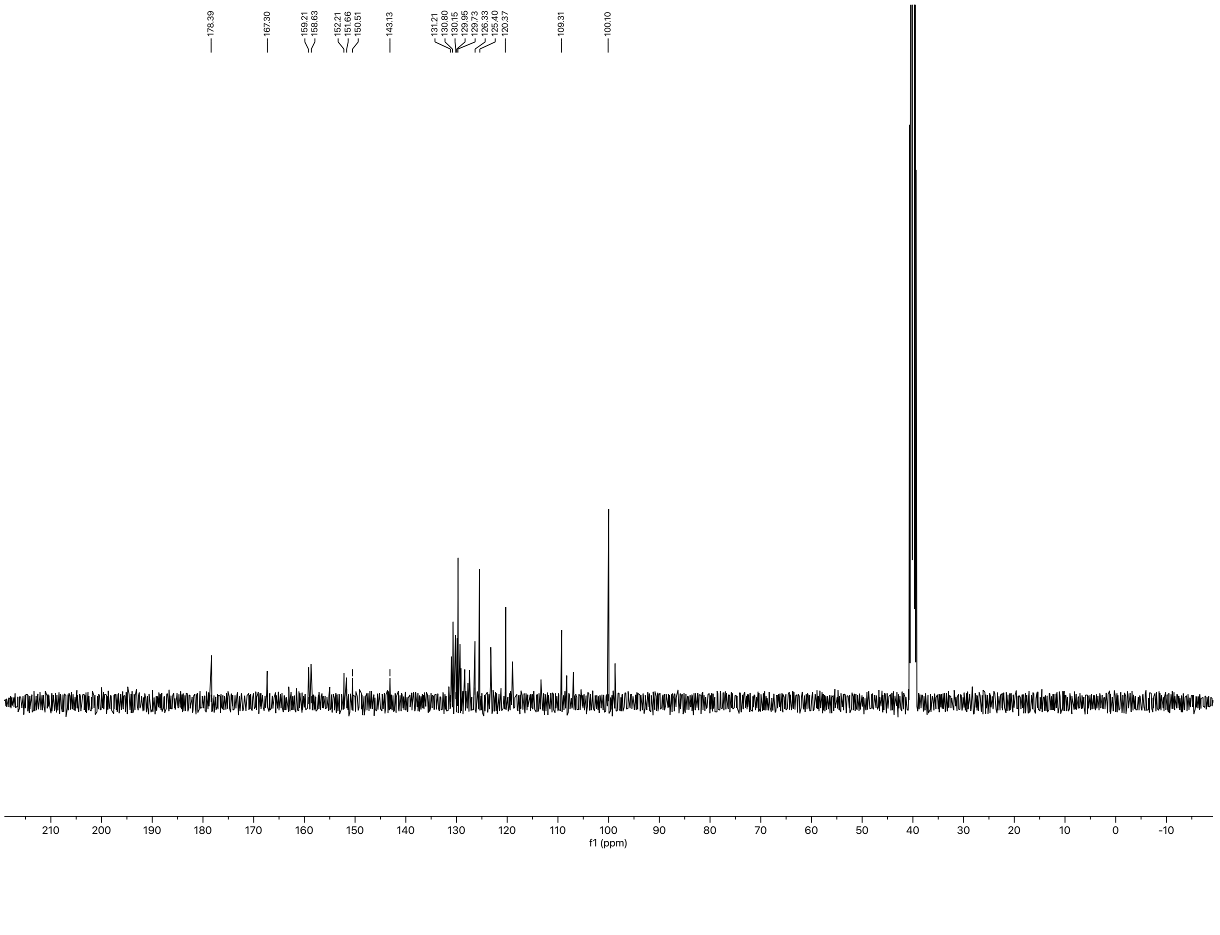

^{1}H-NMR and ^{13}C-NMR of (E)-5-(4-benzylidene-5-oxo-3-(trifluoromethyl)-4,5-dihydro-1H-pyrazol-1-yl)-2-chlorobenzoic acid I$_5$

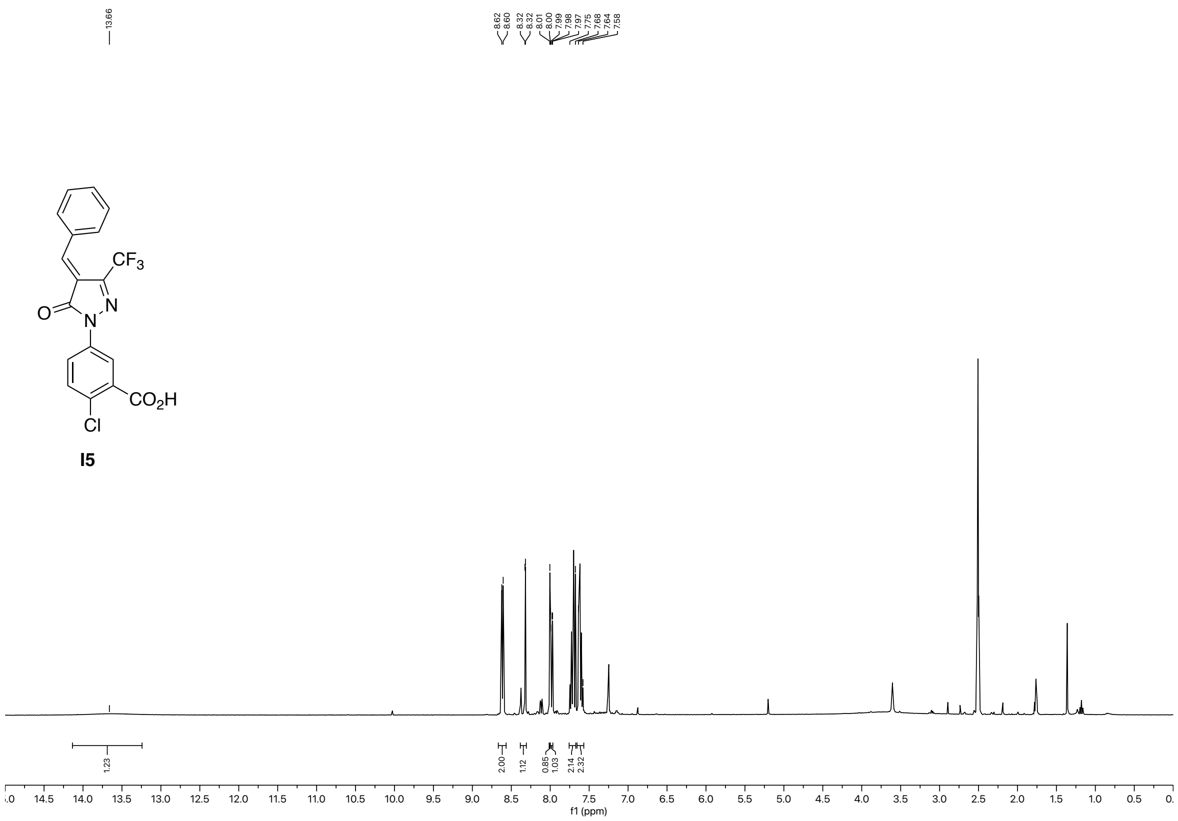

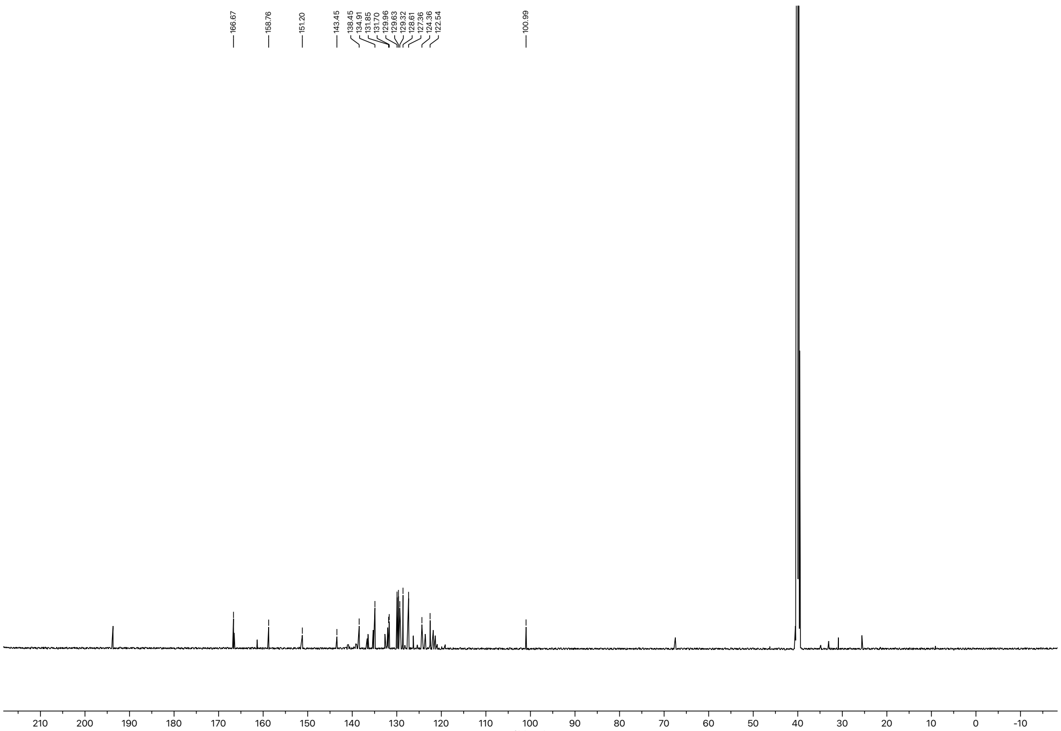

^{1}H-NMR and ^{13}C-NMR of (E)-4-benzylidene-2-(2,4-dichlorophenyl)-5-methyl-2,4-dihydro-3H-pyrazol-3-one I$_7$

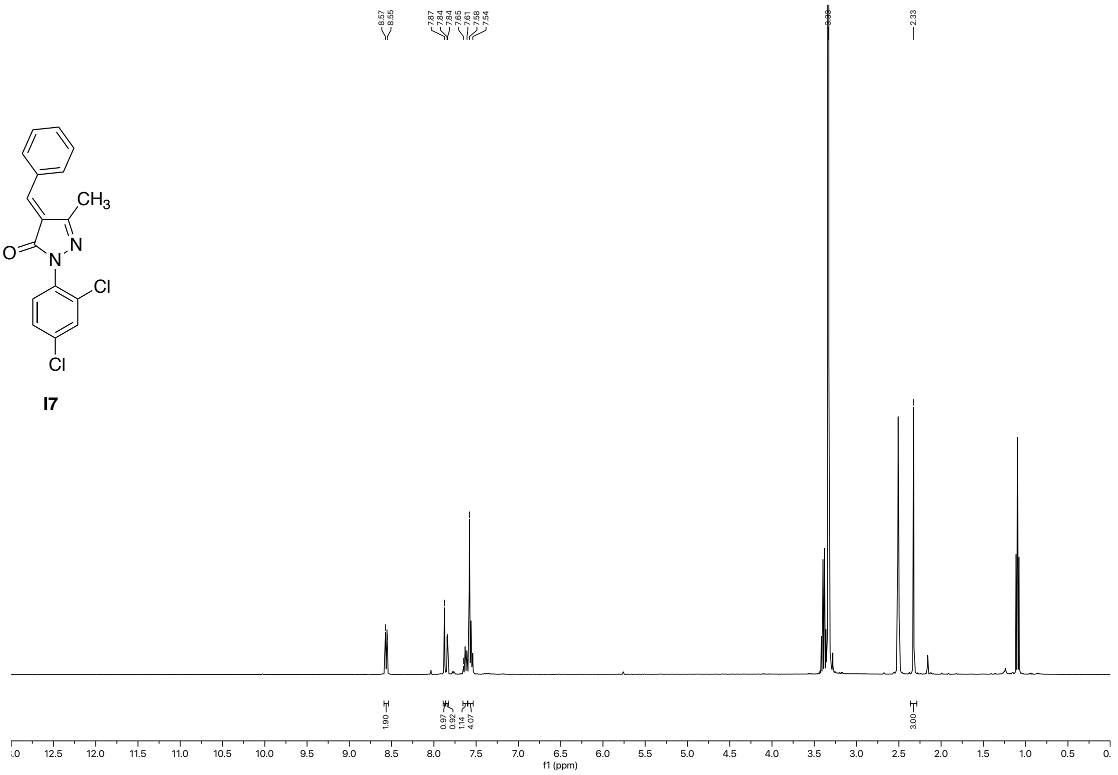

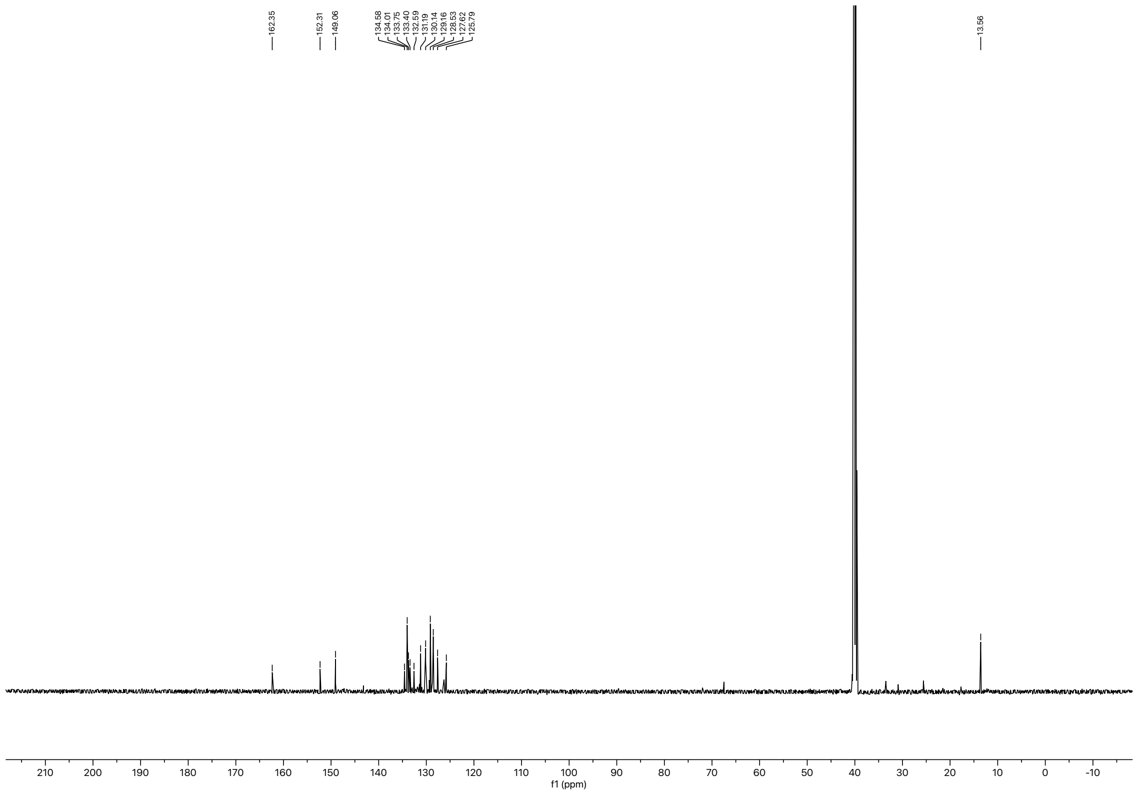

^{1}H-NMR and ^{13}C-NMR of (E)-2-chloro-5-(4-((5-methylfuran-2-yl) methylene)-5-oxo-3-(trifluoromethyl)-4,5-dihydro-1H-pyrazol-1-yl) benzoic acid I_8

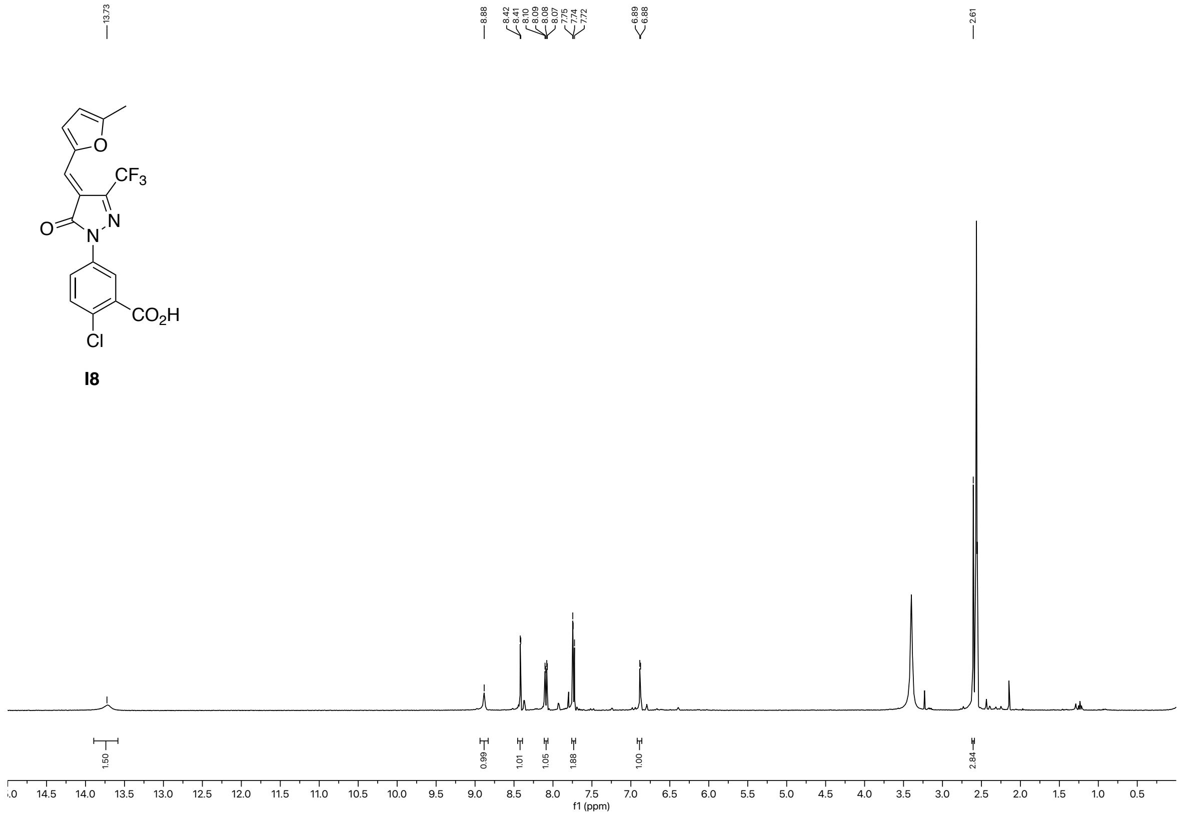

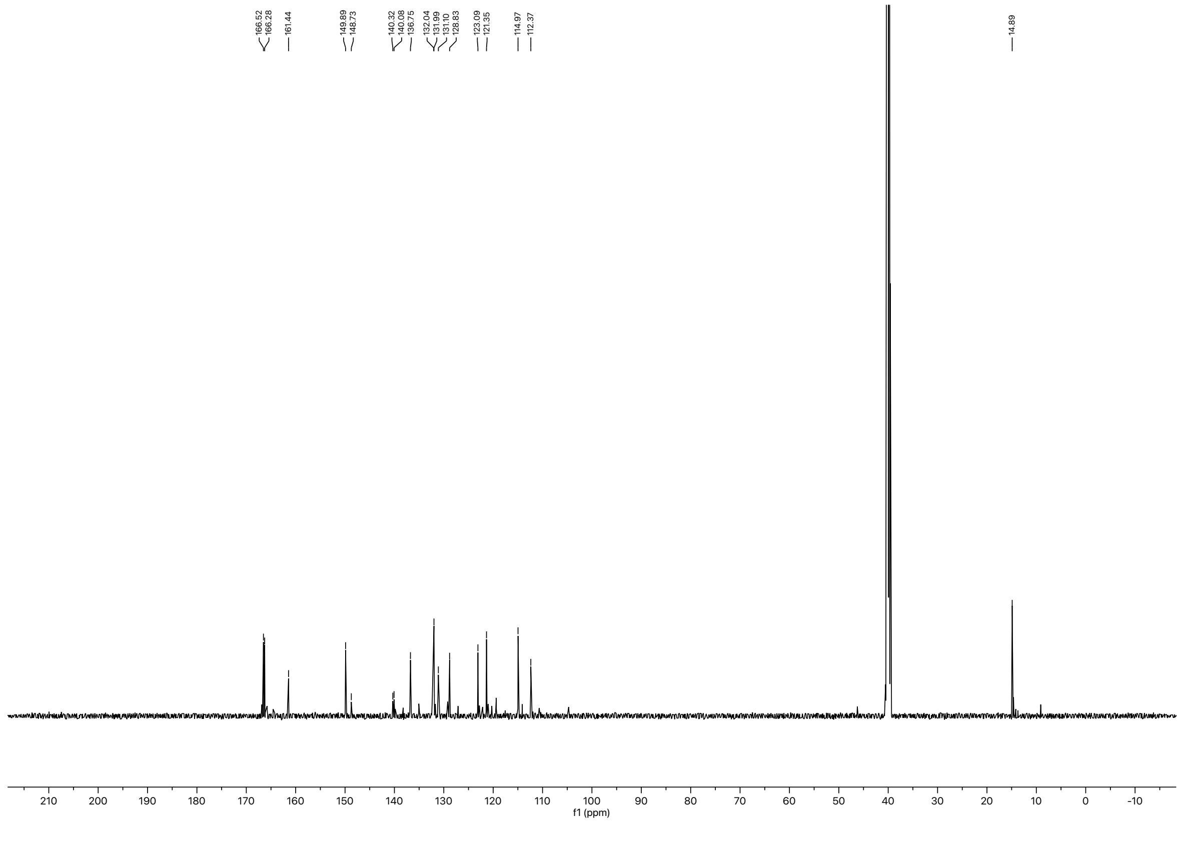

^{1}H-NMR and ^{13}C-NMR of (E)-2-chloro-5-(4-(3-hydroxy-4-methoxybenzylidene)-5-oxo-3-(trifluoromethyl)-4,5-dihydro-1H-pyrazol-1-yl) benzoic acid I_9

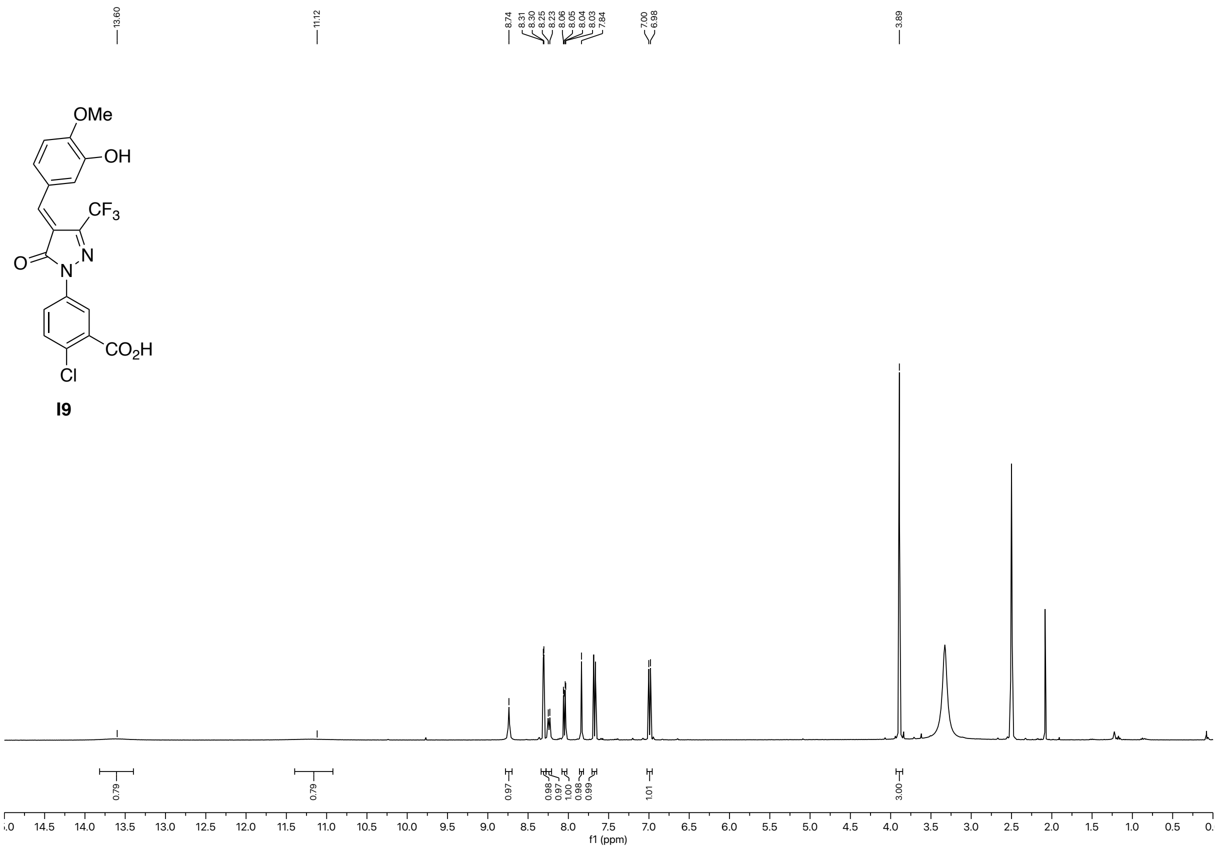

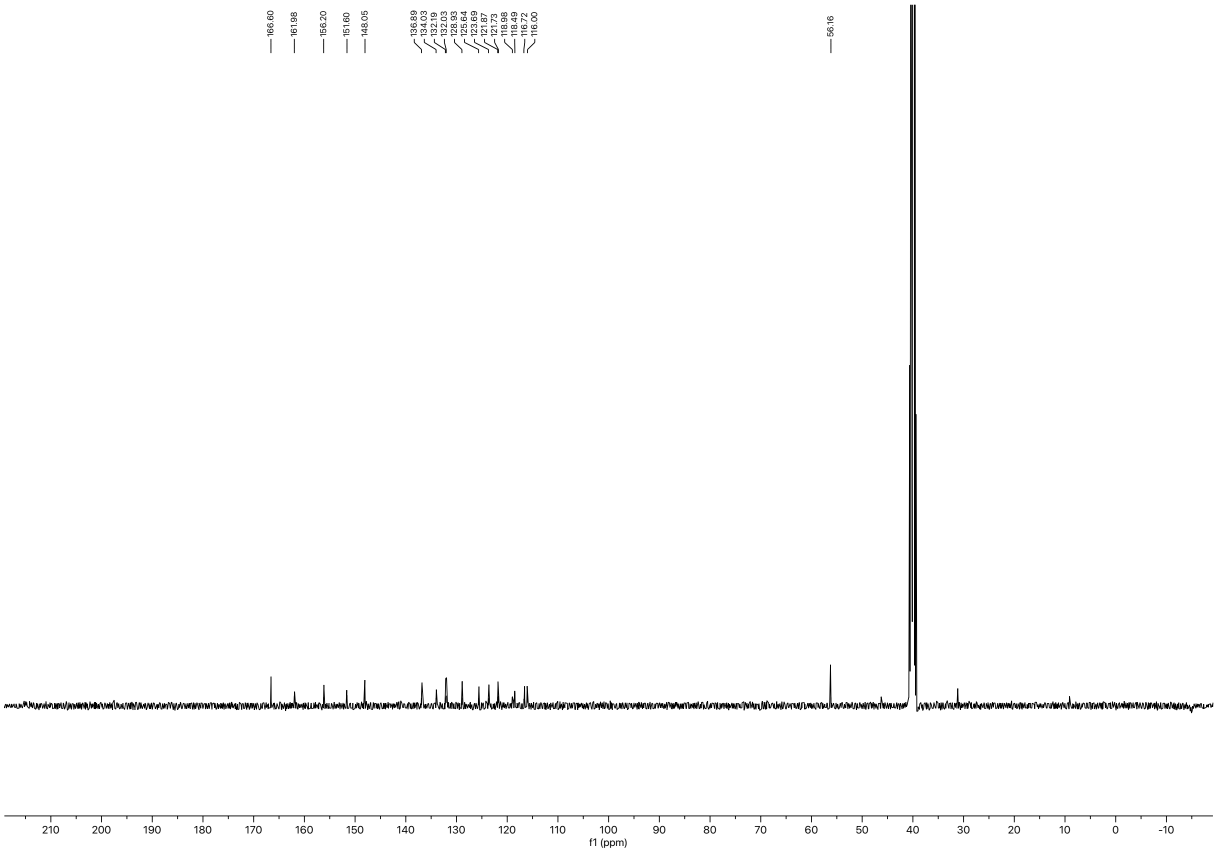

^{1}H-NMR and ^{13}C-NMR of (E)-2-chloro-5-(5-oxo-3-phenyl-4-((5-phenylfuran-2-yl)methylene)-4,5-dihydro-1H-pyrazol-1-yl)benzoic acid I_{10}

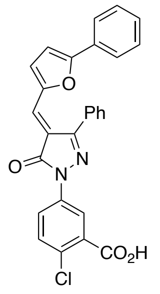

I10

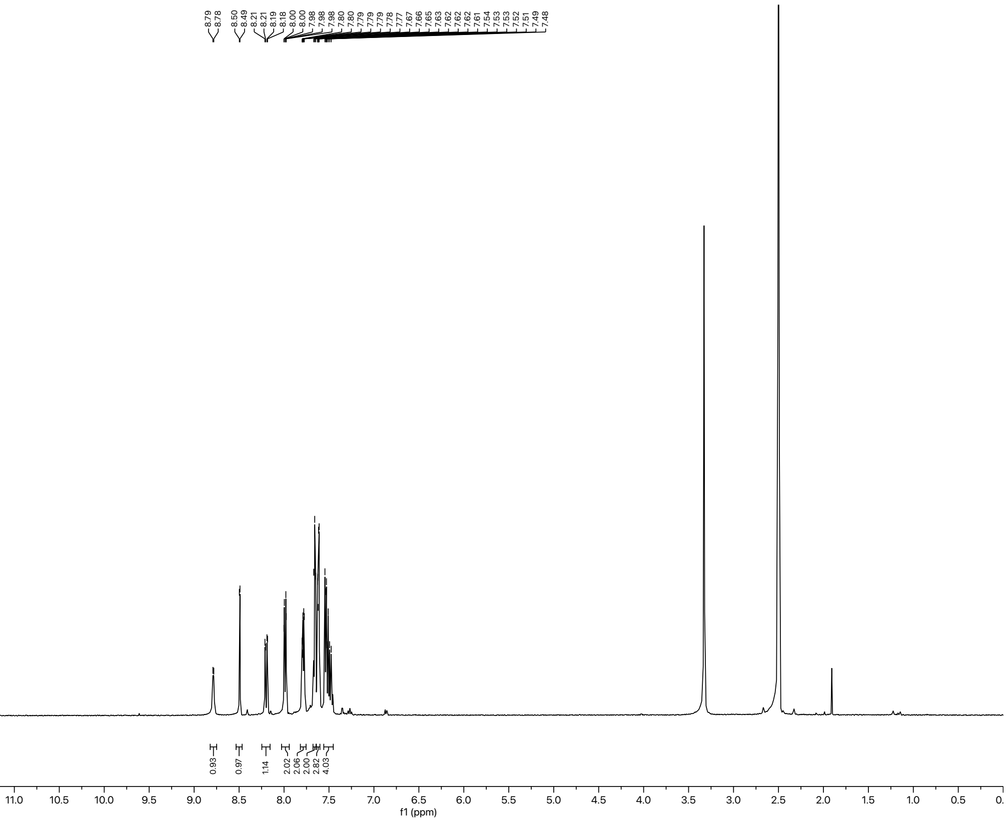

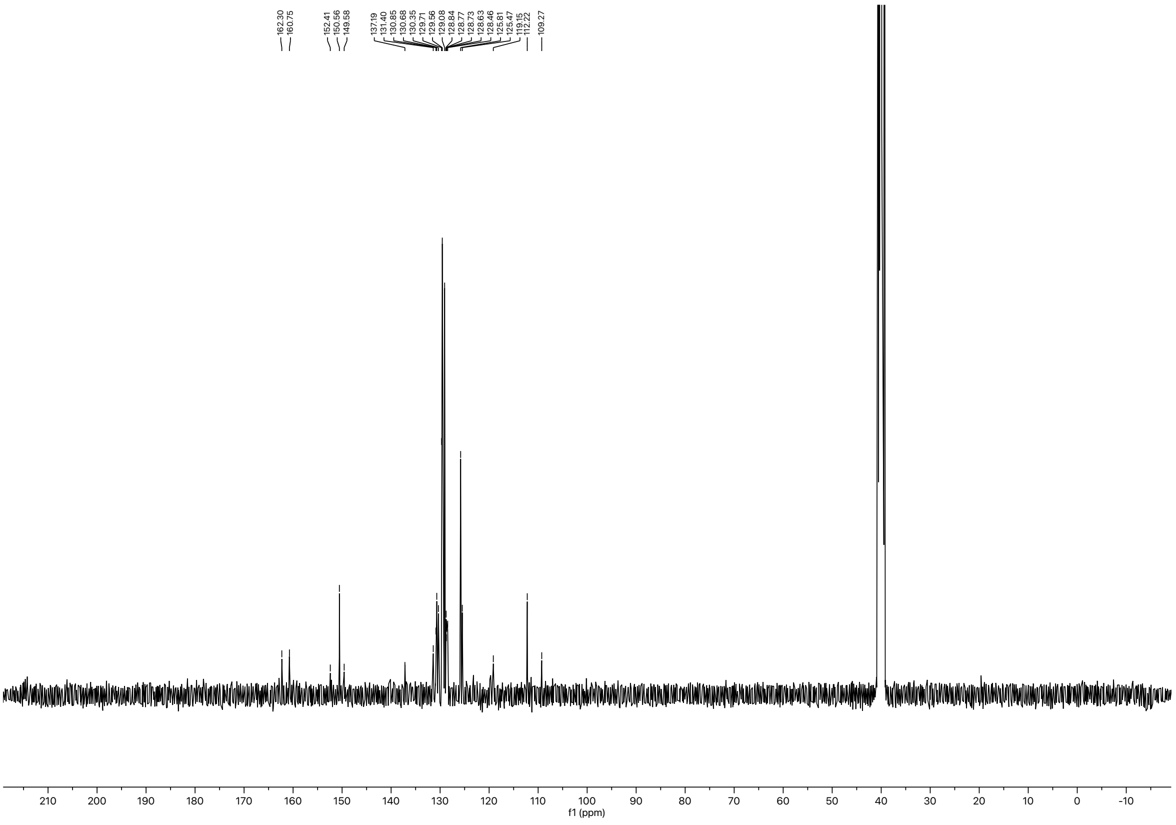

^{1}H-NMR and ^{13}C-NMR of 2-chloro-5-((E)-5-oxo-4-((E)-3-phenylallylidene)-3-(trifluoromethyl)-4,5-dihydro-1H-pyrazol-1-yl) benzoic acid I_{11}

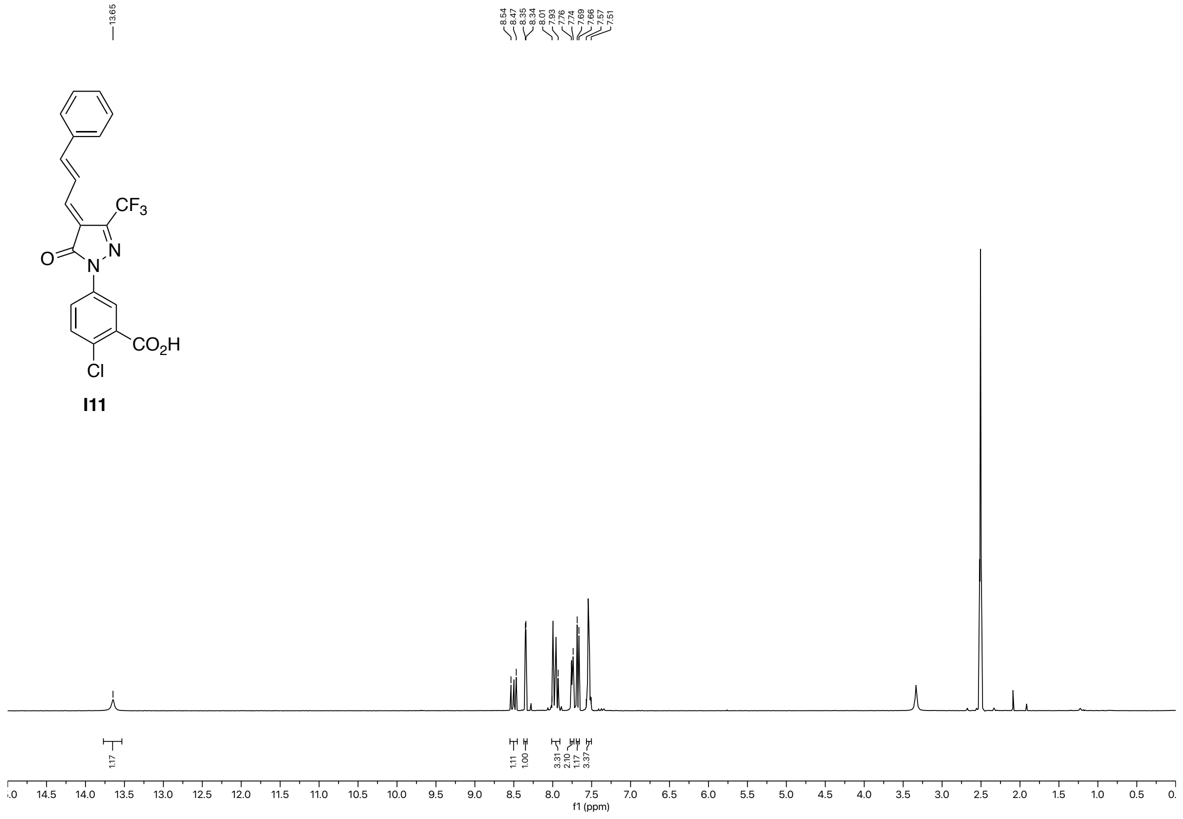

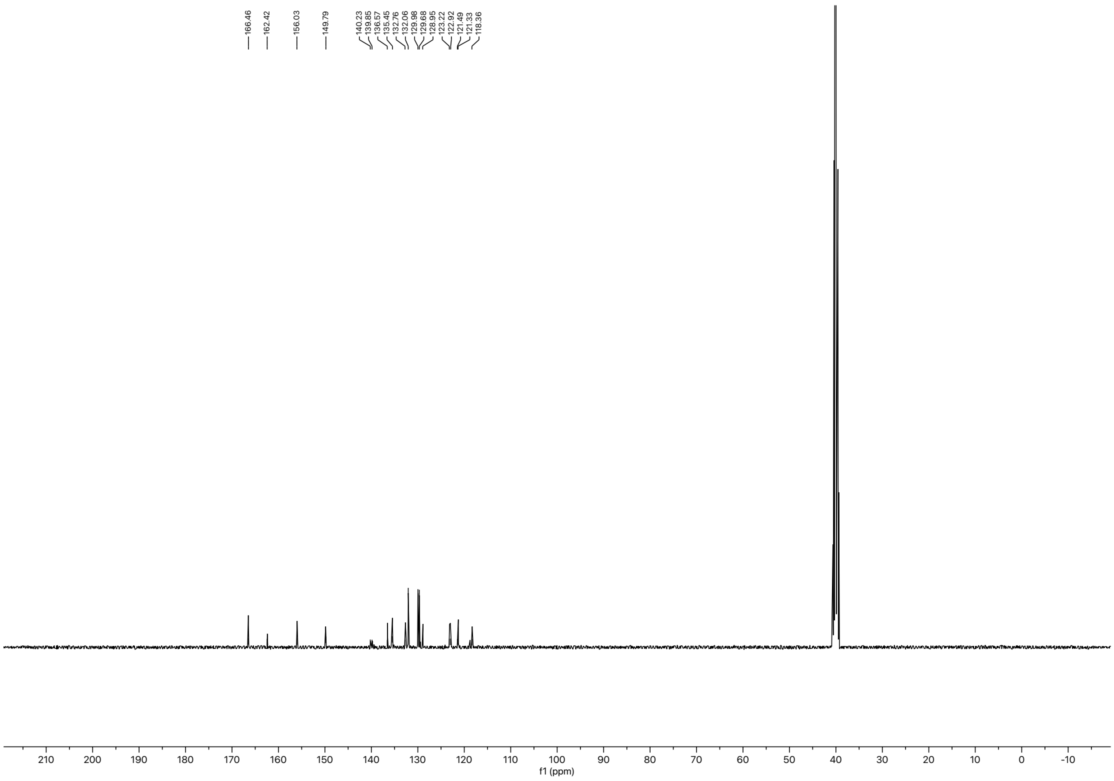

^{1}H-NMR and ^{13}C-NMR of (E)-2-chloro-5-(4-(naphthalen-2-ylmethylene)-5-oxo-3-(trifluoromethyl)-4,5- dihydro-1H-pyrazol-1-yl)benzoic acid I_{12}

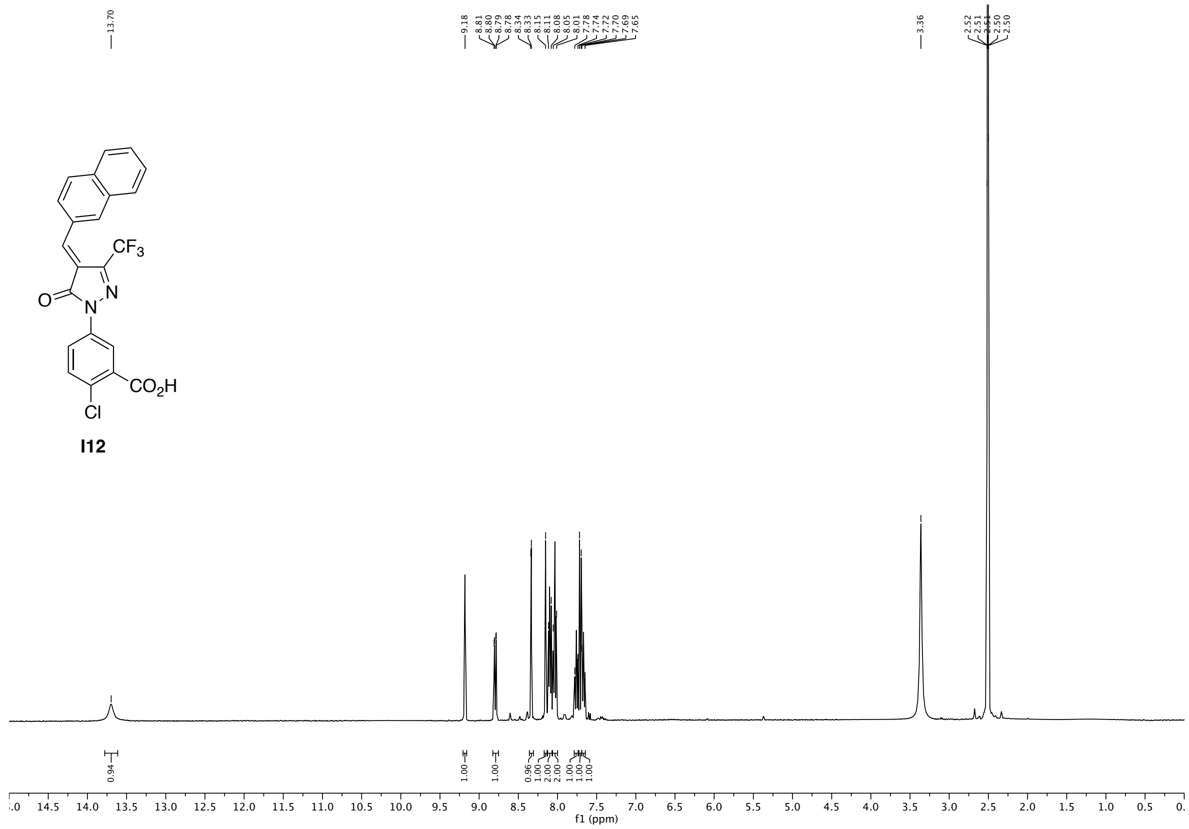

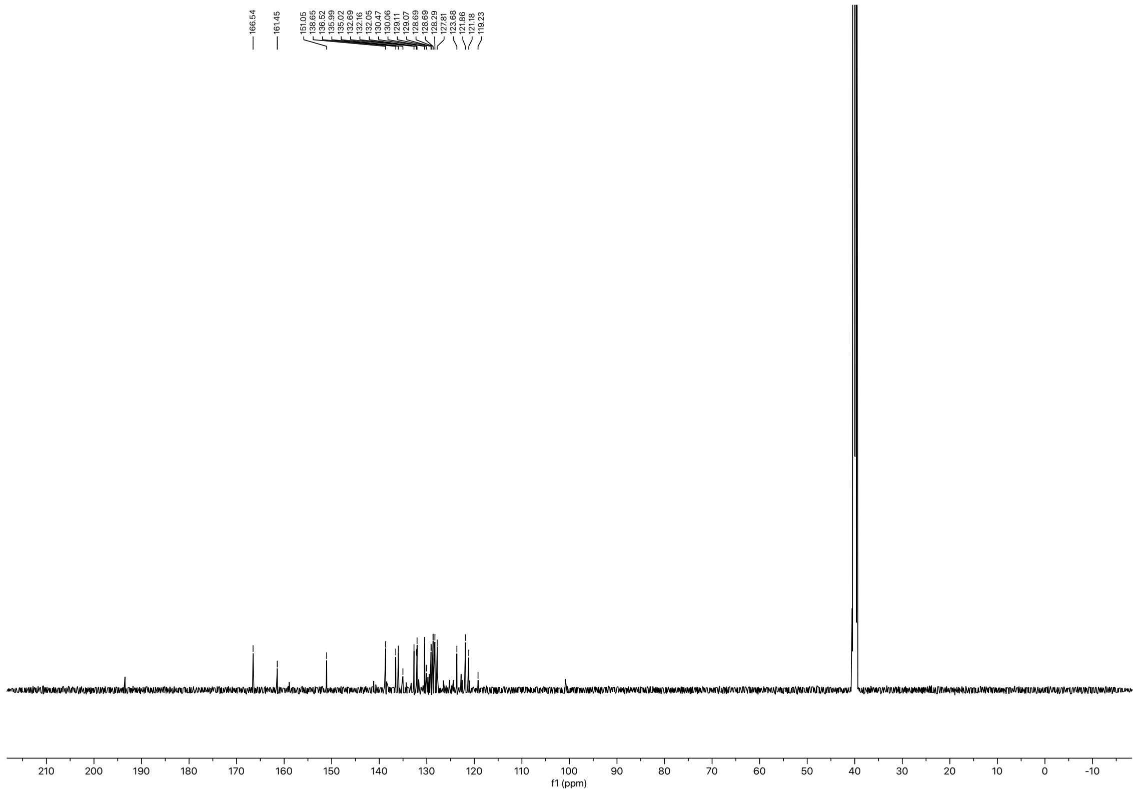

^{1}H-NMR and ^{13}C-NMR of (E)-5-(4-([1,1'-biphenyl]-4-ylmethylene)-5-oxo-3-(trifluoromethyl)-4,5- dihydro-1H-pyrazol-1-yl)-2-chlorobenzoic acid I$_{13}$

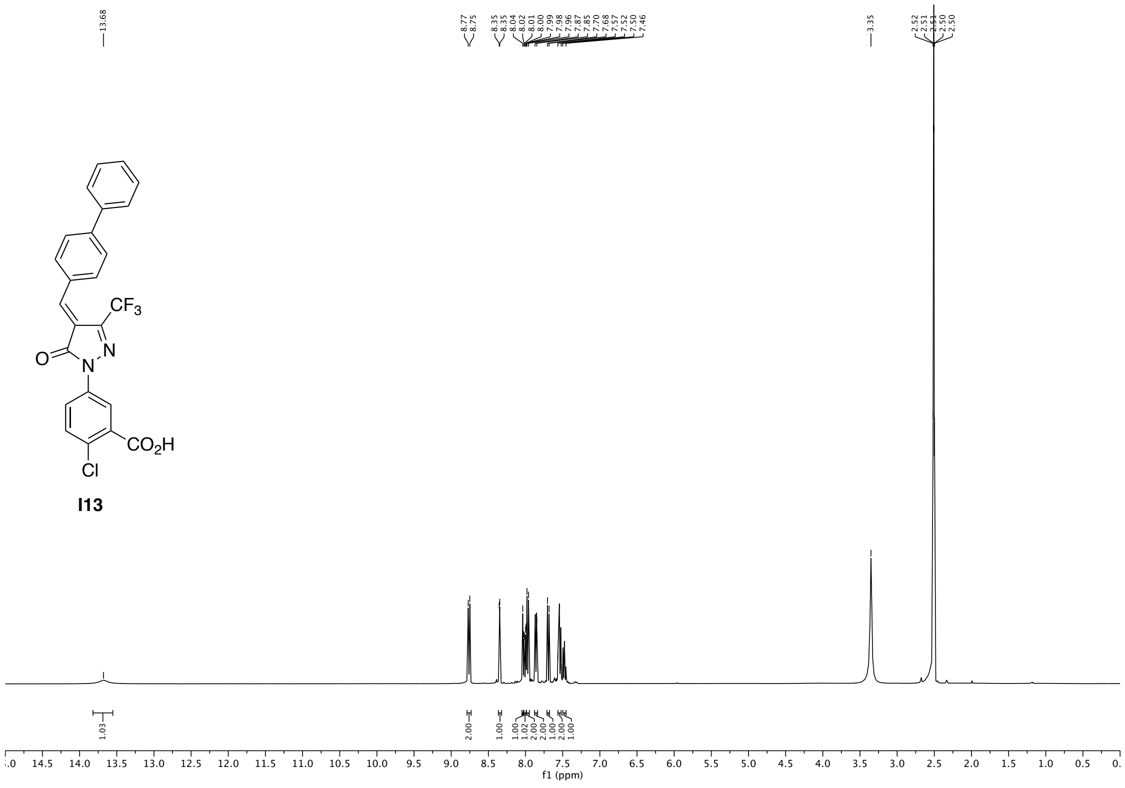

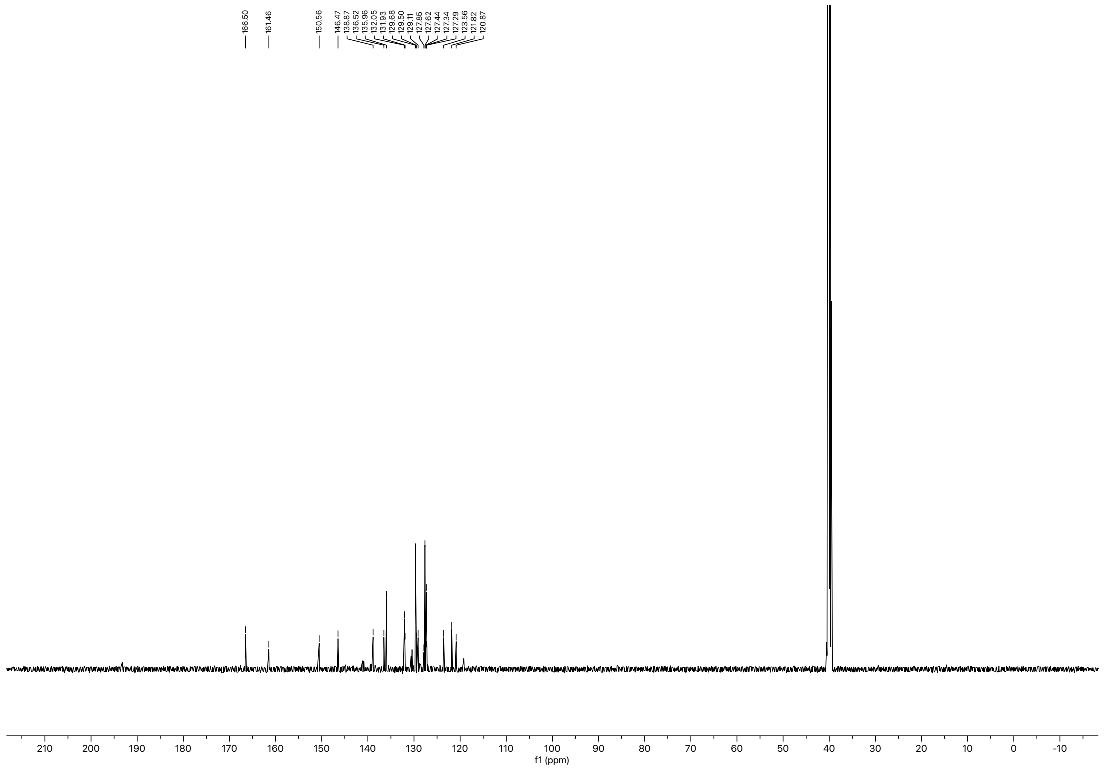

^{1}H-NMR and ^{13}C-NMR of 2-chloro-5-(3-methyl-5-oxo-4-((5-phenylfuran-2-yl)methylene)-4,5-dihydro-1H-pyrazol-1-yl)benzoic acid I_{14}

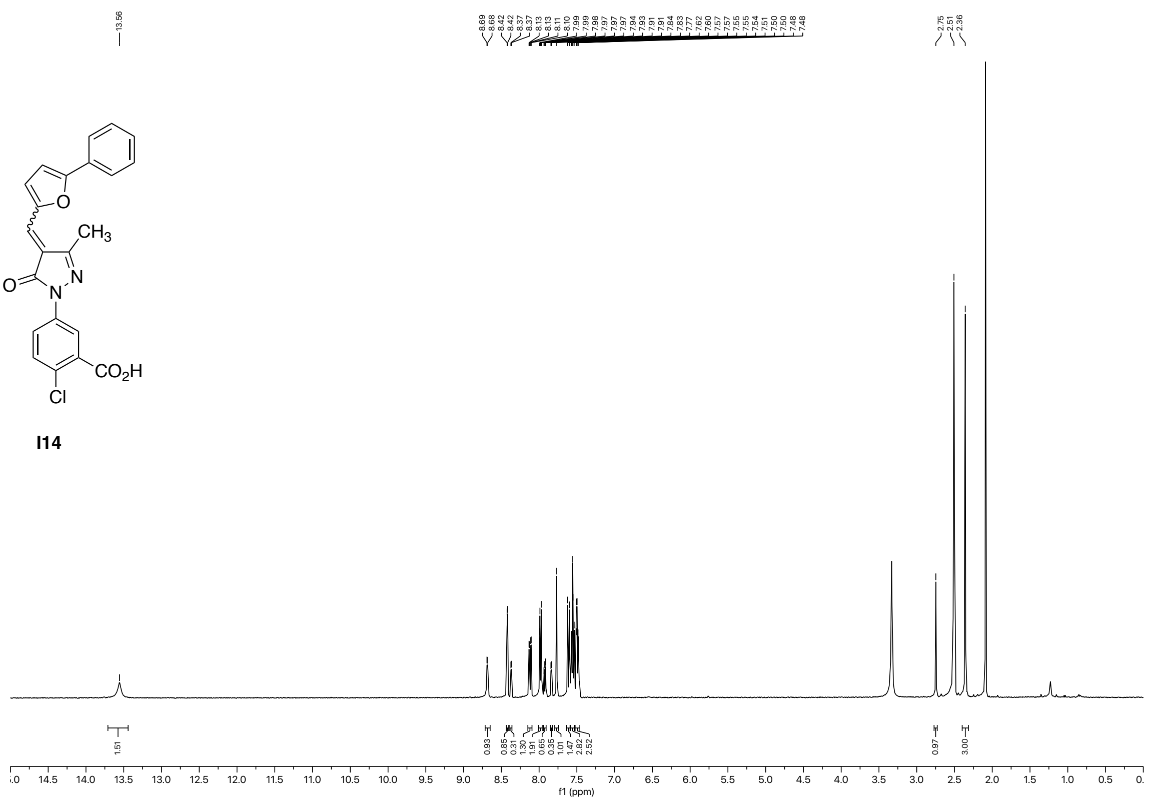

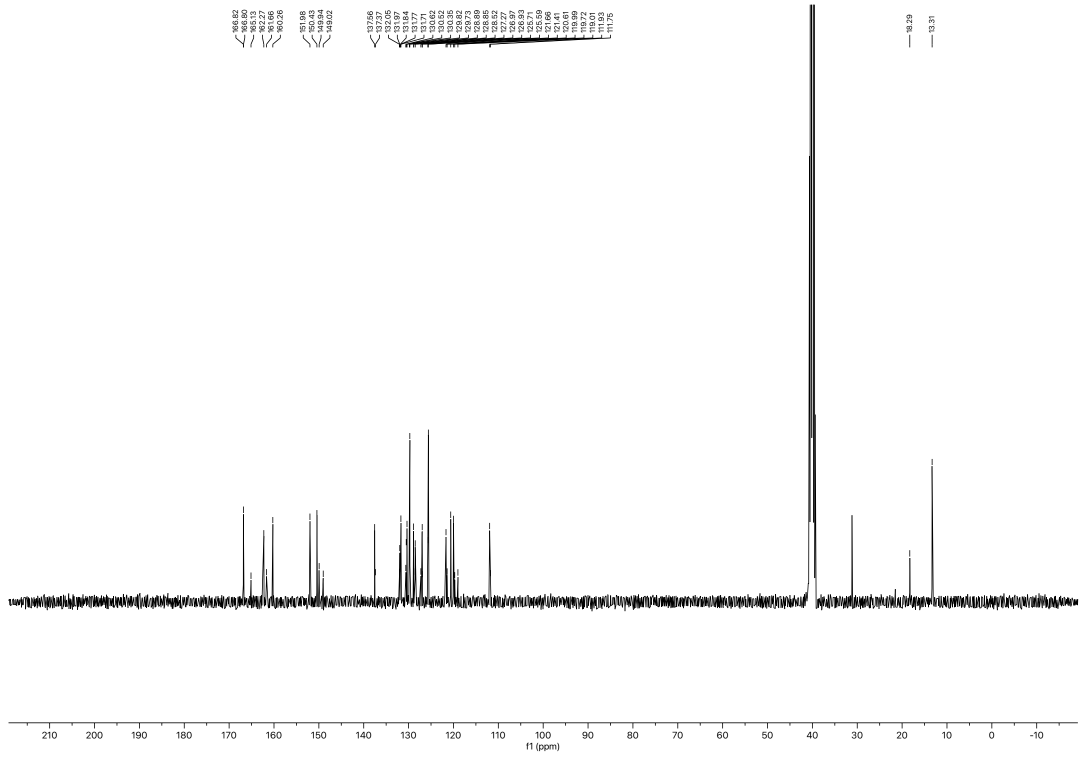

^{1}H-NMR and ^{13}C-NMR of Methyl (E)-2-chloro-5-(5-oxo-4-((5-phenylfuran-2-yl)methylene)-3 (trifluoromethyl)-4,5-dihydro-1H-pyrazol-1-yl)benzoate I_6

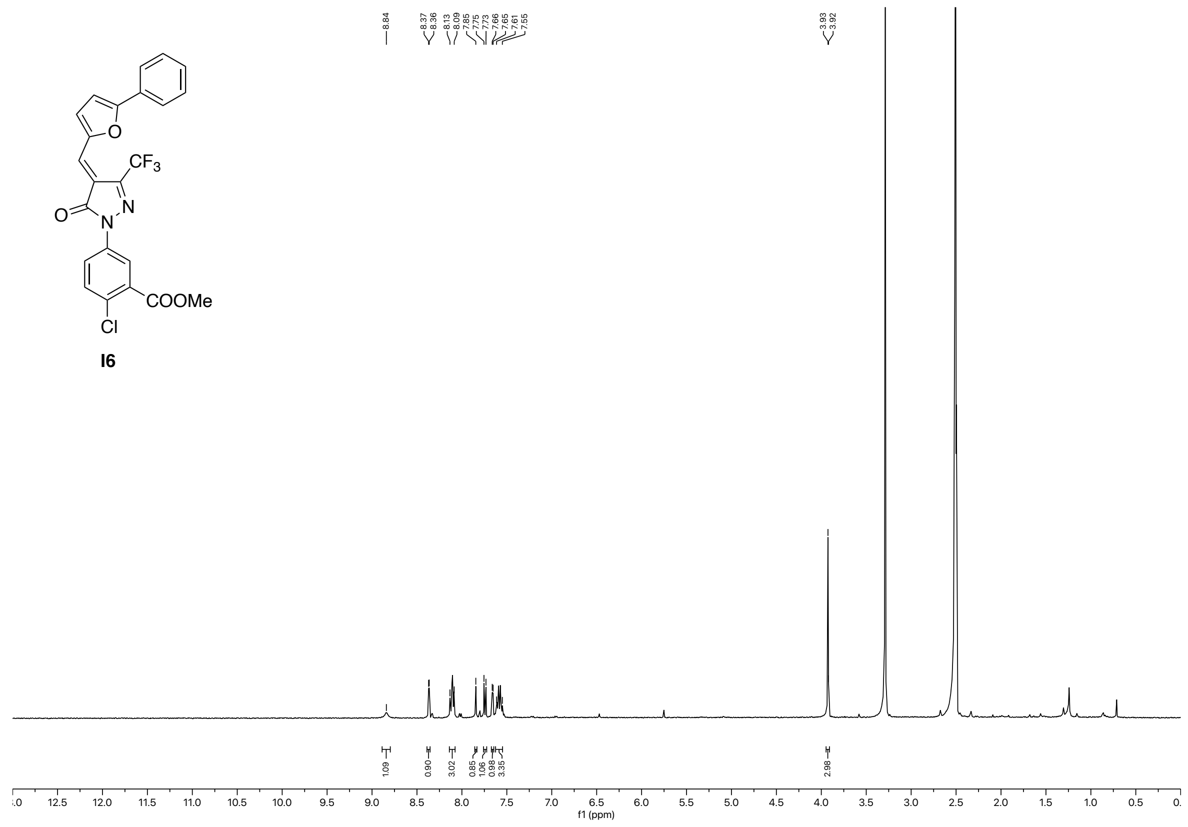

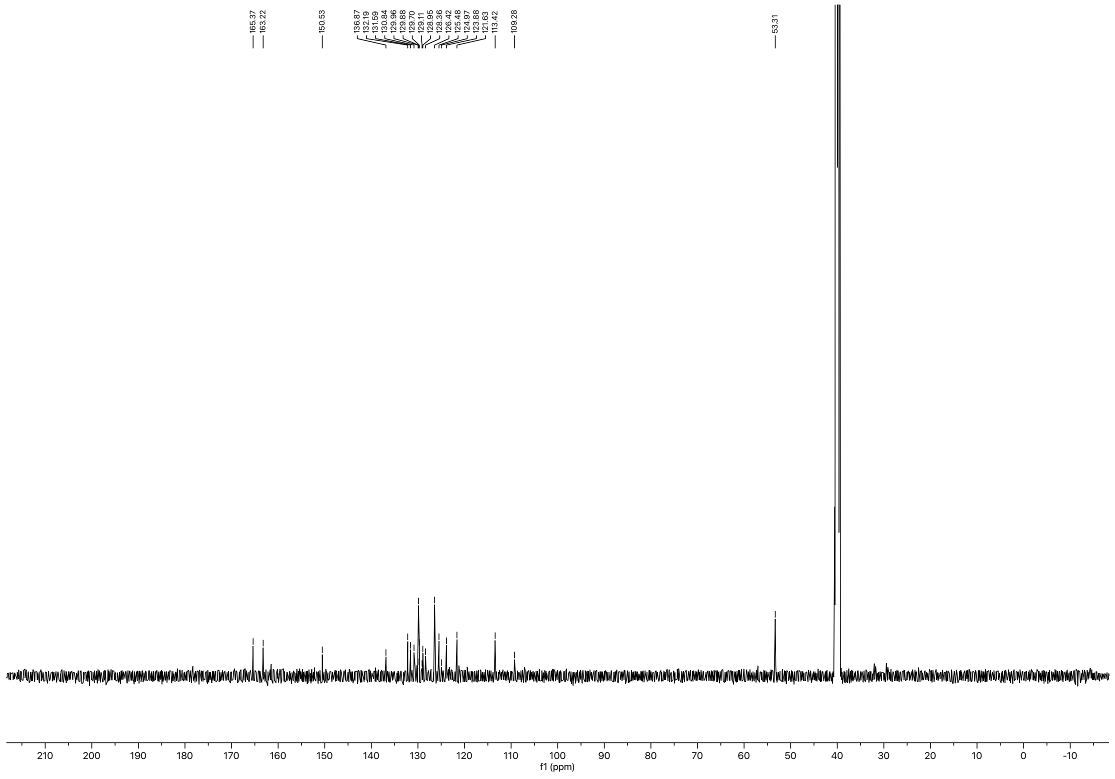

^{1}H-NMR and ^{13}C-NMR of 2-chloro-5-(5-oxo-4-((5-phenylfuran-2-yl)methyl)-3-(trifluoromethyl)-4,5-dihydro-1H-pyrazol-1-yl)benzoic acid I_{15}

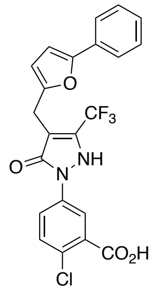

I15

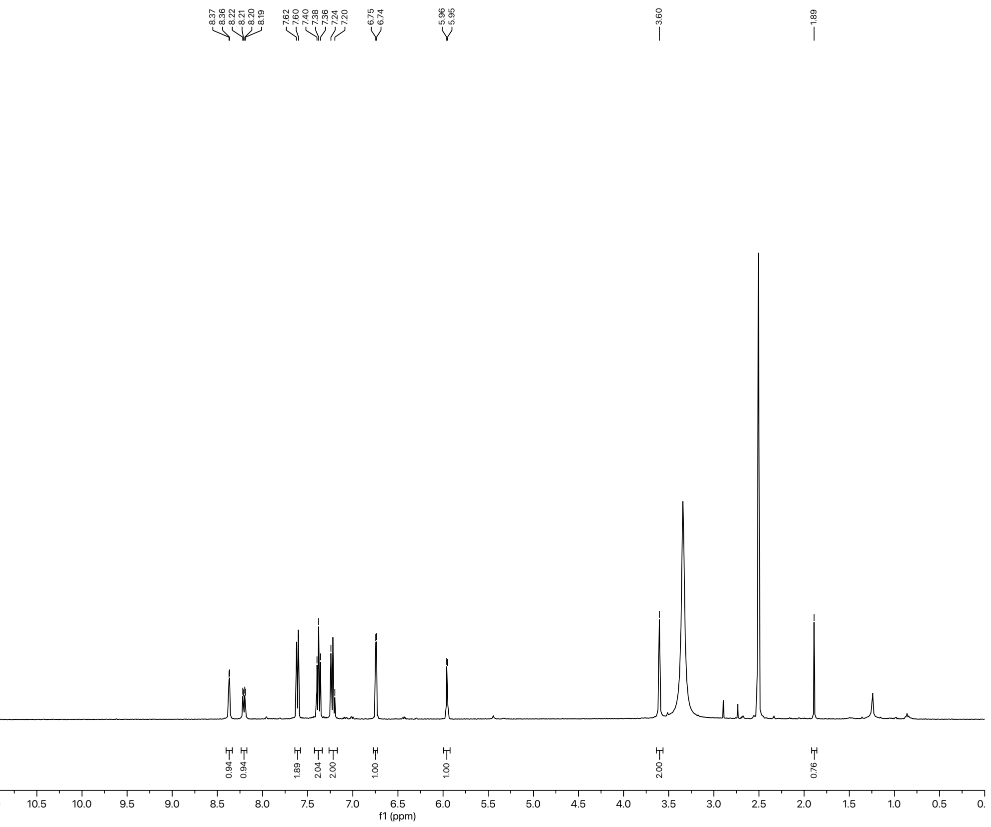

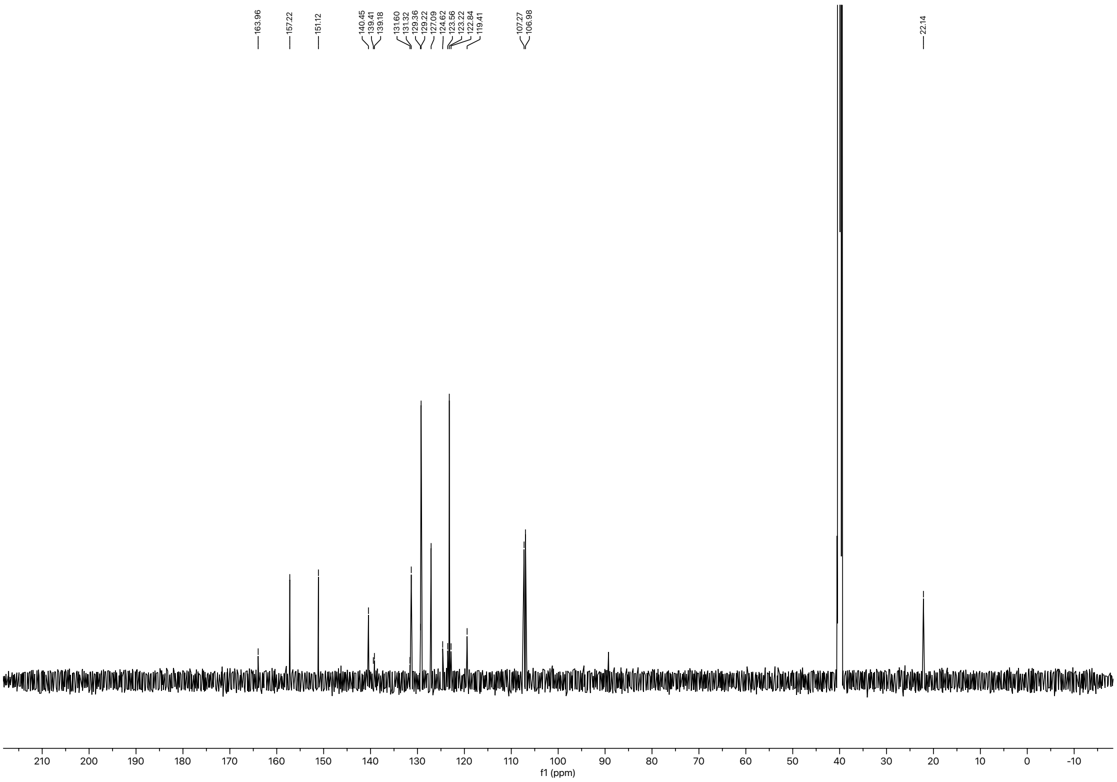

^{1}H-NMR and ^{13}C-NMR of (R)-S-(2-(2-Acetamido-3-mercaptopropanamido)ethyl) Ethane- thioate I_{16}

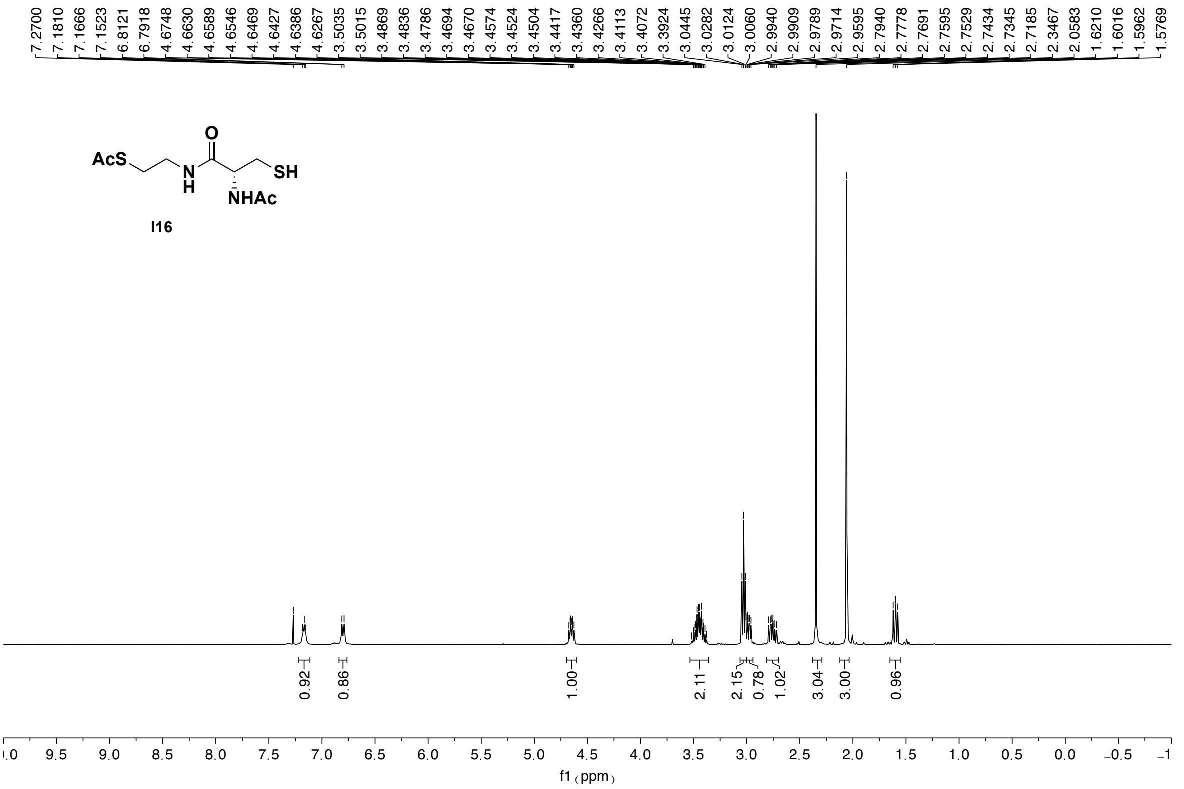

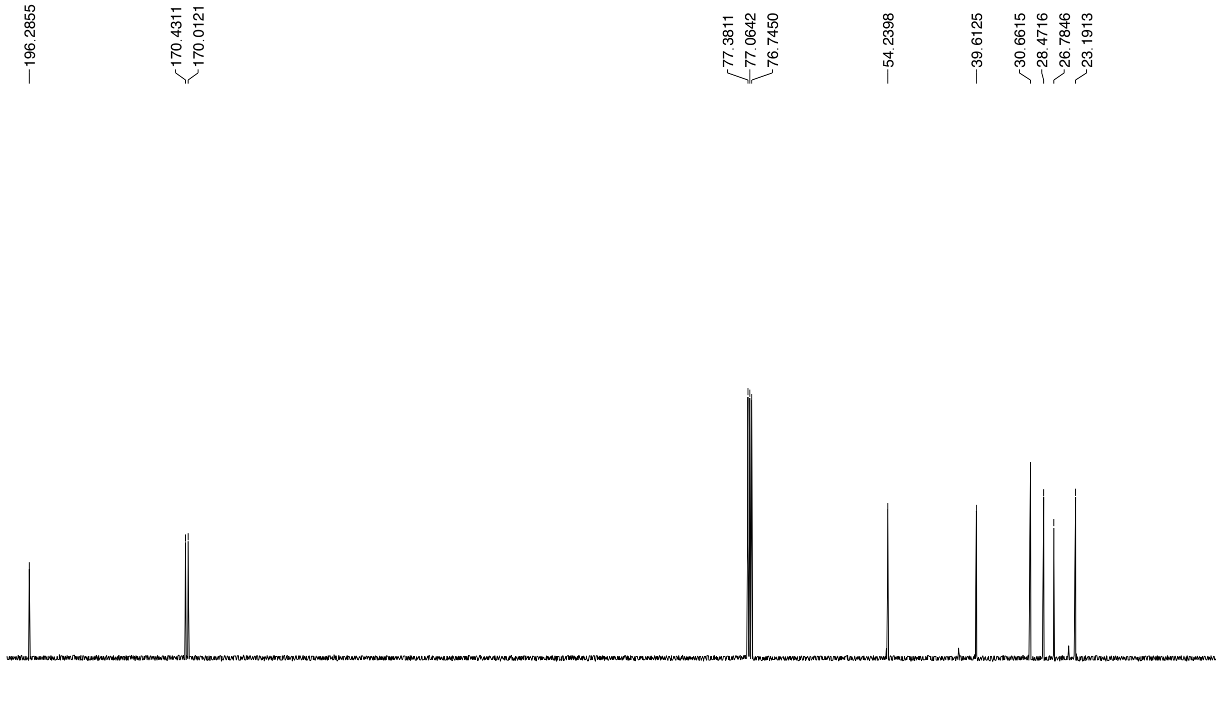

References:

[1] K. E. Hayes, P. Batsomboon, W. C. Chen, B. D. Johnson, A. Becker, S. Eschrich, Y. Yang, A. R. Robart, G. B. Dudley, W. J. Geldenhuys, L. A. Hazlehurst, Inhibition of the FAD containing ER oxidoreductin 1 (Ero1) protein by EN-460 as a strategy for the treatment of multiple myeloma, *Bioorganic & Medicinal Chemistry,* **2019**, 27,1479-1488.

[2] F. Bartoccini, M. Retini, R. Crinelli, M. Menotta, A. Fraternale, G. Piersanti, Dithiol Based on L-Cysteine and Cysteamine as a Disulfide-Reducing Agent J. Org. Chem. **2022**, 87, 10073–10079.

Figure Supplementary 1

ERO1 B expression in tumors and non-tumor tissues. Box plot of the different expression of the ERO1B gene across several types, where tumor samples are depicted in red and healthy tissues adjacent to the tumor in gray. The y-axis represents the gene expression values normalized as log2(TPM + 1). TPM stands for Transcripts Per Million. Data were sourced from the GEPIA2 platform utilizing the TCGA datasets.

Figure Supplementary 2

A) Coomassie-stained non-reducing SDS-PAGE indicating ERO1A exposed to DTT or EN460, I1, I2, I3, I6 and I15. Representative experiment reproduced twice. On the right, bar graphs indicating the reduced ERO1A on total ERO1A (mean ± SEM, One-Way ANOVA). B) ERO1A activity in presence of the indicated derivatives of EN460 measured by time-dependent AUR florescence. RFU, relative fluorescence unit.

Figure Supplementary 3

A) Bar graph representing reduced levels of ERO1 on total ERO1 (oxidized + reduced), that was arbitrarily set to 1 for the DTT-treated cells (mean ± SEM, One-Way ANOVA) (the graph in Fig.6C represents a part of this graph). B) GSH content in WT and ERO1 KO MDAMB231 cells. C) ERO1A Immunoblot representing different clones of E0771 cells subjected to a CRISPR/Cas9 procedure. Actin is a loading control. D) Non-reducing Immunoblot of endogenous ERO1 in lysates of vehicle-treated E0771 cells or exposed to DTT, EN460 and I2, as indicated. ERO1 red. stands for ERO1 reduced, ERO1 ox. for oxidized. Representative experiment reproduced twice. Below, bar graph indicating reduced levels of ERO1 on total ERO1A (oxidized +reduced), that was arbitrarily set to 1 for the DTT-treated cells. Treated samples were compared to the ctrl WT (mean ± SEM, One-way ANOVA). Ponceau stain

indicated the protein loading control. E) Analysis of E0771 apoptotic cells. E0771 were treated with I1 or EN460 or I2 for 8 hours (top panels) and 16 hours (lower panel) and later analyzed for Annexin V and propidium iodide staining by flow cytometry.

Figure Supplementary 4

PD1 in $CD4^{+}$ and $CD8^{+}$ positive lymphocytes and PD-L1 in tumor-associated macrophages (TAM) by flow cytometry analysis.

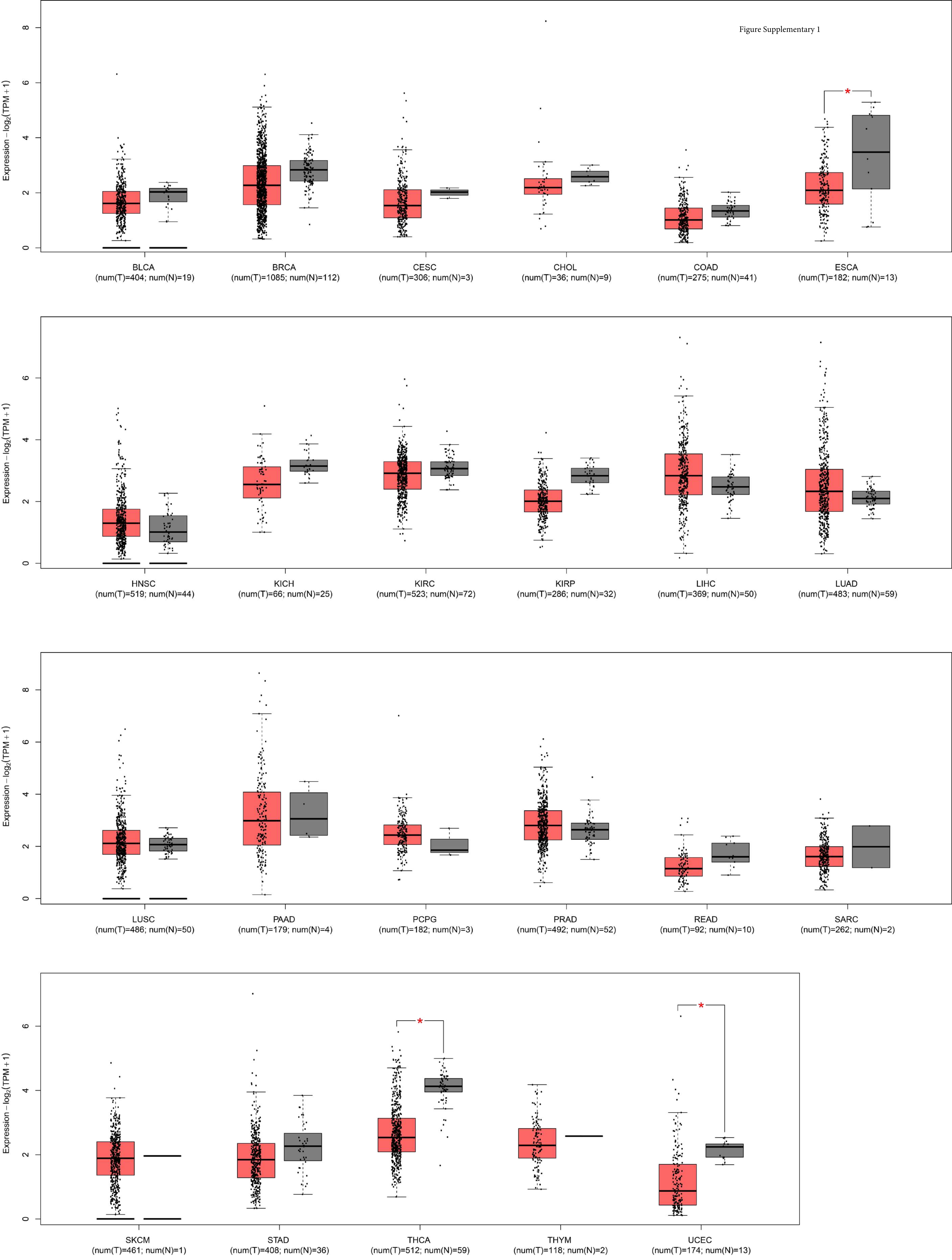
Figure Supplementary 1
Expression − log2(TPM + 1)
BLCA (num(T)=404; num(N)=19)
BRCA (num(T)=1085; num(N)=112)
CESC (num(T)=306; num(N)=3)
CHOL (num(T)=36; num(N)=9)
COAD (num(T)=275; num(N)=41)
ESCA (num(T)=182; num(N)=13)
HNSC (num(T)=519; num(N)=44)
KICH (num(T)=66; num(N)=25)
KIRC (num(T)=523; num(N)=72)
KIRP (num(T)=286; num(N)=32)
LIHC (num(T)=369; num(N)=50)
LUAD (num(T)=483; num(N)=59)
LUSC (num(T)=486; num(N)=50)
PAAD (num(T)=179; num(N)=4)
PCPG (num(T)=182; num(N)=3)
PRAD (num(T)=492; num(N)=52)
READ (num(T)=92; num(N)=10)
SARC (num(T)=262; num(N)=2)
SKCM (num(T)=461; num(N)=1)
STAD (num(T)=408; num(N)=36)
THCA (num(T)=512; num(N)=59)
THYM (num(T)=118; num(N)=2)
UCEC (num(T)=174; num(N)=13)

Figure Supplementary 2

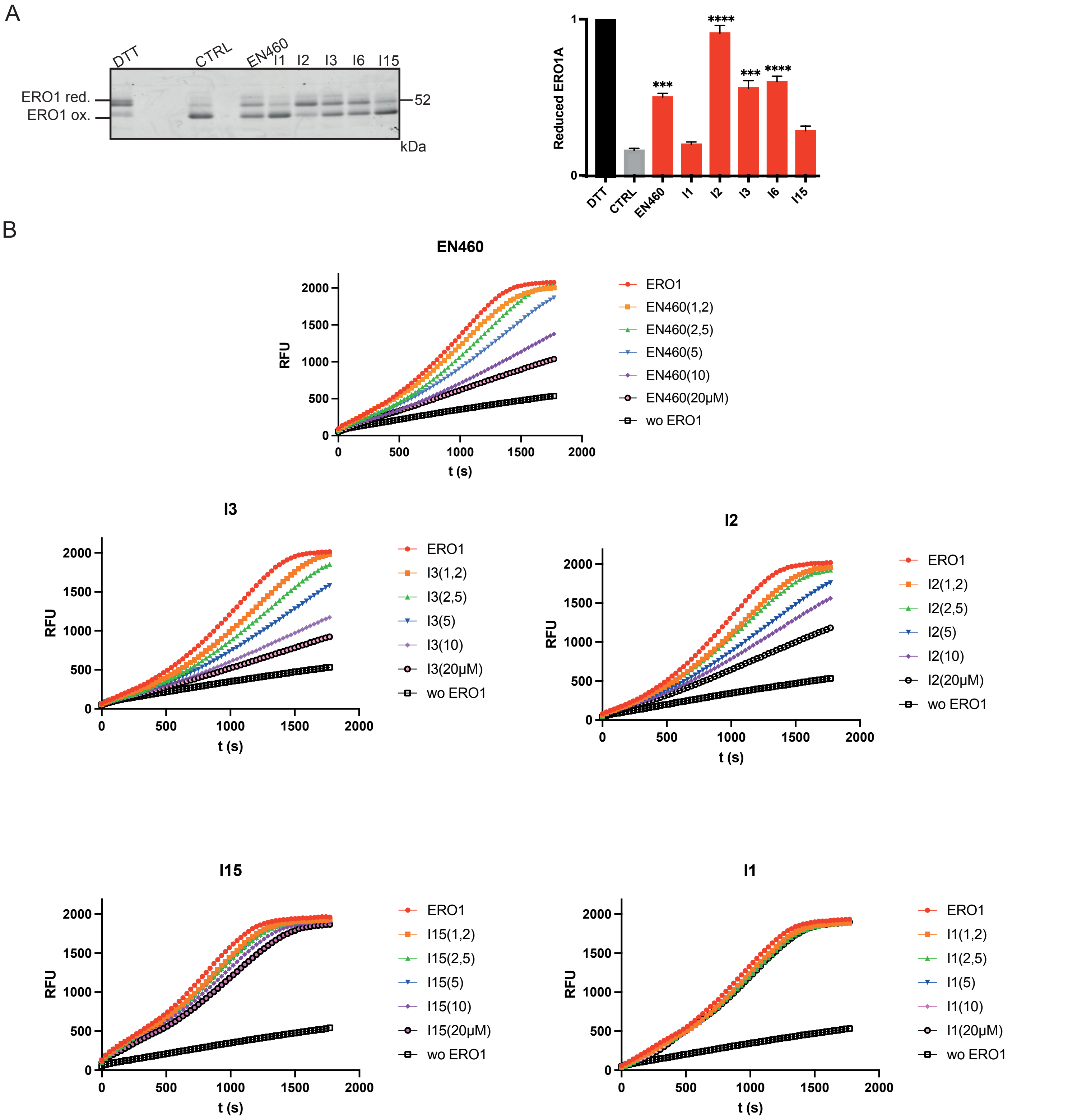

Figure Supplementary 3

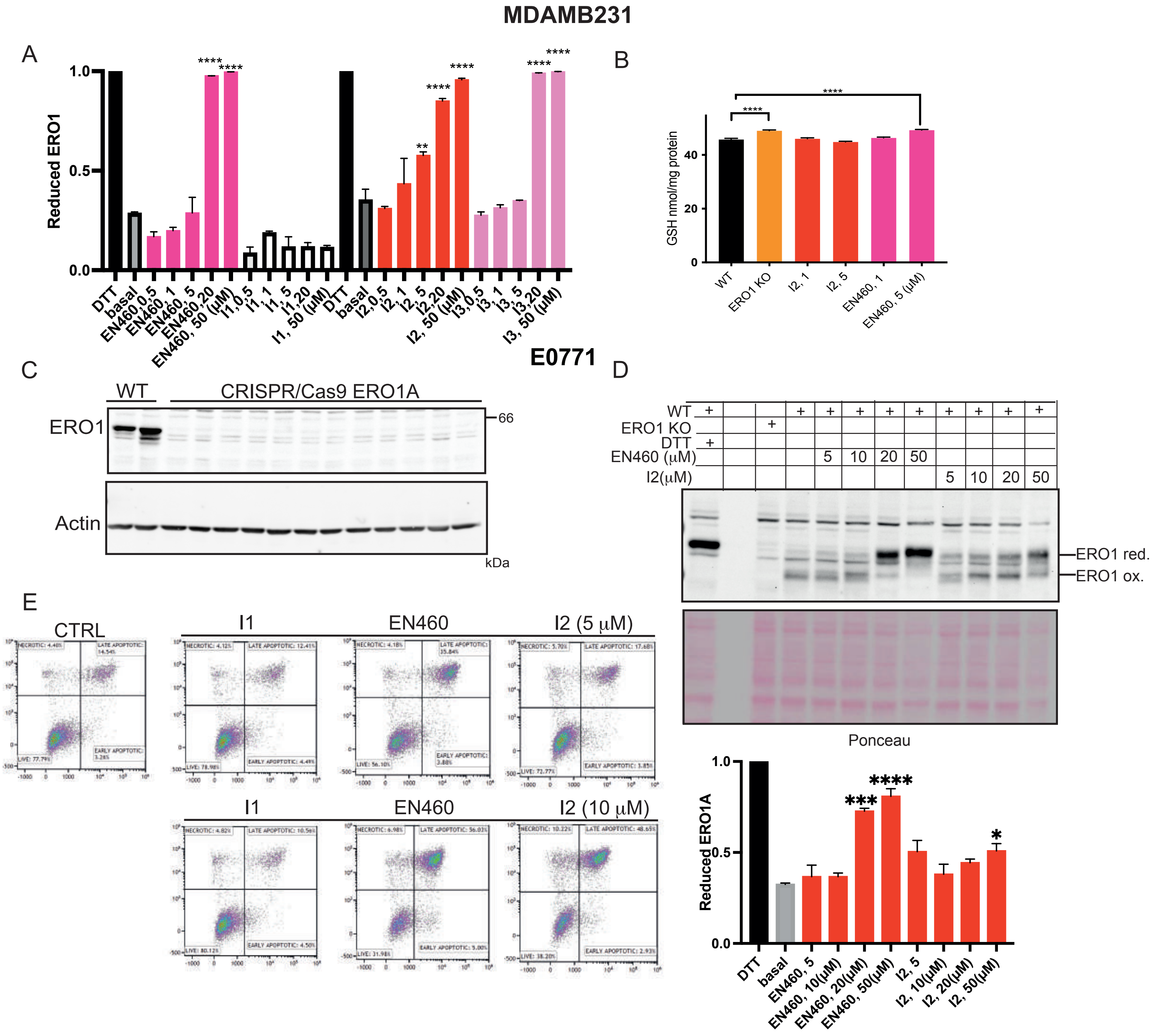

Figure Supplementary 4

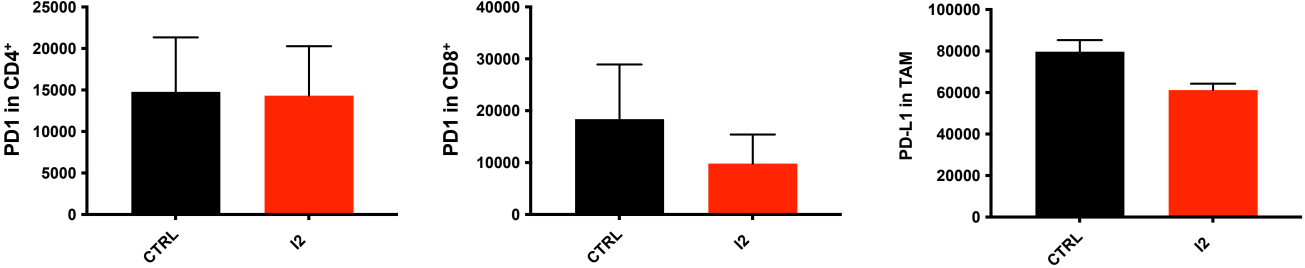

Fig.3A (1)

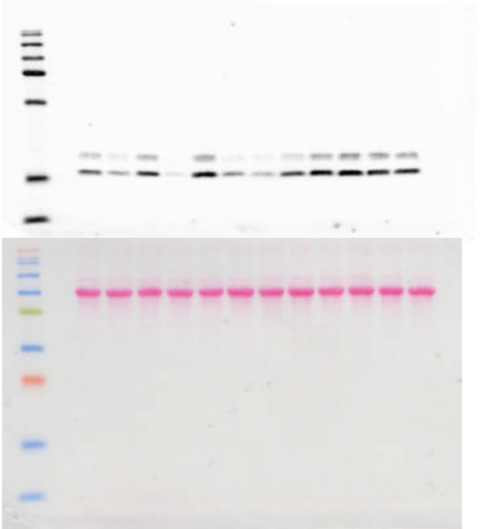

Fig.3A (2)

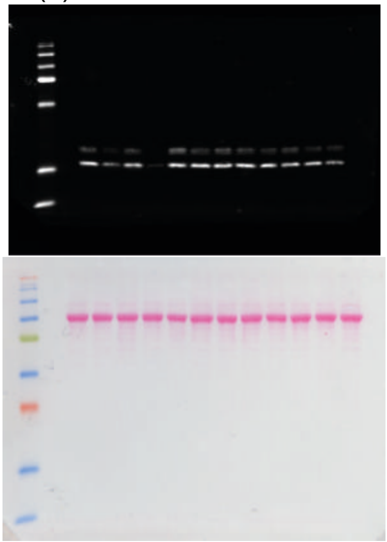

Fig.3C

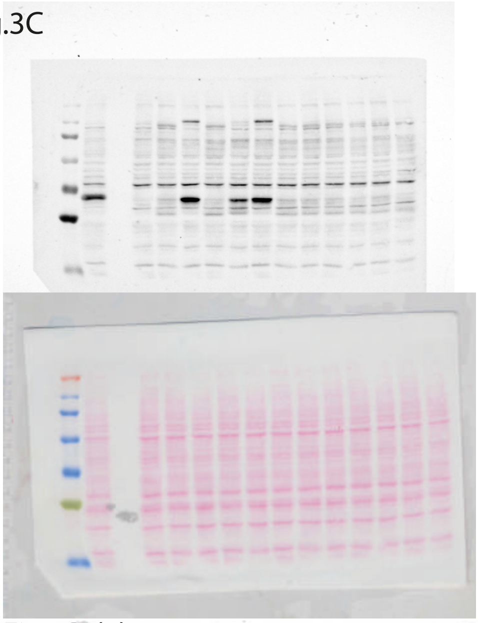

Fig.3A (3)

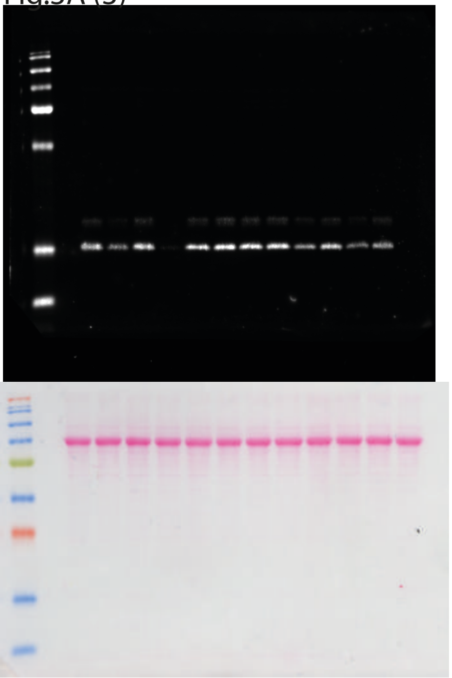

Fig.3B

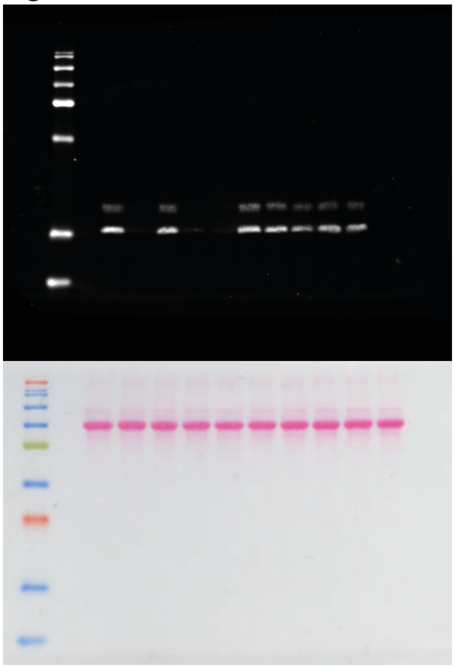

Fig.3D (1)

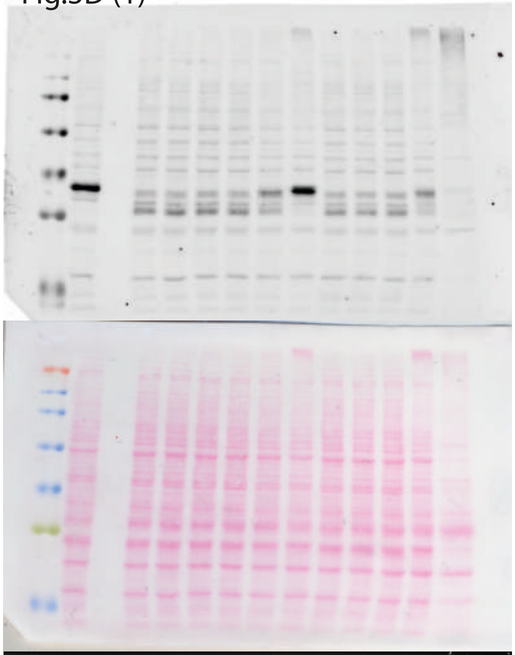

Fig.3D (2)

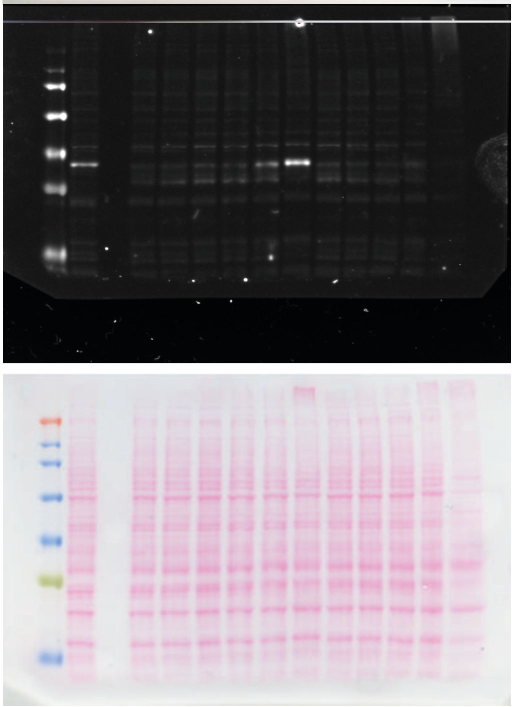

Fig.5B

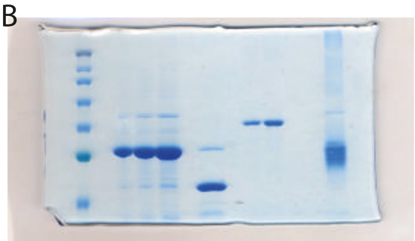

Fig.5C

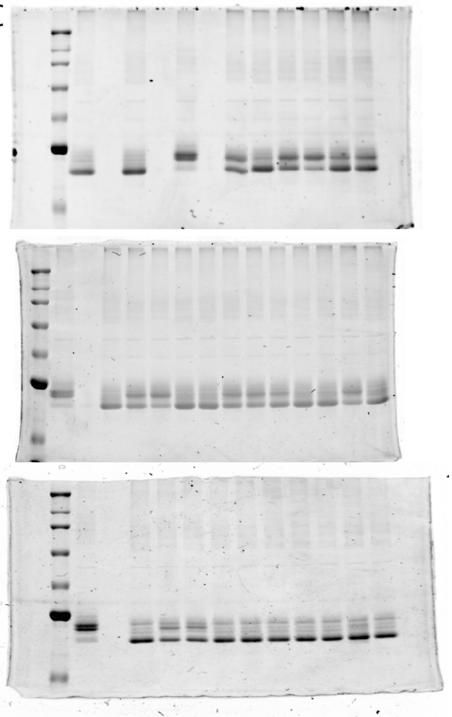

Fig.5D

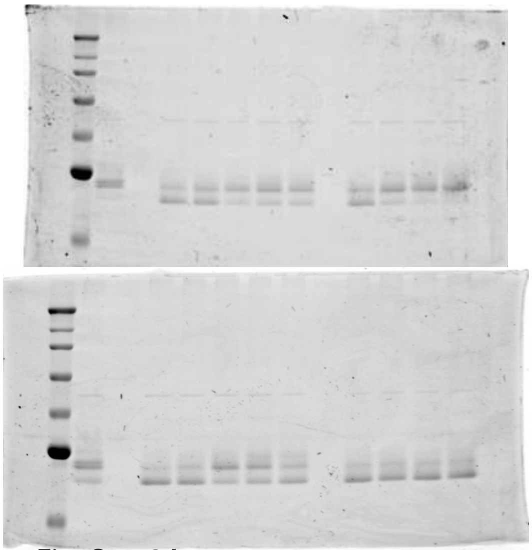

Fig.5E

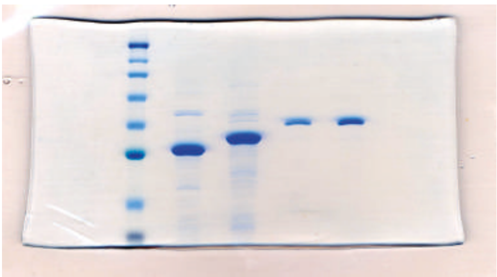

Fig.6C

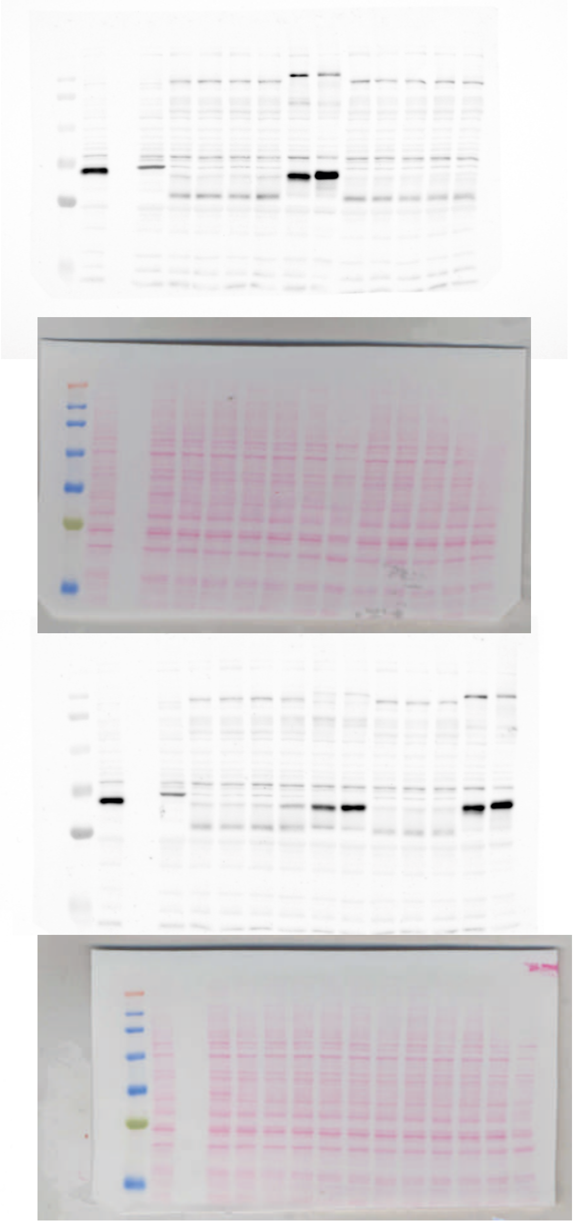

Fig. Sup 2A

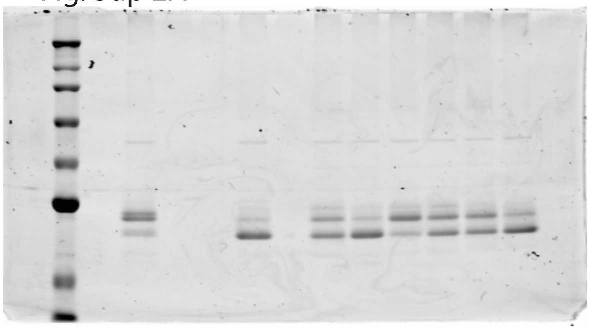

Fig. Sup 3C

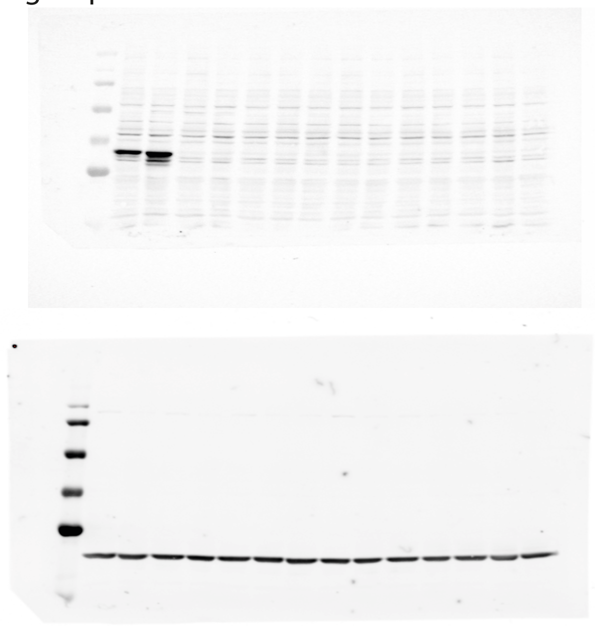

Fig. Sup 3D

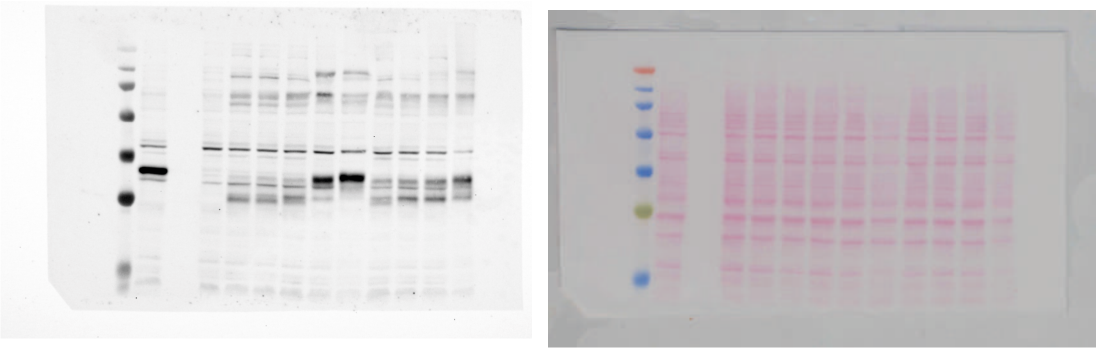

Supplement: Supplementary file 1 — Supplementary material [file 41419_2025_7426_MOESM1_ESM.pdf]
